# Asymmetric Conjugate Addition of Ketones to Maleimides Organocatalyzed by a Chiral Primary Amine-Salicylamide

**DOI:** 10.3390/molecules27196668

**Published:** 2022-10-07

**Authors:** Alejandro Torregrosa-Chinillach, Rafael Chinchilla

**Affiliations:** Department of Organic Chemistry, Faculty of Sciences, Institute of Organic Synthesis (ISO), University of Alicante, Apdo. 99, 03080 Alicante, Spain

**Keywords:** maleimides, ketones, succinimides, conjugate addition, organocatalysis, asymmetric synthesis

## Abstract

Enantioenriched substituted succinimides are interesting compounds, and their asymmetric organocatalytic synthesis by the conjugated addition of ketones to maleimides has been scarcely explored. This study shows the enantioselective conjugate addition of ketones to maleimides organocatalyzed by a simple primary amine-salicylamide derived from a chiral *trans*-cyclohexane-1,2-diamine, which provides the desired succinimides in good to excellent yields (up to 98%) and with moderate to excellent enantioselectivities (up to 99%).

## 1. Introduction

Succinimides are attractive targets in organic synthesis, as they are present in natural products and drug candidates [1,2,3,4,5,6,7] and can be transformed into other interesting compounds [8,9,10,11]. One of the most direct ways of preparing enantioenriched substituted succinimides is the organocatalytic enantioselective conjugate addition of carbon nucleophiles to maleimides [12]. Thus, using chiral organocatalysts containing tertiary amines, these carbon nucleophiles can be generated by the α-deprotonation of the acidic hydrogens of pro-nucleophiles, such as 1,3-dicarbonyl compounds [12]. The formed enolate can coordinate with the protonated amine and, if the organocatalyst bears an acidic moiety coordinating the maleimide employing a hydrogen bond, a close transition state can be produced, leading to an efficient enantioselective process. However, when aldehydes or ketones are used as pro-nucleophiles, α-deprotonation is difficult. Conjugate addition can occur by creating a transient nucleophilic enamine generated using primary or secondary amine-bearing chiral organocatalysts.

**Figure 1:**
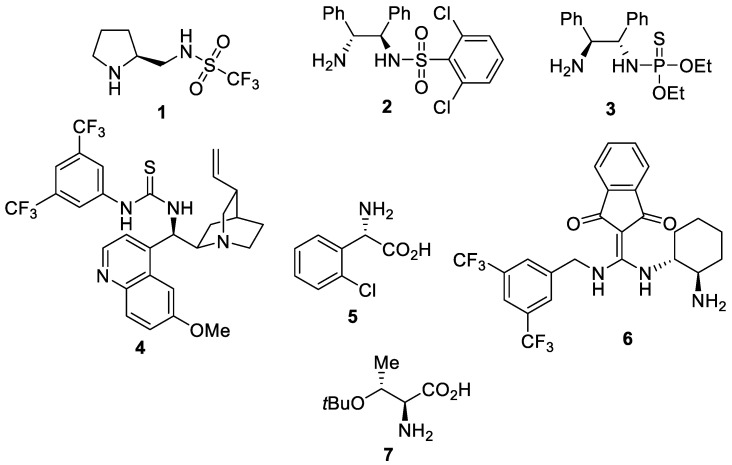
Organocatalysts employed in the asymmetric conjugate addition of ketones to maleimides.

The organocatalytic enantioselective conjugate addition reaction of aldehydes to maleimides has been profusely studied [13,14,15,16,17,18,19,20,21,22,23,24,25,26,27,28,29,30,31,32,33,34,35,36,37,38,39,40,41,42,43,44,45]. However, the same process involving ketones is challenging and has been explored by a limited number of researchers. Thus, the limited number of organocatalysts employed in this enantioselective reaction with ketones are the chiral sulfonamides **1** [46] and **2** [47] and the thiophosphoramide **3** [43] (Figure 1). In addition, the quinidine-derived thiourea **4** combined with an amino acid **5** [48], the diaminomethyleneindenedione **6** [49,50], *O*-*tert*-butyl-L-threonine (**7**) [44], and a tricomponent noncovalent organocatalytic system formed by L-isoleucine, thiourea, and potassium hydroxide [51] are used (Figure 1).

**Figure 2:**
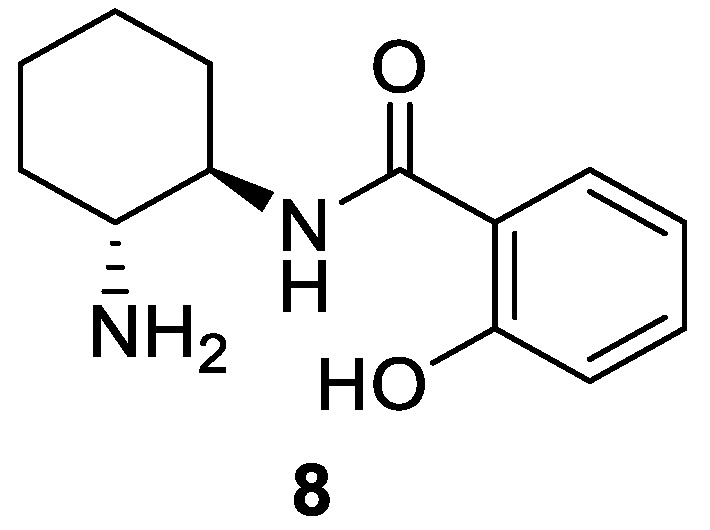
Organocatalyst employed in this study.

Recently, we have used the primary amine-salicylamide derived from chiral *trans*-cyclohexane-1,2-diamine **8** (Figure 2) as an effective organocatalyst for the enantioselective conjugate addition of aldehydes to maleimides [40,45]. We report here that this organocatalyst can be effective in the much less common asymmetric addition of ketones to maleimides.

## 2. Results and Discussion

The search for the most appropriate reaction conditions (Table 1) was carried out using the model conjugate addition reaction of cyclohexanone (**9a**) to *N*-phenylmaleimide (**1a**) (2/1 molar ratio). Thus, the reaction organocatalyzed by **8** (20 mol%) in toluene as a solvent at room temperature during 3 d afforded the corresponding substituted succinimide as a 76/24 mixture of diastereomers, the *anti*-major one **11aa** in 94% *ee*. The absolute configuration of the succinimide was determined by comparing the elution order of the corresponding four isomers in chiral HPLC with those in the literature [46]. The use of other hydrocarbons as solvents did not provide better results (Table 1, entries 2 and 3). In addition, chlorinated solvents were employed (Table 1, entries 4–6), with dichloromethane providing the best diastereoselectivity (80/20) and enantioselectivity (99% *ee*) for the main diastereomer **11aa**. On the other hand, ether solvents (Table 1, entries 7 and 8) provided considerably lower stereoselectivities, as did acetonitrile (Table 1, entry 9), alcohol solvents or water, and methanol had no reaction (Table 1, entries 10–12). Moreover, we were intrigued as to whether a longer reaction time would modify the final stereoselectivity for **11aa** when using dichloromethane as the best solvent. However, when the reaction was performed in 5 d reaction time, the final diastereo- and enantio-selectivity remained unaltered (Table 1, compare entries 4 and 13).

Next, we explore the influence of adding additives to the reaction using dichloromethane as the best solvent. Thus, adding 10 mol% of different carboxylic acids as additives had a result detrimental to the stereoselectivity (Table 1, entries 14–17). Moreover, the use of a 10 mol% of organic bases such as 4-(dimethylamino)pyridine (DMAP), imidazole, 1,8-diazabicyclo[5.4.0]undec-7-ene (DBU), or 2,6-lutidine proved ineffective (Table 1, entries 18–21). However, the use of 1,4-diazabicyclo[2.2.2]octane (DABCO) (10 mol%) as additive raised the *anti*/*syn* diastereomer ratio to 85/25, and the enantioselectivity for **11aa** was 99% (Table 1, entry 22). The loading of DABCO was increased and lowered, however this was not beneficial in any case (Table 1, entries 23 and 24). Furthermore, the reaction temperature was reduced to 5 °C while keeping DABCO (10 mol%) as an additive in dichloromethane, resulting in a diastereomeric ratio of 93/7 favoring **11aa** and a 99% *ee* (Table 1, entry 25; see Figures S1, S2 and S44 in Supplementary Materials).

**Figure 3:**
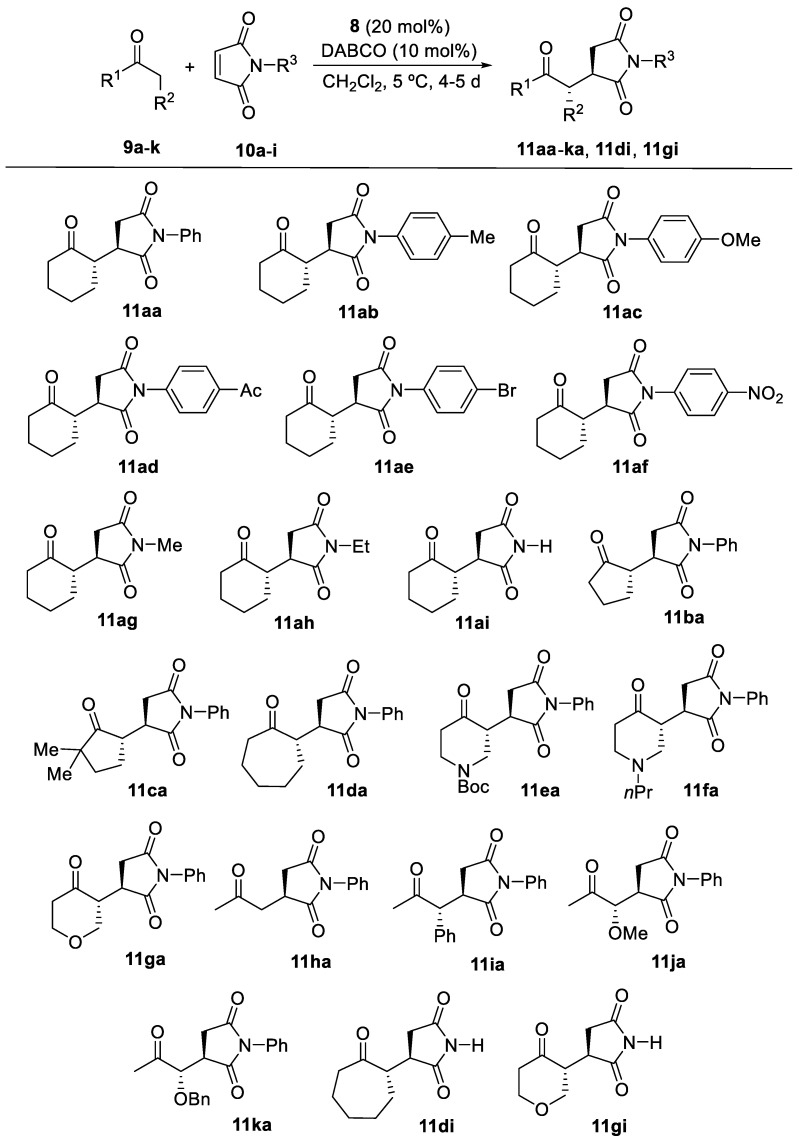
Products obtained in the conjugate addition of ketones to maleimides using **8** as organocatalyst. Only the major stereoisomer is represented; 4-O_2_NC_6_H_4_CO_2_H (10 mol%) was used as co-catalyst for adducts **11ba**, **11ca**, **11da** and **11di**. Compounds **11ba**-**11ga**, **11di** and **11gi** were obtained at room temperature.

With the optimized reaction conditions in hand [**8** (20 mol%), DABCO (10 mol%), CH_2_Cl_2_, 5 °C], we extended the procedure to other substrates (see Figure 3 and Table 2; see Figures S3–S42 and Figures S45–S84 in Supplementary Materials). We first explored the reaction of cyclohexanone (**9a**) with other *N*-substituted maleimides (**10b–h**) (Figure 3 and Table 2). Thus, electron-releasing and electron-withdrawing groups on the aromatic ring of the *N*-arylated maleimide provided the corresponding major adducts **11ab**–**af** (Figure 3) with good diastereoselectivities and with moderate enantioselectivities (Table 2, entries 2–6). In addition, when *N*-methyl or *N*-ethyl maleimide was used, the diastereoselectivity of the reaction for **11ag** and **11ah** was good, with similar enantioselectivities of 76 and 77% (Table 2, entries 7 and 8). Using the simple maleimide (**10i**) provided an excellent enantioselectivity for **11ai** (Table 2, entry 9).

We extended this conjugate addition to other ketones. Thus, five- and seven-membered cyclic ketones **9b**-**d** reacted with *N*-phenylmaleimide (**10a**) to provide the corresponding major succinimides **11ba**-**da** (Figure 3), with rather a low diastereoselectivity but with an *ee* reaching 93% for **11ca** using 2,2,dimethylcyclopentanone (**9c**) (Table 2, entries 10–12). In these three cases, the presence of an acid additive such as *p*-nitrobenzoic acid proved superior to DABCO, working at room temperature being necessary to avoid very slow reactions.

Heterocyclic ketones such as 1-Boc-piperidin-4-one (**9e**) and 1-propylpiperidin-4-one (**9f**) were used, obtaining low diastereoselectivities and moderate enantioselectivities of **11ea** and **11fa**, respectively (Table 2, entries 13 and 14). In contrast, tetrahydro-4*H*-pyran-4-one (**9g**) provided low diastereoselectivity and a very high enantioselectivity of **11ga** (Table 2, entry 15). These results were obtained working at room temperature.

Acyclic ketones were employed in the reaction with *N*-phenylmaleimide. Thus, acetone yielded succinimide **11ha** in a 72% *ee* (Table 2, entry 16), whereas 1-phenylpropan-2-one (**9i**) afforded **11ia** with very low diastereoselectivity and high enantioselectivity for **11ia** (Table 2, entry 17). In addition, the reactions with 1-methoxypropan-2-one (**9j**) and 1-phenoxypropan-2-one (**9k**) yielded moderate diastereoselectivity and adducts **11ja** and **11ka** in low and moderate enantioselectivities, respectively (Table 2, entries 18 and 19).

Finally, and considering the good results obtained using the simple maleimide **10i** as an electrophile, we explored the reaction with other ketones such as cycloheptanone (**9d**) and tetrahydro-4*H*-pyran-4-one (**9g**), obtaining the corresponding adducts **11di** and **11gi**, again with high enantioselections (Table 2, entries 20 and 21).

We scaled up the reaction leading to **11aa** using 7.4 mmol of **9a** and 3.7 mmol of **10a** instead of 0.4 mmol of **9a** and 0.2 mmol of **10a** (see Materials and Methods). The corresponding adduct **11aa** was obtained with a 97% *ee* (Table 2, entry 20).

**Figure 4:**
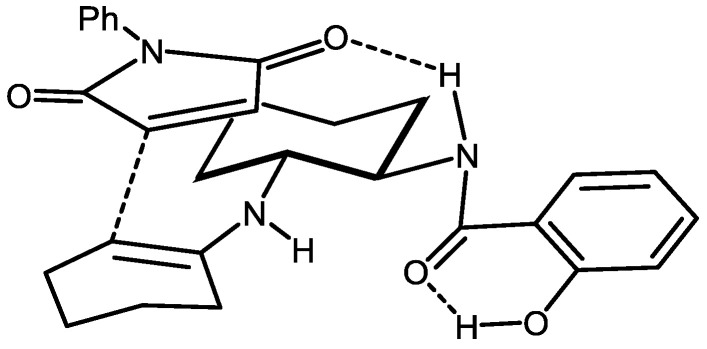
Suggested transition state leading to the formation of **11aa**.

Based on previous DFT calculations concerning the favorable transition states in the conjugated addition reaction of aldehydes to maleimides when using **8** [40], a suggested approach justifying the formation of **11aa** is depicted in Figure 4. Here, **8** would act as a bifunctional species, forming a transient enamine and at the same time coordinating one of the carbonyl groups of the maleimide through a hydrogen bond involving the amide N-H in the organocatalyst.

## 3. Materials and Methods

### 3.1. General Information

Commercially available reagents (Acros Organics, Alfa Aesar, Fluorochem, Sigma Aldrich, TCI Chemicals) were used without further purification. ^1^H NMR spectra were recorded on Bruker AV300 (300 MHz) and Bruker AV400 (400 MHz) spectrometers in proton coupled mode at room temperature. ^13^C NMR spectra were recorded on Bruker AV300 (75 MHz) and Bruker AV400 (101 MHz) spectrometers in proton decoupled mode at room temperature. Chemical shifts (*δ*) are given in parts per million (ppm). CDCl_3_ was used as solvent and tetramethylsilane (TMS) as internal standard. Coupling constants (*J*) are given in Hz. Infrared (IR) spectra were recorded with an ATR Jasco FT/IR-4100 from neat samples. Wavenumbers (*υ*) are given in cm^−1^ and the intensity is provided as very strong (vs), strong (s), weak (w), or broad (br). High resolution mass spectrometry data were obtained on an Agilent 7200 Q-TOF (EI-QTOF) and on an Agilent 1260 Chip-HPLC in line with a mass spectrometer 6500 series Q-TOF (ESI-QTOF). Thin layer chromatography (TLC) was carried out on Macherey–Nagel Alugram Sil G UV254 aluminum sheets coated with a 0.2 mm layer of silica gel. A UV light lamp (254 nm) was employed for detection. Flash column chromatography was performed using silica gel 60 of 40–63 µm (230–400 mesh) size. The *ee*’s were determined on an Agilent 1100 Series HPLC equipped with an Agilent G1311A quaternary pump and an Agilent G1315B diode array detector (DAD). The employed conditions (column, mobile phase, flow rate, wavelength) are shown in each case. Reference racemic samples of adducts **11** were obtained by performing the conjugate addition reactions using a racemic mixture of **8** and *ent*-**8** as organocatalyst at room temperature.

### 3.2. Enantioselective Michael Addition of Ketones to Maleimides: General Procedure

A mixture of organocatalyst **8** (9.4 mg, 0.04 mmol), DABCO (4.5 mg, 0.02 mmol), and **10** (0.2 mmol) was dissolved in CH_2_Cl_2_ (1 mL) in a glass vial (16 mm diameter). Then, **9** (0.4 mmol) was added and the mixture was stirred for 4 or 5 days at 5 °C (see Table 2). After this time, the solvent was evaporated under reduced pressure (15 torr) and the crude reaction mixture was purified by column chromatography (hexanes/ethyl acetate gradients), yielding adduct **11**.

*3-(2-Oxocyclohexyl)-1-phenylpyrrolidine-2,5-dione* (**11aa**) [46]. The major *anti* diastereomer was obtained combined with the minor *syn* diastereomer as an inseparable mixture in 98% global yield (*anti*/*syn* = 93/7, ^1^H NMR on the reaction crude). White solid, mp: 131–133 °C; ^1^H NMR (400 MHz, CDCl_3_): *δ* = 7.47 (tt, *J* = 8.7, 1.7 Hz, 2H), 7.41–7.28 (m, 3H), 3.34–3.13 (m, 1H), 3.11–2.78 (m, 2H), 2.67–2.51 (m, 1H), 2.40–2.28 (m, 2H), 2.15 (dddq, *J* = 20.2, 12.7, 6.7, 3.4 Hz, 2H), 1.98 (m, 1H), 1.82–1.55 (m, 3H) ppm; ^13^C NMR (101 MHz, CDCl_3_): *δ* = 210.4 (major), 210.2 (minor), 178.7 (minor), 178.5 (major), 175.9 (major), 175.8 (minor), 132.5 (major), 132.2 (minor), 129.2 (major), 128.7 (minor), 128.6 (major), 126.9 (major), 126.7 (minor), 52.3 (minor), 50.9 (major), 42.0 (minor), 41.8 (major), 41.4 (major), 40.2 (minor), 33.5 (minor), 32.1 (major), 31.9 (major), 30.4 (minor), 27.3 (minor), 27.1 (major), 25.2 (minor), 25.0 (major) ppm; HPLC: ChiralCel OD-H column, hexanes/*i*-PrOH (70/30), flow rate = 0.5 mL/min, λ = 230 nm, *t_R_* (*anti*) = 31.1 min (major enantiomer) 58.7 min (minor enantiomer), *t_R_* (*syn*) = 39.4 min (major enantiomer) 43.4 min (minor enantiomer).

*1-(4-Methylphenyl)-3-(2-oxocyclohexyl)pyrrolidine-2,5-dione* (**11ab**). The major *anti* diastereomer was obtained combined with the minor *syn* diastereomer as an inseparable mixture in 91% global yield (*anti*/*syn* = 90/10, ^1^H NMR on the reaction crude). White solid, mp: 144–146 °C; ^1^H NMR (300 MHz, CDCl_3_): *δ* = 7.30–7.25 (m, 2H), 7.24–7.17 (m, 2H), 3.34–3.15 (m, 1H), 2.68–2.53 (m, 1H), 2.48–2.27 (m, 5H, CH_3_ included), 2.23–2.07 (m, 2H), 2.03–1.93 (m, 1H), 1.86–1.52 (m, 3H) ppm; ^13^C NMR (75 MHz, CDCl_3_): *δ* = 210.4 (major), 210.2 (minor), 178.8 (minor), 178.7 (major), 176.1 (major), 176.0 (minor), 138.7 (major), 129.9 (major), 129.8 (minor), 126.7 (major), 126.5 (minor), 52.2 (minor), 50.9 (major), 42.1 (minor), 41.9 (major), 41.4 (major), 40.2 (minor), 33.4 (minor), 32.2 (major), 31.9 (major), 30.2 (minor), 29.8 (minor), 27.3 (minor), 27.1 (major), 25.2 (minor), 25.1 (major), 21.4 (major) ppm; IR (neat): *υ* = 2939 (w), 2862 (w), 1778 (w), 1697 (vs), 1647 (w), 1516 (w), 1400 (w), 1200 (w), 1122 (w), 1041 (w), 818 (w), 768 (w), 663 (w) cm^−1^; MS (70 eV, EI): *m*/*z* (%): 67.2 (17), 107.2 (49), 133.1 (29), 150.1 (15), 189.1 (100), 228.1 (22), 285.1 (M^+^, 79); HRMS (EI-QTOF) calcd. for C_17_H_19_NO_3_ (*M*^+^): 285.1365. Found: 285.1357; HPLC: ChiralPak AD-H column, hexanes/*i*-PrOH (70/30), flow rate = 1.0 mL/min, λ = 254 nm, *t_R_* (*anti*) = 20.70 min (major enantiomer) 17.44 min (minor enantiomer), *t_R_* (*syn*) = 22.49 min (major enantiomer) 19.42 min (minor enantiomer).

*1-(4-Methoxyphenyl)-3-(2-oxocyclohexyl)pyrrolidine-2,5-dione* (**11ac**). The major *anti* diastereomer was obtained combined with the minor *syn* diastereomer as an inseparable mixture in 90% global yield (*anti*/*syn* = 85/15, ^1^H NMR on the reaction crude). Yellow solid, mp: 120–122 °C; ^1^H NMR (400 MHz, CDCl_3_): *δ* = 7.28–7.19 (m, 2H), 7.00–6.95 (m, 2H), 3.81 (s, 3H), 3.31–3.12 (m, 1H), 3.10–2.76 (m, 2H), 2.67–2.50 (m, 1H), 2.48–2.27 (m, 2H), 2.21–2.03 (m, 2H), 2.02–1.94 (m, 1H), 1.81–1.55 (m, 3H) ppm; ^13^C NMR (101 MHz, CDCl_3_): *δ* = 210.4 (major), 201.2 (minor), 178.9 (minor), 178.8 (major), 176.2 (major), 176.1 (minor), 159.6 (major), 128.1 (major), 127.9 (minor), 125.1 (major), 124.9 (minor), 114.6 (major), 55.6 (major), 52.3 (minor), 50.9 (major), 42.0 (minor), 41.8 (major), 41.3 (major), 40.1 (minor), 33.4 (minor), 32.1 (major), 31.9 (major), 30.3 (minor), 29.8 (minor), 27.3 (minor), 27.1 (major), 25.1 (minor), 25.0 (major) ppm; IR (neat): *υ* = 2931 (w), 2858 (w), 1778 (w), 1697 (vs), 1512 (s), 1396 (w), 1246 (s), 1184 (s), 1026 (w), 833 (w), 663 (w) cm^−1^; MS (70 eV, EI): *m*/*z* (%): 123.1 (46), 205.1 (45), 244.1 (22), 301.1 (M^+^, 100); HRMS (EI-QTOF) calcd. for C_17_H_19_NO_4_ (*M*^+^): 301.1314. Found: 301.1308; HPLC: ChiralPak AD-H column, hexanes/*i*-PrOH (70/30), flow rate = 0.8 mL/min, λ = 240 nm, *t_R_* (*anti*) = 34.38 min (major enantiomer) 29.47 min (minor enantiomer), *t_R_* (*syn*) = 41.26 min (major enantiomer) 45.35 min (minor enantiomer).

*1-(4-Acetylphenyl)-3-(2-oxocyclohexyl)pyrrolidine-2,5-dione* (**11ad**). The major *anti* diastereomer was obtained combined with the minor *syn* diastereomer as an inseparable mixture in 82% global yield (*anti*/*syn* = 88/12, ^1^H NMR on the reaction crude). White solid, mp: 128–130 °C; ^1^H NMR (400 MHz, CDCl_3_): *δ* = 8.12–8.00 (m, 2H), 7.56–7.42 (m, 2H), 3.33–3.13 (m, 1H), 3.12–2.79 (m, 2H), 2.76–2.53 (m, 4H, CH_3_ included), 2.52–2.30 (m, 2H), 2.25–2.08 (m, 2H), 2.06–1.96 (m, 1H), 1.85–1.56 (m, 3H) ppm; ^13^C NMR (101 MHz, CDCl_3_): *δ* = 210.5 (major), 210.3 (minor), 197.3 (major), 137.2 (minor), 178.2 (minor), 178.1 (major), 175.4 (major), 175.2 (minor), 136.7 (major), 136.6 (major), 136.4 (minor), 129.2 (major), 126.8 (major), 126.7 (minor), 52.6 (minor), 51.0 (major), 42.0 (minor), 41.8 (major), 41.4 (major), 40.4 (minor), 33.8 (minor), 32.2 (major), 31.9 (major), 30.9 (minor), 29.8 (minor), 27.3 (minor), 27.1 (major), 26.8 (major), 25.2 (minor), 25.0 (major) ppm; IR (neat): *υ* = 2931 (w), 2866 (w), 1782 (w), 1697 (vs), 1601 (w), 1512 (w), 1400 (s), 1257 (w), 1192 (s), 1126 (w), 957 (w), 837 (w), 744 (w), 663 (w) cm^−1^; MS (70 eV, EI): *m*/*z* (%): 97.1 (15), 120.1 (22), 146.1 (21), 202.1 (25), 217.1 (100), 298.1 (65), 313.1 (M^+^, 54); HRMS (EI-QTOF) calcd. for C_18_H_19_NO_4_ (*M*^+^): 313.1314. Found: 313.1308; HPLC: ChiralPak AD-H column, hexanes/*i*-PrOH (70/30), flow rate = 0.8 mL/min, λ = 230 nm, *t_R_* (*anti*) = 58.77 min (major enantiomer) 53.11 min (minor enantiomer), *t_R_* (*syn*) = 82.26 min (major enantiomer) 181.91 min (minor enantiomer).

*1-(4-Bromophenyl)-3-(2-oxocyclohexyl)pyrrolidine-2,5-dione* (**11ae**) [46]. The major *anti* diastereomer was obtained combined with the minor *syn* diastereomer as an inseparable mixture in 95% global yield (*anti*/*syn* = 92/8, ^1^H NMR on the reaction crude). White solid, mp: 148–150 °C; ^1^H NMR (300 MHz, CDCl_3_): *δ* = 7.64–7.54 (m, 2H), 7.27–7.18 (m, 2H), 3.33–3.09 (m, 1H), 3.10–2.74 (m, 2H), 2.67–2.47 (m, 1H), 2.46–2.25 (m, 2H), 2.22–2.06 (m, 2H), 2.03–1.92 (m, 1H), 1.85–1.50 (m, 3H) ppm; ^13^C NMR (75 MHz, CDCl_3_): *δ* = 210.5 (major), 210.3 (minor), 178.3 (minor), 178.2 (major), 175.5 (major), 175.4 (minor), 132.4 (major), 131.5 (major), 131.3 (minor), 128.5 (major), 128.3 (minor), 122.5 (major), 52.6 (minor), 51.0 (major), 42.1 (minor), 41.8 (major), 41.4 (major), 40.3 (minor), 33.7 (minor), 32.1 (major), 31.9 (major), 30.9 (minor), 27.3 (minor), 27.1 (major), 25.2 (minor), 25.0 (major) ppm; HPLC: ChiralCel OD-H column, hexanes/*i*-PrOH (70/30), flow rate = 0.5 mL/min, λ = 230 nm, *t_R_* (*anti*) = 40.14 min (major enantiomer) 63.08 min (minor enantiomer), *t_R_* (*syn*) = 46.56 min (major enantiomer) 52.85 min (minor enantiomer).

*1-(4-Nitrophenyl)-3-(2-oxocyclohexyl)pyrrolidine-2,5-dione* (**11af**). The major *anti* diastereomer was obtained combined with the minor *syn* diastereomer as an inseparable mixture in 80% global yield (*anti*/*syn* = 84/16, ^1^H NMR on the reaction crude). Yellow solid, mp: 116–118 °C; ^1^H NMR (400 MHz, CDCl_3_): *δ* = 8.39–8.28 (m, 2H), 7.64–7.57 (m, 2H), 3.40–3.16 (m, 1H), 3.15–2.83 (m, 2H), 2.72–2.54 (m, 1H), 2.52–2.30 (m, 2H), 2.26–2.11 (m, 2H), 2.07–1.96 (m, 1H), 1.87–1.56 (m, 3H) ppm; ^13^C NMR (101 MHz, CDCl_3_): *δ* = 210.87 (major), 210.5 (minor), 177.8 (minor), 177.7 (major), 175.0 (major), 174.8 (minor), 147.4 (minor), 147.1 (major), 138.1 (major), 137.9 (minor), 127.4 (major), 127.3 (minor), 124.6 (minor), 124.4 (major), 53.0 (minor), 21.1 (major), 42.1 (minor), 41.7 (major), 41.4 (major), 40.5 (minor), 34.1 (minor), 32.1 (major), 31.8 (major), 31.5 (minor), 29.8 (major), 27.4 (minor), 27.0 (major), 25.3 (minor), 25.0 (major) ppm; IR (neat): *υ* = 2931 (w), 2862 (w), 1786 (w), 1705 (vs), 1647 (w), 1523 (s), 1389 (w), 1342 (s), 1184 (s), 849 (w), 690 (w) cm^−1^; MS (70 eV, EI): *m*/*z* (%): 55.1 (32), 68.1 (22), 97.1 (100), 220.1 (37), 316.1 (M^+^, 17); HRMS (EI-QTOF) calcd. for C_16_H_16_N_2_O_5_ (*M*^+^): 316.1059. Found: 316.1041; HPLC: ChiralPak AD-H column, hexanes/*i*-PrOH (80/20), flow rate = 0.6 mL/min, λ = 254 nm, *t_R_* (*anti*) = 146.02 min (major enantiomer) 95.57 min (minor enantiomer), *t_R_* (*syn*) = 156.09 min (major enantiomer) 174.45 min (minor enantiomer).

*1-Methyl-3-(2-oxocyclohexyl)pyrrolidine-2,5-dione* (**11ag**) [43]. The major *anti* diastereomer was obtained combined with the minor *syn* diastereomer as an inseparable mixture in 94% global yield (*anti*/*syn* = 91/9, ^1^H NMR on the reaction crude). White solid, mp: 90–92 °C; ^1^H NMR (400 MHz, CDCl_3_): *δ* = 3.21–3.08 (m, 1H), 3.02 (s, 3H), 2.98–2.63 (m, 2H), 2.52–2.42 (m, 1H), 2.41–2.27 (m, 2H), 2.14 (dddq, *J* = 19.4, 12.1, 6.2, 3.1 Hz, 2H), 2.01–1.92 (m, 1H), 1.81–1.51 (m, 3H) ppm; ^13^C NMR (101 MHz, CDCl_3_): *δ* = 210.2 (major), 210.1 (minor), 179.7 (minor), 179.4 (major), 176.9 (major), 51.4 (minor), 50.4 (major), 42.0 (minor), 41.9 (major), 41.4 (major), 40.1 (minor), 32.7 (minor), 32.1 (major), 32.0 (major), 29.6 (minor), 27.3 (minor), 27.2 (major), 25.1 (major), 25.0 (minor), 25.0 (major), 24.9 (minor) ppm; HPLC: ChiralPak AD-H column, hexanes/*i*-PrOH (90/10), flow rate = 1.0 mL/min, λ = 210 nm, *t_R_* (*anti*) = 28.24 min (major enantiomer) 34.68 min (minor enantiomer), *t_R_* (*syn*) = 114.20 min (major enantiomer) 82.43 min (minor enantiomer).

*1-Ethyl-3-(2-oxocyclohexyl)pyrrolidine-2,5-dione* (**11ah**) [43]. The major *anti* diastereomer was obtained combined with the minor *syn* diastereomer as an inseparable mixture in 93% global yield (*anti*/*syn* = 83/17, ^1^H NMR on the reaction crude). White solid, mp: 96–98 °C; ^1^H NMR (400 MHz, CDCl_3_): *δ* = 3.65–3.48 (m, 2H), 3.22–3.06 (m, 1H), 3.03–2.60 (m, 2H), 2.50–2.40 (m, 1H), 2.39–2.24 (m, 2H), 2.20–2.05 (m. 2H), 2.01–1.91 (m, 1H), 1.83–1.49 (m, 3H), 1.25–1.13 (m, 3H) ppm; ^13^C NMR (101 MHz, CDCl_3_): *δ* = 210.2 (major), 210.1 (minor), 179.4 (minor), 179.2 (major), 176.7 (major), 51.6 (minor), 50.5 (major), 42.0 (minor), 41.9 (major), 41.3 (major), 39.9 (minor), 33.9 (major), 33.8 (minor), 32.8 (minor), 32.0 (major), 31.9 (major), 29.5 (minor), 27.3 (minor), 27.1 (major), 25.1 (major), 13.0 (minor), 12.7 (major) ppm; HPLC: ChiralPak AD-H column, hexanes/*i*-PrOH (90/10), flow rate = 1.0 mL/min, λ = 210 nm, *t_R_* (*anti*) = 19.81 min (major enantiomer) 27.62 min (minor enantiomer), *t_R_* (*syn*) = 32.04 min (major enantiomer) 78.12 min (minor enantiomer).

*3-(2-Oxocyclohexyl)pyrrolidine-2,5-dione* (**11ai**). The major *anti* diastereomer was obtained combined with the minor *syn* diastereomer as an inseparable mixture in 92% global yield (*anti*/*syn* = 76/24, ^1^H NMR on the reaction crude). White solid, mp: 159–161 °C; ^1^H NMR (400 MHz, CDCl_3_): *δ* = 8.63 (br s, 1H), 3.22–3.09 (m, 1H), 3.01–2.66 (m, 2H), 2.59–2.45 (m, 1H), 2.44–2.28 (m, 2H), 2.19–2.07 (m, 2H), 2.00–1.86 (m, 1H), 1.83–1.47 (m, 3H) ppm; ^13^C NMR (101 MHz, CDCl_3_): *δ* = 210.3 (major), 210.1 (minor), 180.1 (minor), 179.7 (major), 177.2 (minor), 177.0 (major), 51.3 (minor), 50.3 (major), 42.6 (major), 42.0 (minor), 41.8 (major), 41.4 (minor), 33.8 (minor), 33.2 (major), 32.0 (major), 29.5 (minor), 27.3 (minor), 27.2 (major), 25.0 (major) ppm; IR (neat): *υ* = 3244 (br), 3039 (br), 2943 (w), 2873 (w), 1766 (w), 1693 (vs), 1354 (w), 1180 (w), 833 (w), 791 (w), 737 (w) cm^−1^; MS (70 eV, EI): *m*/*z* (%): 55.1 (17), 97.1 (100), 99.1 (65), 195.1 (M^+^, 27); HRMS (EI-QTOF) calcd. for C_10_H_13_NO_3_ (*M*^+^): 195.0895. Found: 195.0888; HPLC: ChiralPak AD-H column, hexanes/*i*-PrOH (90/10), flow rate = 1.0 mL/min, λ = 254 nm, *t_R_* (*anti*) = 56.48 min, *t_R_* (*syn*) = 25.46 min.

*3-(2-Oxocyclopentyl)-1-phenylpyrrolidine-2,5-dione* (**11ba**) [50]. The major *anti* diastereomer was obtained combined with the minor *syn* diastereomer as an inseparable mixture in 89% global yield (*anti*/*syn* = 51/49, ^1^H NMR on the reaction crude). Brown solid, mp: 66–68 °C; ^1^H NMR (300 MHz, CDCl_3_): *δ* = 7.53–7.32 (m, 4H, major + minor), 7.31–7.20 (m, 1H, major + minor), 3.54–3.20 (m, 1H, major + minor), 3.09–2.79 (m, 2H, major + minor), 2.65–2.23 (m, 3H, major + minor), 2.22–2.06 (m, 2H, major + minor), 1.98–1.80 (m, 1H, major + minor), 1.76–1.59 (m, 1H, major + minor) ppm; ^13^C NMR (75 MHz, CDCl_3_): *δ* = 218.2 (minor), 217.7 (major), 178.2 (minor), 177.9 (major), 175.3 (major), 175.2 (minor), 132.1 (major), 131.9 (minor), 129.3 (major), 128.9 (minor), 128.8 (major), 126.7 (major), 126.6 (minor), 50.4 (major), 50.2 (minor), 39.6 (major), 38.8 (minor), 37.8 (minor), 37.7 (major), 32.5 (major), 32.4 (minor), 27.2 (major), 25.5 (minor), 20.8 (major), 20.5 (minor) ppm; HPLC: ChiralPak AD-H column, hexanes/*i*-PrOH (70/30), flow rate = 0.5 mL/min, λ = 230 nm, *t_R_* (*anti*) = 36.08 min (major enantiomer) 33.94 min (minor enantiomer), *t_R_* (*syn*) = 39.87 min (major enantiomer) 33.40 min (minor enantiomer).

*3-(3,3-Dimethyl-2-oxocyclopentyl)-1-phenylpyrrolidine-2,5-dione* (**11ca**). The major *anti* diastereomer was obtained combined with the minor *syn* diastereomer as an inseparable mixture in 65% global yield (*anti*/*syn* = 64/36, ^1^H NMR on the reaction crude). White solid, mp: 95–97 °C; ^1^H NMR (400 MHz, CDCl_3_): *δ* = 7.51–7.45 (m, 2H), 7.43–7.34 (m, 2H), 7.28–7.24 (m, 1H), 3.51–3.19 (m, 1H), 3.12–2.93 (m, 2H), 2.61–2.36 (m, 1H), 2.25–2.02 (m, 1H), 1.95–1.67 (m, 3H), 1.13 (d, *J* = 5.5 Hz, 3H), 1.02 (d, *J* = 9.3 Hz, 3H) ppm; ^13^C NMR (101 MHz, CDCl_3_): *δ* = 222.0 (minor), 221.4 (major), 178.2 (minor), 177.9 (major), 175.4 (major), 175.3 (minor), 132.2 (minor), 132.0 (major), 129.4 (major), 128.9 (minor), 128.8 (major), 126.8 (major), 126.7 (minor), 50.4 (major), 50.0 (minor), 44.9 (minor), 44.8 (major), 39.6 (major), 39.0 (minor), 36.6 (major), 36.3 (minor), 32.6 (major), 25.1 (minor), 25.0 (major), 23.9 (minor), 23.8 (major), 23.7 (minor), 21.9 (major) ppm; IR (neat): *υ* = 2970 (s), 2904 (s), 1782 (w), 1705 (vs), 1500 (w), 1458 (w), 1389 (s), 1242 (w), 1184 (s), 1061 (vs), 879 (w), 760 (w), 694 (w) cm^−1^; MS (70 eV, EI): *m*/*z* (%): 54.1 (21), 111.1 (28), 119.1 (41), 175.1 (100), 201.1 (28), 285.1 (M^+^, 28); HRMS (EI-QTOF) calcd. for C_17_H_19_NO_3_ (*M*^+^): 285.1365. Found: 285.1363; HPLC: ChiralPak AD-H column, hexanes/*i*-PrOH (90/10), flow rate = 0.7 mL/min, λ = 254 nm, *t_R_* (*anti*) = 60.81 min (major enantiomer) 44.23 min (minor enantiomer), *t_R_* (*syn*) = 65.94 min (major enantiomer) 55.30 min (minor enantiomer).

*3-(2-Oxocycloheptyl)-1-phenylpyrrolidine-2,5-dione* (**11da**) [47]. The major *anti* diastereomer was obtained combined with the minor *syn* diastereomer as an inseparable mixture in 92% global yield (*anti*/*syn* = 66/34, ^1^H NMR on the reaction crude). Grey solid, mp: 102–104 °C; ^1^H NMR (300 MHz, CDCl_3_): *δ* = 7.51–7.43 (m, 2H), 7.42–7.35 (m, 1H), 7.31–7.28 (m, 2H), 3.58–3.23 (m, 1H), 3.12–2.92 (m, 1H), 2.91–2.70 (m, 1H), 2.69–2.54 (m, 1H), 2.52–2.33 (m, 1H), 2.08–1.95 (m, 2H), 1.94–1.52 (m, 5H), 1.49–1.10 (m, 2H) ppm; ^13^C NMR (75 MHz, CDCl_3_): *δ* = 213.9 (major), 213.2 (minor), 178.6 (major), 176.0 (major), 175.9 (minor), 132.4 (major), 132.1 (minor), 129.2 (major), 128.7 (minor), 128.6 (major), 126.8 (major), 126.6 (minor), 52.8 (minor), 51.8 (major), 43.7 (major), 43.5 (minor), 43.4 (major), 42.3 (minor), 33.0 (minor), 31.9 (major), 30.1 (minor), 29.9 (major), 29.8 (major), 29.7 (minor), 29.4 (major), 28.7 (minor), 23.4 (major) ppm ; HPLC: ChiralPak AD-H column, hexanes/*i*-PrOH (70/30), flow rate = 0.8 mL/min, λ = 230 nm, *t_R_* (*anti*) = 30.73 min (major enantiomer) 67.64 min (minor enantiomer), *t_R_* (*syn*) = 28.47 min (major enantiomer) 25.08 min (minor enantiomer).

*3-(4-Oxo-1-*tert*-butoxycarbonylpiperidine-3-yl)-1-phenylpyrrolidin-2,5-dione* (**11ea**). The major *anti* diastereomer was obtained combined with the minor *syn* diastereomer as an inseparable mixture in 94% global yield (*anti*/*syn* = 56/44, ^1^H NMR on the reaction crude). Yellow solid, mp: 74–76 °C; ^1^H NMR (300 MHz, CDCl_3_): *δ* = 7.55–7.44 (m, 2H, major + minor), 7.43–7.37 (m, 1H, major + minor), 7.36–7.29 (m, 2H, major + minor), 4.66–4.20 (m, 2H, major + minor), 3.51–3.03 (m, 3H, major + minor), 2.98–2.81 (m, 2H, major + minor), 2.68–2.35 (m, 3H, major + minor), 1.51 (s, 9H, major + minor) ppm; ^13^C NMR (75 MHz, CDCl_3_): *δ* = 207.3 (minor), 207.0 (major), 178.1 (minor), 177.6 (major), 175.3 (major), 175.2 (minor), 154.8 (minor), 154.3 (major), 132.3 (major), 132.2 (minor), 129.3 (major), 128.8 (major), 126.8 (major), 81.2 (major), 81.0 (minor), 51.9 (minor), 50.1 (major), 41.3 (minor), 41.1 (major), 38.7 (major), 38.5 (minor), 31.9 (major), 28.5 (minor), 28.4 (major) ppm; IR (neat): *υ* = 2989 (w), 2935 (w), 1778 (w), 1701 (vs), 1500 (w), 1389 (s), 1242 (w), 1165 (s), 864 (w), 756 (w); 694 (w) cm^−1^; HRMS (ESI-QTOF) calcd. for C_20_H_24_N_2_O_5_Na [(*M*+Na)^+^]: 395.1583. Found: 395.1582; HPLC: ChiralPak IC column, hexanes/*i*-PrOH (70/30), flow rate = 1.0 mL/min, λ = 230 nm, *t_R_* (*anti*) = 166.10 min (major enantiomer) 106.81 min (minor enantiomer), *t_R_* (*syn*) = 178.57 min (major enantiomer) 42.19 min (minor enantiomer).

*3-(4-Oxo-1-propylpiperidin-3-yl)-1-phenylpyrrolidine-2,5-dione* (**11fa**). The major *anti* diastereomer was obtained combined with the minor *syn* diastereomer as an inseparable mixture in 91% global yield (*anti*/*syn* = 65/35, ^1^H NMR on the reaction crude). Red solid, mp: 68–70 °C; ^1^H NMR (300 MHz, CDCl_3_): *δ* = 7.52–7.44 (m, 2H), 7.42–7.38 (m, 1H), 7.37–7.29 (m, 2H), 3.56–3.24 (m, 1H), 3.23–2.94 (m, 3H), 2.92–2.79 (m, 1H), 2.78–2.56 (m, 2H), 2.55–2.16 (m, 5H), 1.63–1.47 (m, 2H), 1.00–0.90 (m, 3H) ppm; ^13^C NMR (75 MHz, CDCl_3_): *δ* = 208.5 (minor), 208.4 (major), 178.2 (minor), 178.0 (major), 175.6 (major), 175.5 (minor), 132.4 (major), 132.2 (minor), 129.3 (major), 128.7 (major), 126.8 (major), 126.7 (minor), 59.1 (major), 57.1 (major), 56.6 (minor), 53.3 (major), 53.2 (minor), 51.4 (minor), 50.1 (major), 41.1 (minor), 41.0 (major), 39.3 (major), 39.0 (minor), 34.4 (minor), 32.5 (major), 20.8 (major), 12.0 (minor), 11.9 (major) ppm; IR (neat): *υ* = 2981 (w), 2924 (w), 1782 (w), 1705 (vs), 1647 (w), 1500 (w), 1385 (s), 1184 (s), 756 (w), 694 (s) cm^−1^; MS (70 eV, EI): *m*/*z* (%): 110.1 (<10), 140.1 (100), 256.1 (<10), 285.2 (M^+^, 26); HRMS (EI-QTOF) calcd. for C_18_H_22_N_2_O_3_ (*M*^+^): 314.1630. Found: 314.1619; HPLC: ChiralPak AD-H column, hexanes/*i*-PrOH (70/30), flow rate = 0.8 mL/min, λ = 240 nm, *t_R_* (*anti*) = 16.20 min (major enantiomer) 26.53 min (minor enantiomer), *t_R_* (*syn*) = 17.44 min (major enantiomer) 13.43 min (minor enantiomer).

*3-(4-Oxotetrahydro-2*H*-pyran-3-yl)-1-phenylpyrrolidine-2,5-dione* (**11ga**) [46]. The major *anti* diastereomer was obtained combined with the minor *syn* diastereomer as an inseparable mixture in 93% global yield (*anti*/*syn* = 58/42, ^1^H NMR on the reaction crude). Yellow solid, mp: 109–111 °C; ^1^H NMR (300 MHz, CDCl_3_): *δ* = 7.55–7.44 (m, 2H, major + minor), 7.43–7.36 (m, 1H, major + minor), 7.35–7.28 (m, 2H, major + minor), 4.47–4.08 (m, 2H, major + minor), 3.79–3.60 (m, 1H, major + minor), 3.58–3.43 (m, 1H, major + minor), 3.18–2.80 (m, 2H, major + minor), 2.79–2.57 (m, 2H, major + minor), 2.56–2.28 (m, 2H, major + minor) ppm; ^13^C NMR (75 MHz, CDCl_3_): *δ* = 206.3 (minor), 205.9 (major), 178.0 (minor), 177.5 (major), 175.3 (major), 175.2 (minor), 132.2 (major), 132.1 (minor), 129.3 (major), 128.8 (minor), 126.8 (major), 70.9 (minor), 70.7 (major), 53.0 (minor), 51.5 (major), 42.6 (minor), 42.5 (major), 37.4 (major), 34.5 (minor), 32.2 (major); 29.8 (minor) ppm; HPLC: ChiralPak AD-H column, hexanes/*i*-PrOH (90/10), flow rate = 0.6 mL/min, λ = 230 nm, *t_R_* (*anti*) = 197.01 min, *t_R_* (*syn*) = 177.10 min.

*3-(2-Oxopropyl)-1-phenylpyrrolidine-2,5-dione* (**11ha**) [49]. The product was obtained in 71% yield. White solid, mp: 101–103 °C; ^1^H NMR (300 MHz, CDCl_3_): *δ* = 7.52–7.37 (m, 3H), 7.35–7.29 (m, 2H), 3.22–3.03 (m, 4H), 2.63–2.51 (m, 1H), 2.22 (s, 3H) ppm; ^13^C NMR (75 MHz, CDCl_3_): *δ* = 205.7, 178.7, 175.6, 132.2, 129.3, 128.8, 126.7, 43.7, 35.7, 34.8, 29.9 ppm; HPLC: ChiralCel OD-H column, hexanes/*i*-PrOH (80/20), flow rate = 0.8 mL/min, λ = 230 nm, *t_R_* (*S*) = 45.40 min, *t_R_* (*R*) = 50.79 min.

*3-(2-Oxo-1-phenylpropyl)-1-phenylpyrrolidine-2,5-dione* (**11ia**). The major *anti* diastereomer was obtained combined with the minor *syn* diastereomer as an inseparable mixture in 56% global yield (*anti*/*syn* = 52/48, ^1^H NMR on the reaction crude). Colorless oil; ^1^H NMR (300 MHz, CDCl_3_): *δ* = 7.52–7.29 (m, 14H, major + minor), 7.25–7.15 (m, 4H, major + minor), 7.08–6.99 (m, 2H, major + minor), 4.60 (s, *J* = 4.3 Hz, 1H, major), 4.48 (d, *J* = 4.9 Hz, 1H, minor), 3.77 (dt, *J* = 9.4, 5.1, Hz, 1H, major), 3.16 (ddd, *J* = 9.6, 5.9, 4.4 Hz, 1H, minor), 3.09–2.96 (m, 2H, major), 2.70–2.53 (m, 2H, minor), 2.20 (s, 3H, major), 2.11 (s, 3H, minor) ppm; ^13^C NMR (75 MHz, CDCl_3_): *δ* = 207.4 (minor), 206.3 (major), 178.1 (major), 175.7 (minor), 175.3 (major), 136.0 (major), 133.5 (minor), 132.3 (minor), 131.8 (major), 129.8 (major), 129.6 (minor), 129.5 (major), 129.4 (minor), 129.3 (major), 128.8 (major), 128.7 (major), 128.5 (minor), 126.7 (major), 126.5 (minor), 58.8 (minor), 58.3 (major), 43.8 (major), 41.9 (minor), 32.1 (major), 32.0 (minor), 29.2 (major), 29.1 (minor) ppm; IR (neat): *υ* = 2962 (w), 2908 (w), 1778 (w), 1705 (vs), 1500 (w), 1385 (w), 1257 (w), 1176 (w), 1080 (s), 1022 (vs), 795 (vs), 694 (s) cm^−1^; MS (70 eV, EI): *m*/*z* (%): 91.1 (14), 115.1 (19), 117.1 (24), 174.1 (16), 265.1 (100), 266.1 (19), 307.1 (M^+^, <10) ; HRMS (EI-QTOF) calcd. for C_19_H_17_NO_3_ (*M*^+^): 307.1208. Found: 307.1210; HPLC: ChiralPak AD-H column, hexanes/*i*-PrOH (90/10), flow rate = 1.0 mL/min, λ = 280 nm, *t_R_* (*anti*) = 42.99 min (major enantiomer) 55.30 min (minor enantiomer), *t_R_* (*syn*) = 46.98 min (major enantiomer) 38.04 min (minor enantiomer).

*3-(1-Methoxy-2-oxopropyl)-1-phenylpyrrolidine-2,5-dione* (**11ja**) [50]. The major *anti* diastereomer was obtained combined with the minor *syn* diastereomer as an inseparable mixture in 94% global yield (*anti*/*syn* = 62/38, ^1^H NMR on the reaction crude). Yellow solid, mp: 67–69 °C; ^1^H NMR (300 MHz, CDCl_3_): *δ* = 7.53–7.43 (m, 3H), 7.33–7.25 (m, 2H), 4.39 (d, *J* = 2.3 Hz, 1H), 3.51 (s, 3H), 3.50–3.44 (m, 1H), 2.84–2.77 (m, 2H), 2.29 (s, 3H) ppm; ^13^C NMR (75 MHz, CDCl_3_): *δ* = 207.8 (major), 176.9 (major), 175.8 (major), 132.0 (major), 129.4 (major), 129.3 (minor), 128.9 (major), 128.8 (minor), 126.7 (minor), 126.5 (major), 86.3 (minor), 84.5 (major), 60.3 (major), 60.0 (minor), 42.6 (major), 42.2 (minor), 31.8 (minor), 29.2 (major), 27.2 (minor), 27.1 (major) ppm; HPLC: ChiralPak IB column, hexanes/*i*-PrOH (80/20), flow rate = 0.8 mL/min, λ = 240 nm, *t_R_* (*anti*) = 23.87 min (major enantiomer) 22.10 min (minor enantiomer), *t_R_* (*syn*) = 24.79 min (major enantiomer) 26.65 min (minor enantiomer).

*3-(1-Benzyloxy-2-oxopropyl)-1-phenylpyrrolidine-2,5-dione* (**11ka**). The major *anti* diastereomer was obtained combined with the minor *syn* diastereomer as an inseparable mixture in 96% global yield (*anti*/*syn* = 75/25, ^1^H NMR on the reaction crude). Yellow solid, mp: 85–87 °C; ^1^H NMR (300 MHz, CDCl_3_): *δ* = 7.44–7.30 (m, 9H), 7.21–7.18 (m, 1H), 4.84–4.53 (m, 3H), 3.56–3.48 (m, 1H), 2.88–2.78 (, 2H), 2.25 (s, 3H) ppm; ^13^C NMR (75 MHz, CDCl_3_): *δ* = 208.9 (minor), 207.6 (major), 177.0 (major), 175.8 (minor), 175.4 (minor), 175.2 (major), 136.7 (major), 136.4 (minor), 132.0 (minor), 131.9 (major), 129.3 (major), 129.3 (minor), 128.9 (major), 128.8 (major), 128.8 (minor), 128.7 (major), 128.5 (minor), 128.3 (minor), 128.2 (major), 126.7 (minor), 126.5 (major), 83.6 (minor), 82.4 (major), 74.6 (major), 73.9 (minor), 42.7 (major), 42.4 (minor), 31.8 (minor), 29.4 (major), 27.4 (minor), 27.3 (major) ppm; IR (neat): *υ* = 2939 (w), 2870 (w); 1786 (w), 1705 (vs), 1647 (w), 1597 (w), 1539 (w), 1496 (w), 1454 (w), 1389 (s), 1308 (w), 1192 (s), 1122 (w), 1049 (w), 945 (w), 744 (s), 694 (s) cm^−1^; HRMS (ESI-QTOF) calcd. for C_20_H_20_NO_4_ [(*M*+H)^+^]: 338.1392. Found: 338.1393; HPLC: ChiralCel OD-H column, hexanes/*i*-PrOH (80/20), flow rate = 1.0 mL/min, λ = 230 nm, *t_R_* (*anti*) = 31.89 min (major enantiomer) 26.74 min (minor enantiomer), *t_R_* (*syn*) = 95.87 min (major enantiomer) 49.79 min (minor enantiomer).

*3-(2-Oxocycloheptyl)pyrrolidine-2,5-dione* (**11di**): The major *anti* diastereomer was obtained combined with the minor *syn* diastereomer as an inseparable mixture in 85% global yield (*anti*/*syn* = 60/40, ^1^H NMR on the reaction crude). Yellow solid, mp: 71–73 °C; ^1^H NMR (500 MHz, CDCl_3_): *δ* = 8.27 (br s, 1H), 3.30–3.11 (m, 1H), 2.91–2.79 (m, 1H), 2.71–2.47 (m, 2H), 2.45–2.28 (m, 1H), 1.97–1.73 (m, 3H), 1.71–1.19 (m, 6H) ppm; ^13^C NMR (125 MHz, CDCl_3_): *δ* = 213.7 (major), 213.1 (minor), 179.7 (minor), 179.5 (major), 176.9 (minor), 176.7 (major), 52.0 (minor), 51.2 (major), 44.7 (major), 43.8 (major), 43.6 (minor), 43.5 (minor), 33.5 (minor), 33.2 (major), 32.1 (minor), 30.1 (minor), 29.8 (major), 29.7 (major), 29.4 (major), 28.1 (minor), 23.5 (major), 22.8 (minor) ppm; IR (neat): *υ* = 3120 (br), 2927 (w), 2858 (w), 1774 (w), 1701 (vs), 1639 (w), 1593 (w), 1539 (w), 1496 (w), 1450 (w), 1350 (s), 1308 (w), 1180 (vs), 1068 (s), 1018 (s), 945 (w), 910 (w), 812 (w), 756 (s), 725 (s) cm^−1^; MS (70 eV, EI): *m/z* (%): 54.1 (24), 55.1 (50), 67.1 (23), 83.1 (20), 98.1 (49), 99.1 (80), 111.1 (100), 112.1 (44), 151.1 (14), 209.1 (M^+^, 12); HRMS (EI-QTOF) calcd. for C_11_H_15_NO_3_ (*M*^+^): 209.1052. Found: 209.1046; HPLC: ChiralPak IB column, hexanes/EtOH (90/10), flow rate = 0.8 mL/min, λ = 240 nm, *t_R_* (*anti*) = 28.57 min (minor enantiomer) 32.76 min (major enantiomer), *t_R_* (*syn*) = 26.67 min (minor enantiomer) 30.05 min (major enantiomer).

*3-(4-Oxotetrahydro-2*H*-pyran-3-yl)pyrrolidine-2,5-dione* (**11gi**): The major *anti* diastereomer was obtained combined with the minor *syn* diastereomer as an inseparable mixture in 90% global yield (*anti*/*syn* = 51/49, ^1^H NMR on the reaction crude). Yellow solid, mp: 52–54 °C; ^1^H NMR (500 MHz, CDCl_3_): *δ* = 4.31–4.10 (m, 1H, major + minor), 4.02–3.38 (m, 2H, major + minor), 3.37–2.80 (m, 1H, major + minor), 2.79–2.30 (m, 2H, major + minor), 1.91–1.20 (m, 4H, major + minor) ppm; ^13^C NMR (125 MHz, CDCl_3_): *δ* = 210.3 (major), 205.9 (major), 205.6 (minor), 178.0 (major), 175.6 (minor), 70.5 (minor), 69.3 (major), 68.8 (major). 68.5 (minor), 63.3 (major), 60.0 (minor), 52.3 (major), 51.2 (minor), 44.2 (major), 42.6 (minor), 38.8 (minor), 36.8 (major), 35.2 (minor), 34.8 (major), 33.4 (minor), 29.9 (major) ppm; IR (neat): *υ* = 3232 (br), 2931 (w), 2866 (w), 1774 (w), 1705 (vs), 1639 (w), 1593 (w), 1543 (w), 1493 (w), 1454 (w), 1362 (w), 1308 (w), 1184 (s), 1149 (w), 1088 (w), 964 (w), 849 (w), 756 (w), 694 (w) cm^−1^; MS (70 eV, EI): *m/z* (%): 54.1 (70), 68.1 (19), 82.1 (<10), 99.1 (100), 197.0 (M^+^, <10); HRMS (EI-QTOF) calcd. for C_9_H_11_NO_4_ (*M*^+^): 197.0688. Found: 197.0690; HPLC: ChiralPak IB column, hexanes/EtOH (90/10), flow rate = 0.8 mL/min, λ = 240 nm, *t_R_* (*anti*) = 28.36 min, *t_R_* (*syn*) = 31.05 min.

### 3.3. Scaled-Up Enantioselective Michael Addition Reaction of Cyclohexanone and N-Phenylmaleimide

A mixture of organocatalyst **8** (172.7 mg, 0.74 mmol), DABCO (41.3 mg, 0.37 mmol), and **10a** (0.64 g, 3.7 mmol) was dissolved in CH_2_Cl_2_ (10 mL) in a glass vial (16 mm diameter). Then, **9a** (0.76 mL, 7,4 mmol) was added and the mixture was stirred for four days at 5 °C. After this time, the solvent was evaporated under reduced pressure (15 torr) and the crude reaction mixture was purified by column chromatography (hexanes/ethyl acetate gradients), yielding the corresponding diastereomeric adducts (0.92 g, 92% yield).

## 4. Conclusions

We have demonstrated that a simple primary amine-salicylamide derived from chiral *trans*-cyclohexane-1,2-diamine acts as an appropriate organocatalyst for the diastereo- and enantio-selective conjugate addition of ketones to maleimides, with the presence of an organic base such as 1,4-diazabicyclo[2.2.2]octane (DABCO) generally improving the stereoselectivity. Cyclic and acyclic ketones are used in this addition reaction with *N*-aryl- and *N*-alkyl-maleimides, usually affording the final succinimides in good yields. The stereoselectivity of the process ranges from low to very good in the case of diastereoselectivity and from moderate to excellent concerning enantioselectivity, even when using simple *N*-unsubstituted maleimide. This asymmetric procedure is an interesting alternative to the short array of methodologies leading to these substituted ketone-containing succinimides.

## Figures and Tables

**Table 1:** Organocatalytic conjugate addition of cyclohexanone to *N*-phenylmaleimide. Optimization of the reaction conditions.

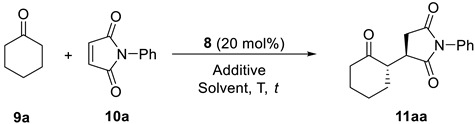							
Entry	Additive (mol%) ^a^	Solvent ^a^	T (°C)	*t* (d)	Conv. (%) ^b^	*dr* ^c^	*ee* (%) ^d^
1	-	Toluene	25	3	93	76/24	94 (55)
2	-	Xylene	25	3	76	79/21	89 (49)
3	-	Hexane	25	3	91	63/37	31 (8)
4	-	CH_2_Cl_2_	25	3	100	80/20	99 (40)
5	-	CHCl_3_	25	3	100	80/20	92 (50)
6	-	DCE	25	3	100	78/22	97 (30)
7	-	Et_2_O	25	3	55	72/28	78 (25)
8	-	THF	25	3	63	67/33	38 (32)
9	-	MeCN	25	3	69	71/29	41 (5)
10	-	MeOH	25	3	n.r.	n.d.	n.d.
11	-	*i*PrOH	25	3	49	71/29	50 (11)
12	-	H_2_O	25	3	100	67/33	95 (6)
13	-	CH_2_Cl_2_	25	5	100	80/20	99 (41)
14	PhCO_2_H (10)	CH_2_Cl_2_	25	3	100	77/23	51 (40)
15	4-O_2_NC_6_H_4_CO_2_H (10)	CH_2_Cl_2_	25	3	100	77/23	47 (40)
16	Salicylic acid (10)	CH_2_Cl_2_	25	3	100	74/26	33 (30)
17	HDA (10)	CH_2_Cl_2_	25	3	100	76/24	83 (58)
18	DMAP (10)	CH_2_Cl_2_	25	3	100	69/31	87 (22)
19	Imidazole (10)	CH_2_Cl_2_	25	3	100	83/17	83 (74)
20	DBU (10)	CH_2_Cl_2_	25	3	n.r.	n.d.	n.d
21	2,6-Lutidine (10)	CH_2_Cl_2_	25	3	100	81/19	84 (6)
22	DABCO (10)	CH_2_Cl_2_	25	3	100	85/15	99 (9)
23	DABCO (5)	CH_2_Cl_2_	25	3	100	85/15	97 (9)
24	DABCO (20)	CH_2_Cl_2_	25	3	100	85/15	64 (16)
25	DABCO (10)	CH_2_Cl_2_	5	4	100	93/7	99 (19)

^a^ Abbreviations: DABCO: 1,4-Diazabicyclo[2.2.2]octane; DBU: 1,8-Diazabicyclo[5.4.0]undec-7-ene; DCE: 1,2-Dichloroethane; DMAP: 4-(Dimethylamino)pyridine; HDA: Hexanedioic acid. ^b^ Determined by ^1^H NMR based on the remaining **10a**. ^c^ Diastereomeric ratio determined by ^1^H NMR on the reaction crude. ^d^ Determined by chiral HPLC on the reaction crude (see Section 3). Enantiomeric excess of the minor *anti* diastereomer in parentheses.

**Table 2:** Conjugate addition of ketones to maleimides organocatalyzed by **8**.

Entry	*t* (d)	Product 11	Yield (%) ^a^	*dr* ^b^	*ee* (%) ^c,d^
1	4	**11aa**	98	93/7	99 (19)
2	4	**11ab**	91	90/10	76 (53)
3	4	**11ac**	90	85/15	64 (53)
4	4	**11ad**	82	88/12	78 (46)
5	4	**11ae**	95	92/8	73 (18)
6	4	**11af**	80	84/16	62 (40)
7	4	**11ag**	94	91/9	76 (25)
8	4	**11ah**	93	83/17	77 (63)
9	4	**11ai**	92	76/24	99 (99)
10 ^e,f^	5	**11ba**	89	51/49	62 (22)
11 ^e,f^	5	**11ca**	65	64/36	93 (81)
12 ^e,f^	5	**11da**	92	66/34	85 (21)
13 ^f^	4	**11ea**	94	56/44	62 (54)
14 ^f^	4	**11fa**	91	65/35	76 (76)
15 ^f^	4	**11ga**	93	58/42	99 (99)
16	4	**11ha**	71	-	72
17	5	**11ia**	56	52/48	96 (26)
18	4	**11ja**	94	62/38	36 (15)
19	4	**11ka**	96	75/25	75 (25)
20 ^e,f^	4	**11di**	85	60/40	95 (75)
21 ^f^	4	**11gi**	90	51/49	99 (99)
22 ^g^	4	**11aa**	92	91/9	97 (19)

^a^ Combined isolated yield of both diastereomers after flash chromatography. ^b^ Diastereomeric *anti*/*syn* ratio determined by ^1^H NMR on the reaction crude. ^c^ Determined by chiral HPLC on the reaction crude (see Materials and Methods). Enantiomeric excess of the minor diastereomer in parentheses. ^d^ The absolute stereochemistry was determined by comparing the elution order in chiral HPLC with the reported in literature, whereas the stereochemistry of unknown compounds was assigned by analogy (see Materials and Methods). ^e^ 4-O_2_NC_6_H_4_CO_2_H (10 mol%) was used as co-catalyst. ^f^ Reaction was carried out at 25 °C. ^g^ Scaled-up reaction (see Materials and Methods).

## Data Availability

The data presented in this study are available in Supplementary Material.

## Supplementary Materials

The following supporting information can be downloaded at: https://www.mdpi.com/article/10.3390/molecules27196668/s1, Figures S1–S42 NMR spectra of compounds **11**, Figures S43–S84: HPLC chromatograms of compounds **11**.

## Sample Availability

Samples of all compounds **11**, except compound **11ia**, are available from the authors.
