# Supplementary material for: Asymmetric Conjugate Addition of Ketones to Maleimides Organocatalyzed by a Chiral Primary Amine-Salicylamide
Source: Molecules. 2022 Oct 7;27(19):6668. doi: 10.3390/molecules27196668 (PMC9573004; doi:10.3390/molecules27196668)
Supplement: Supplementary file 1 [file molecules-27-06668-s001.zip › molecules-1910637-supplementary.pdf]

Supplementary materials

Asymmetric conjugate addition of ketones to maleimides organocatalyzed by a chiral primary amine-salicylamide

Alejandro Torregrosa-Chinillach, Rafael Chinchilla*

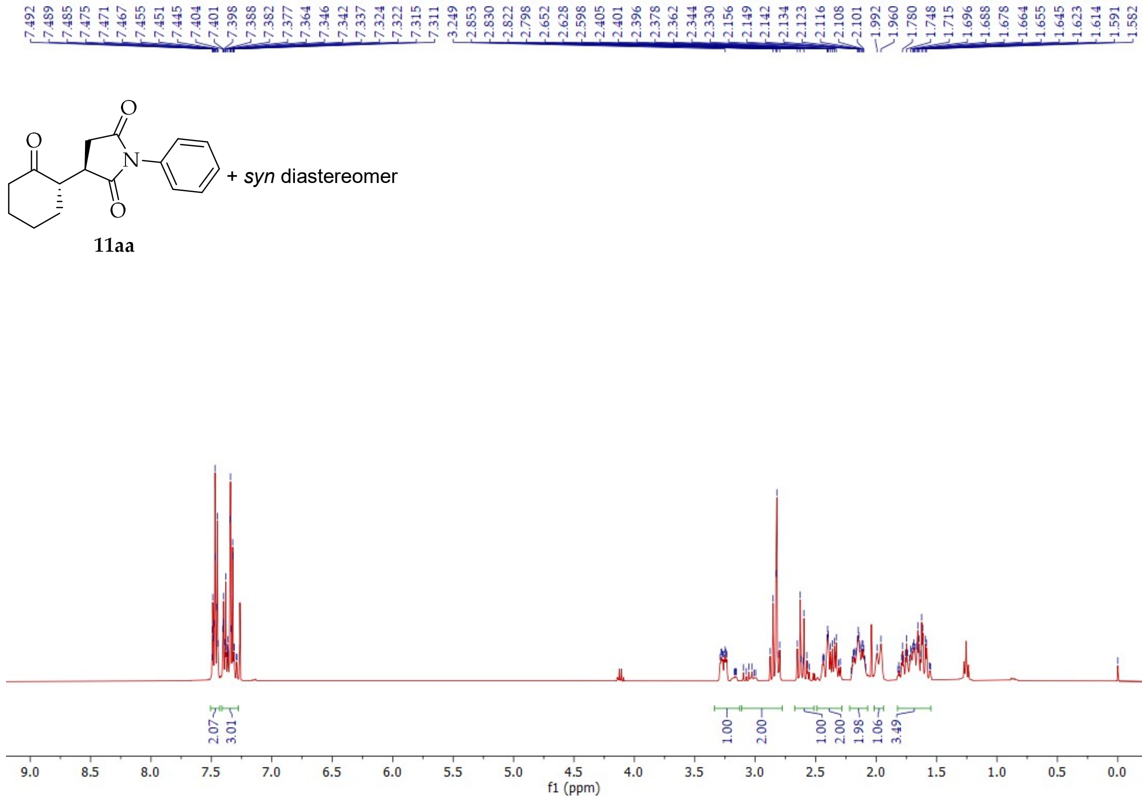

Figure S1. ^{1}H NMR spectrum (400 MHz) of compound **11aa**.

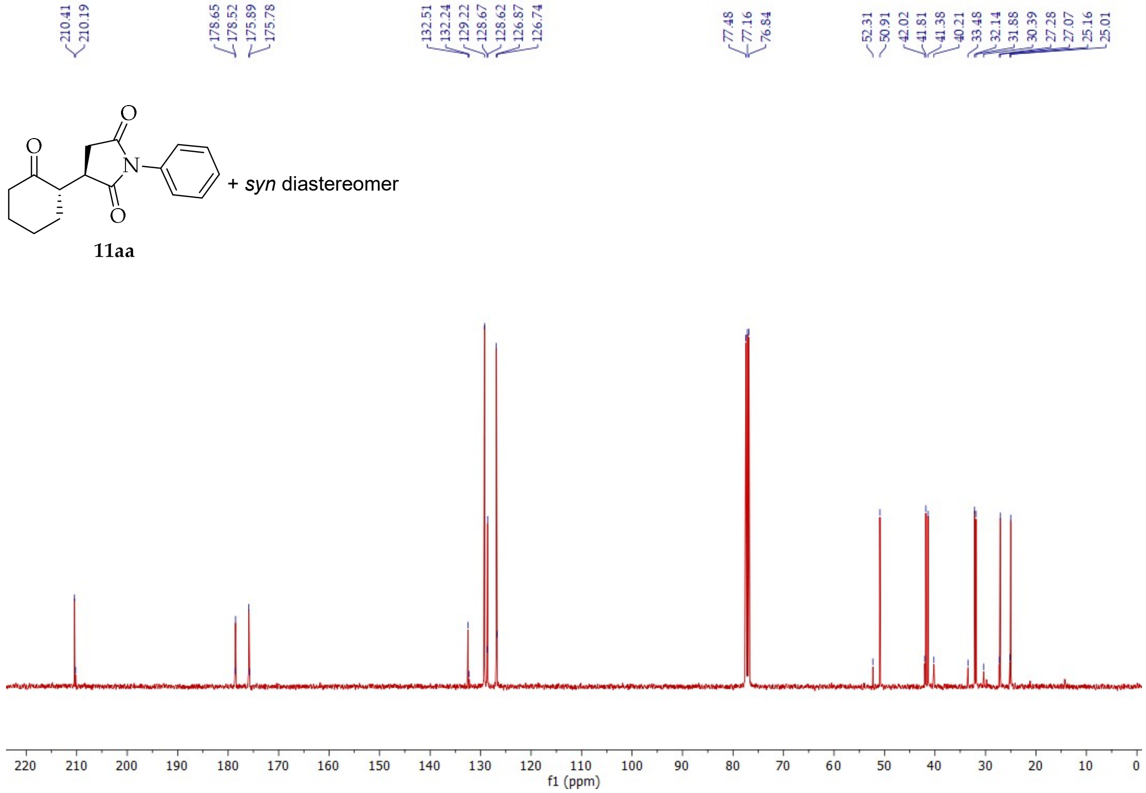

Figure S2. ^{13}C NMR spectrum (101 MHz) of compound **11aa**.

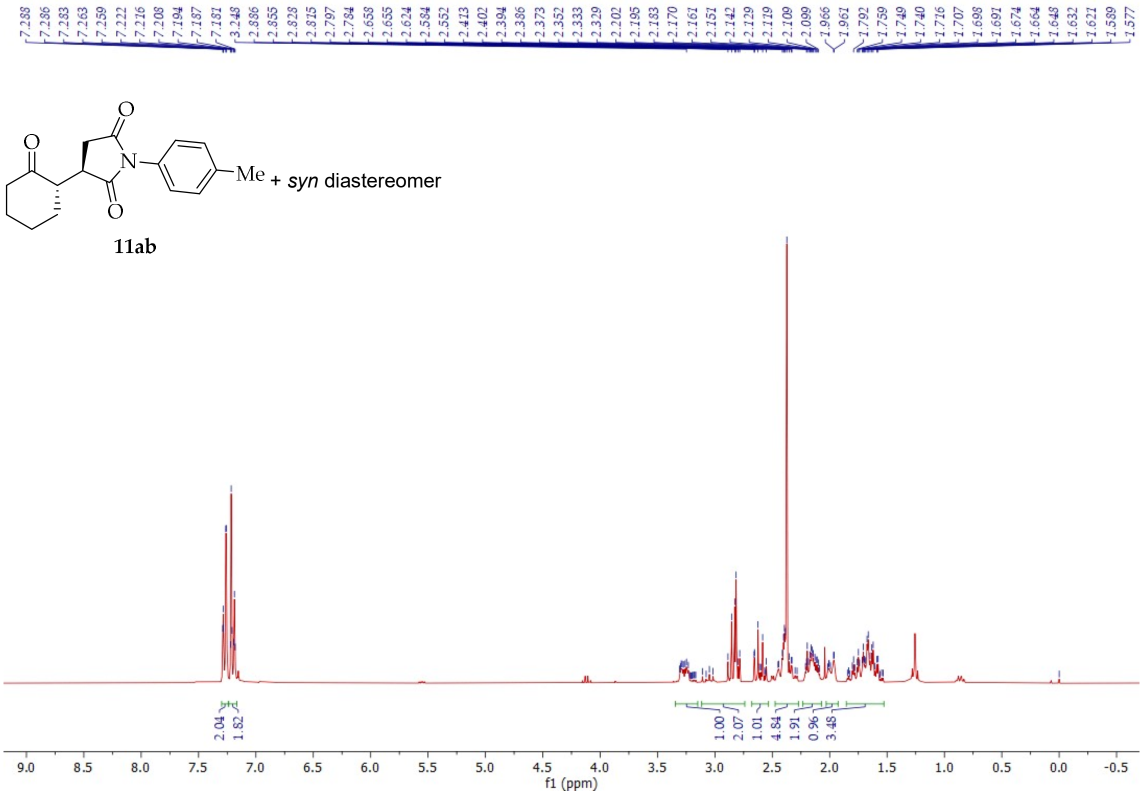

Figure S3. ^{1}H NMR spectrum (300 MHz) of compound **11ab**.

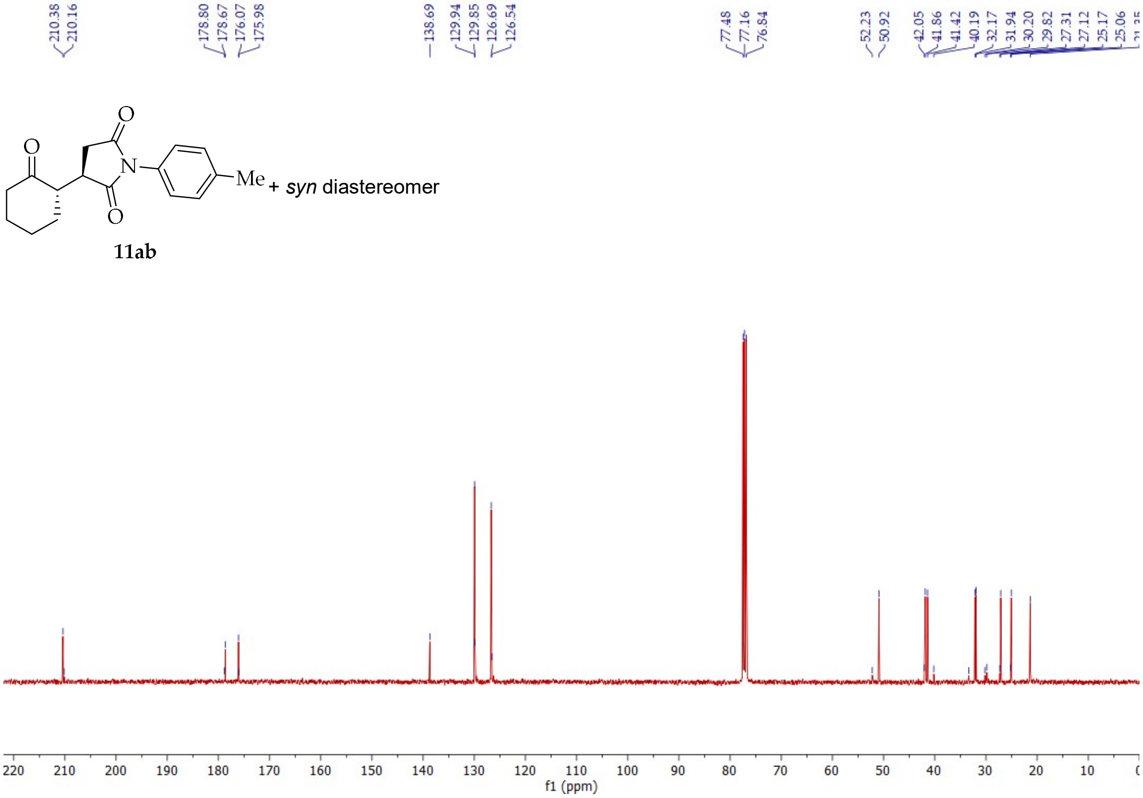

Figure S4. ^{13}C NMR spectrum (75 MHz) of compound **11ab**.

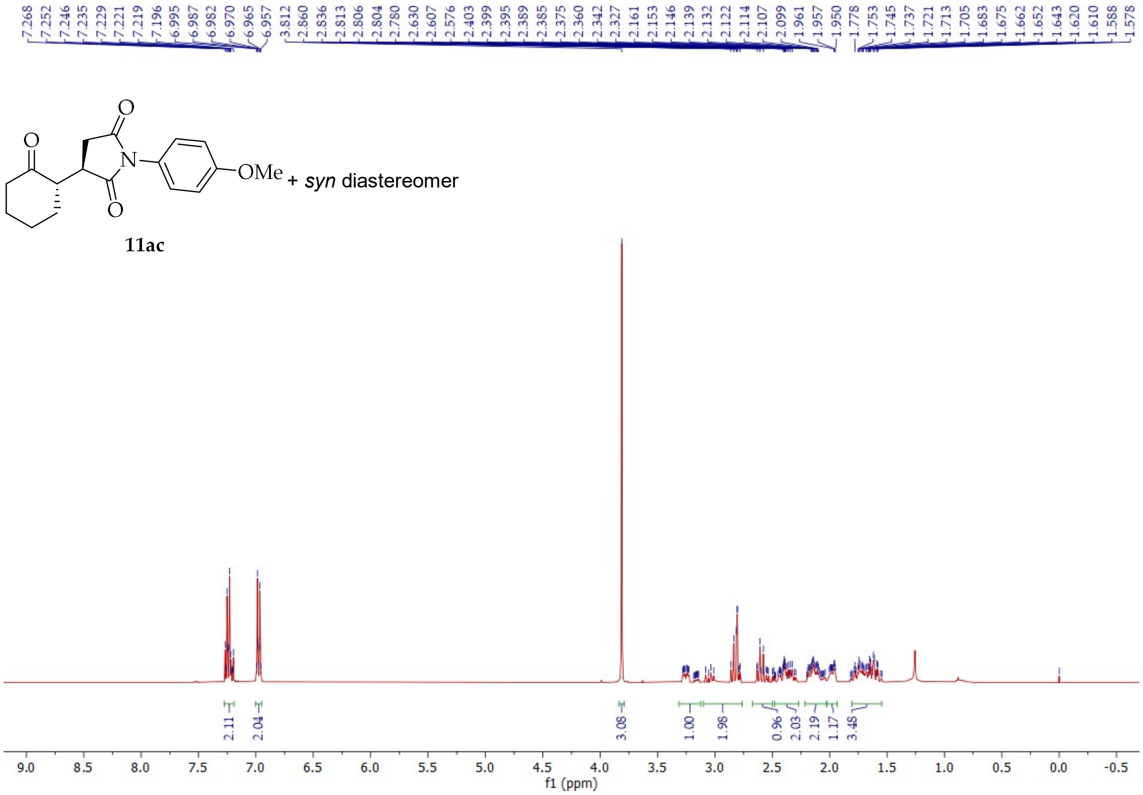

Figure S5. ^{1}H NMR spectrum (400 MHz) of compound **11ac**.

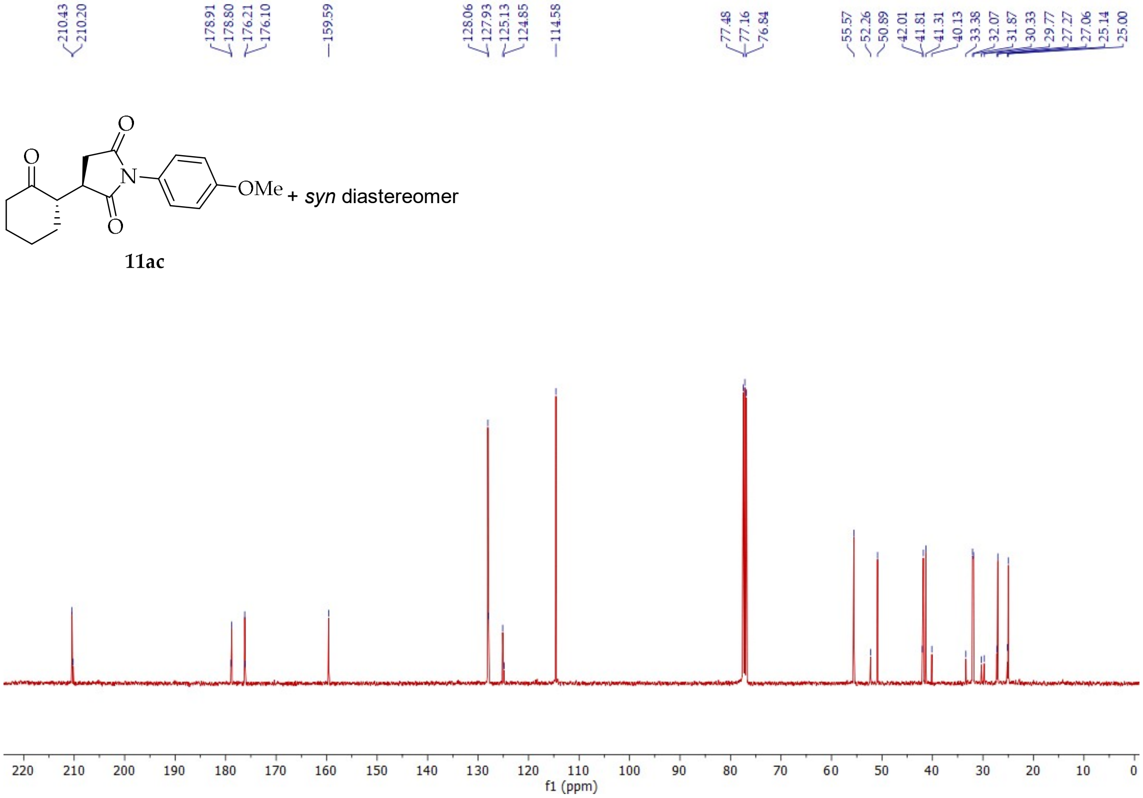

Figure S6. ^{13}C NMR spectrum (101 MHz) of compound **11ac**.

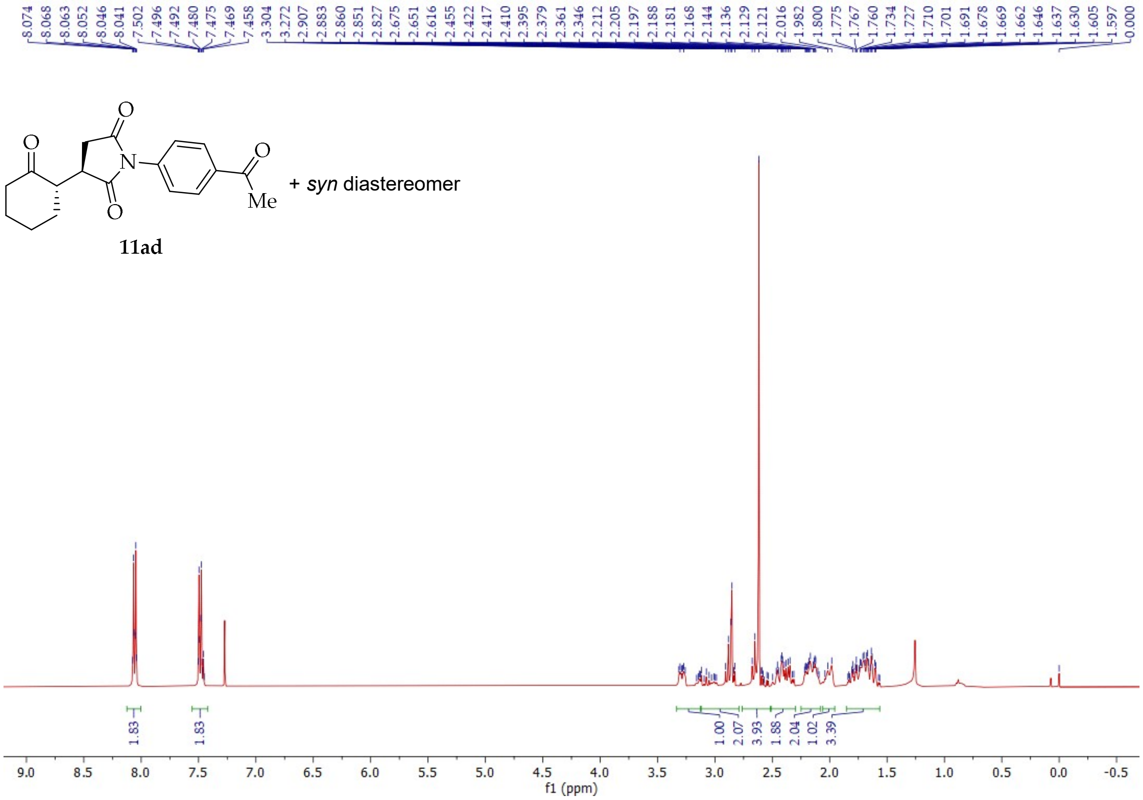

Figure S7. ^{1}H NMR spectrum (400 MHz) of compound **11ad**.

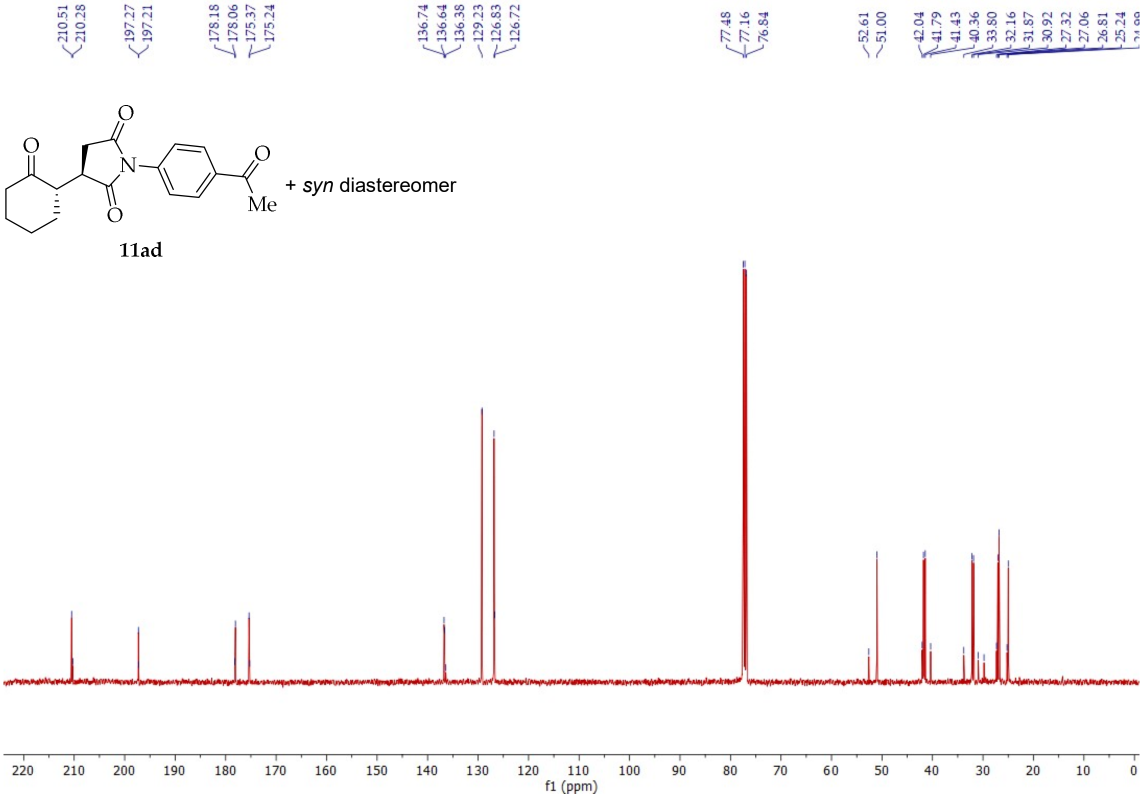

Figure S8. ^{13}C NMR spectrum (101 MHz) of compound **11ad**.

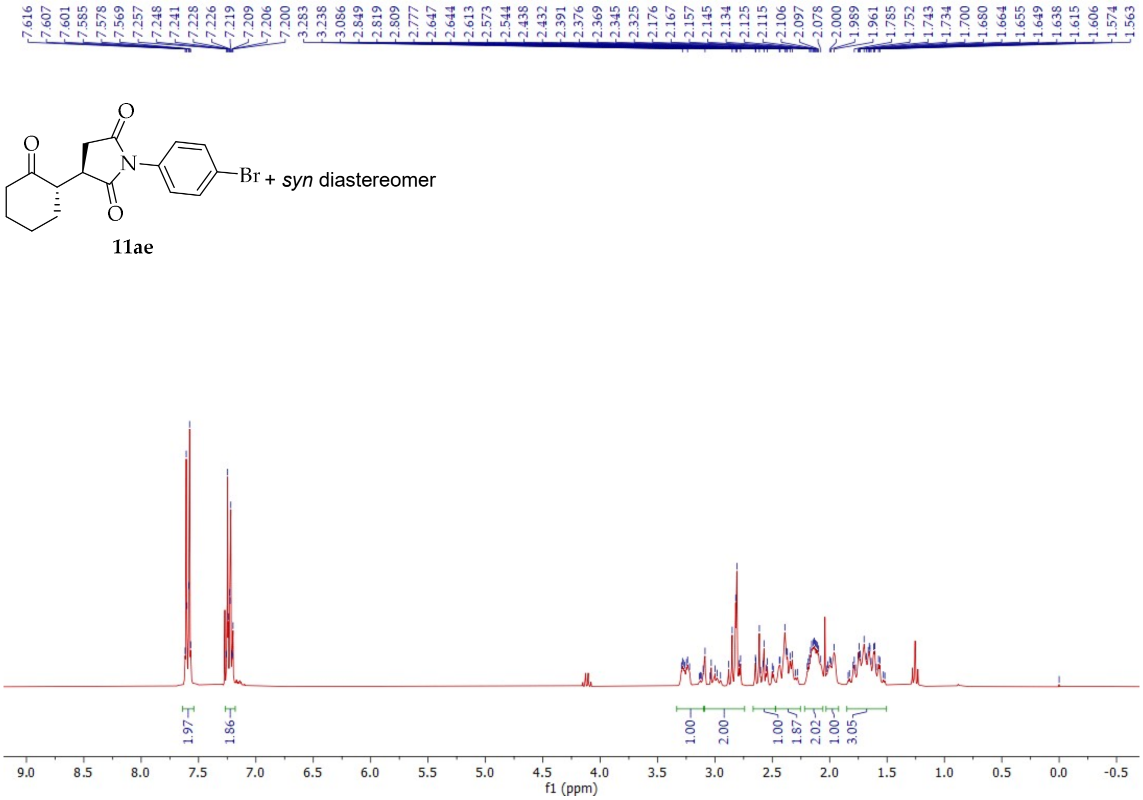

Figure S9. ^{1}H NMR spectrum (300 MHz) of compound **11ae**.

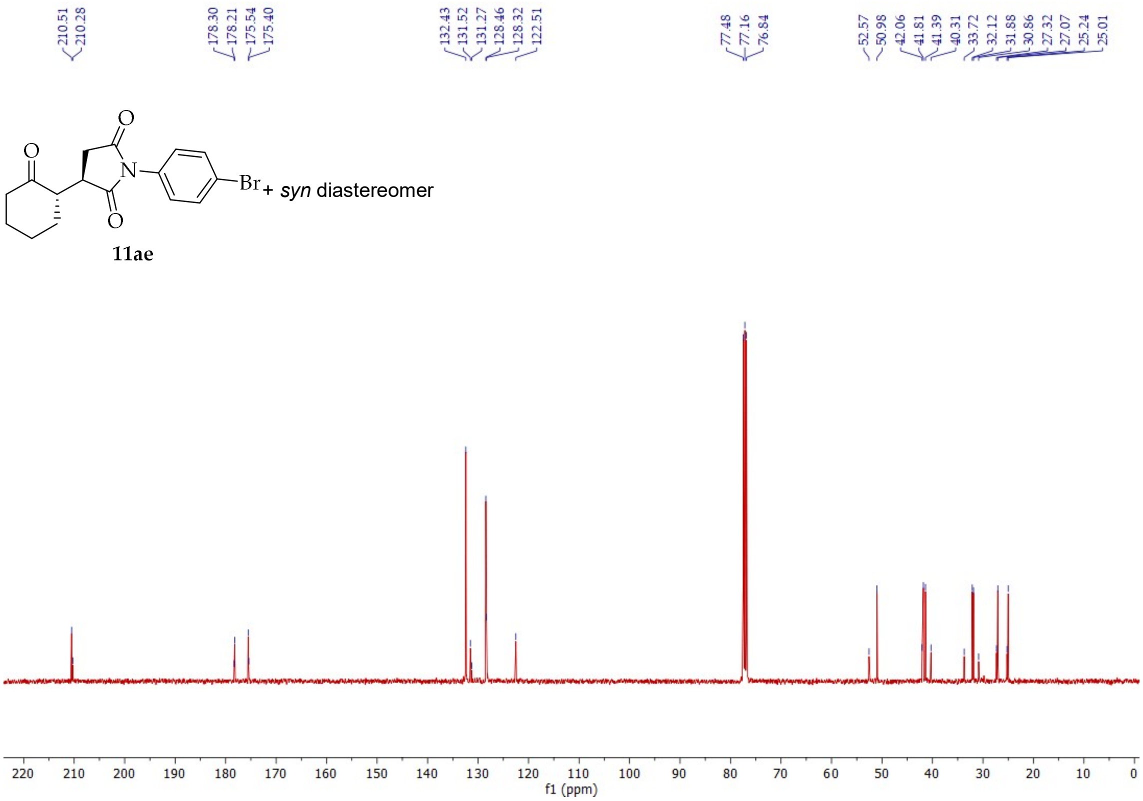

Figure S10. ^{13}C NMR spectrum (75 MHz) of compound **11ae**.

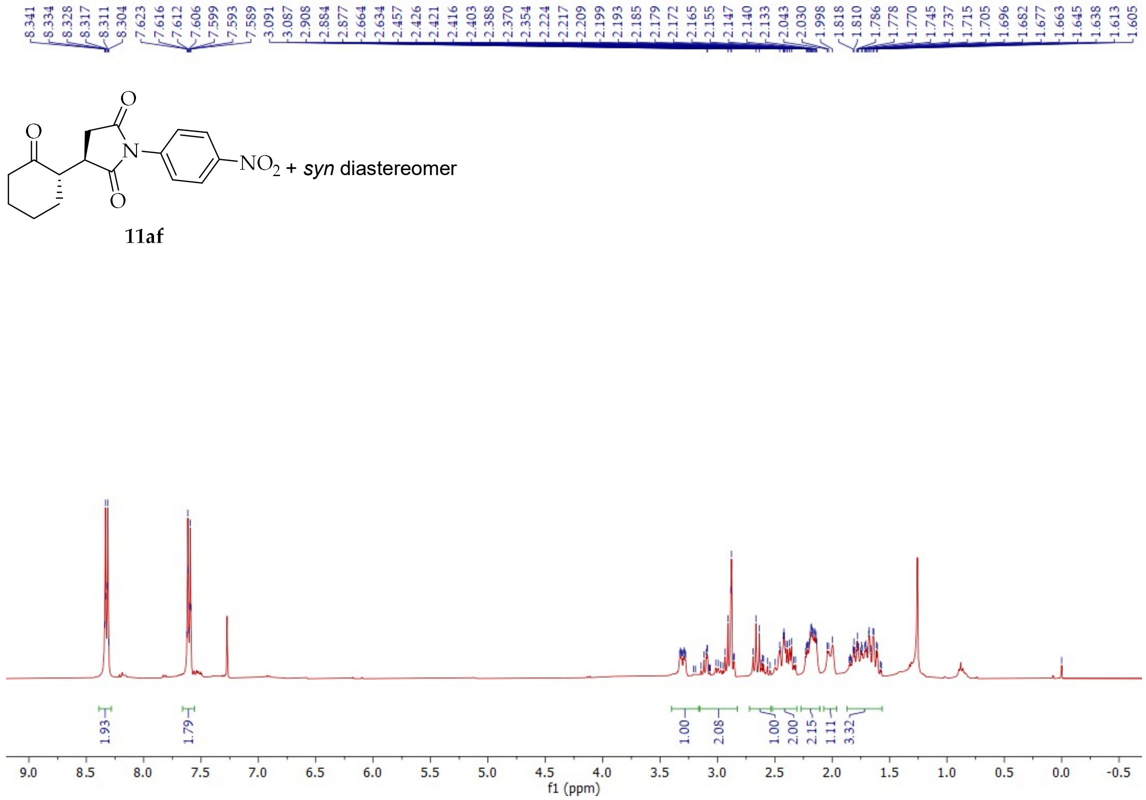

Figure S11. ^{1}H NMR spectrum (400 MHz) of compound **11af**.

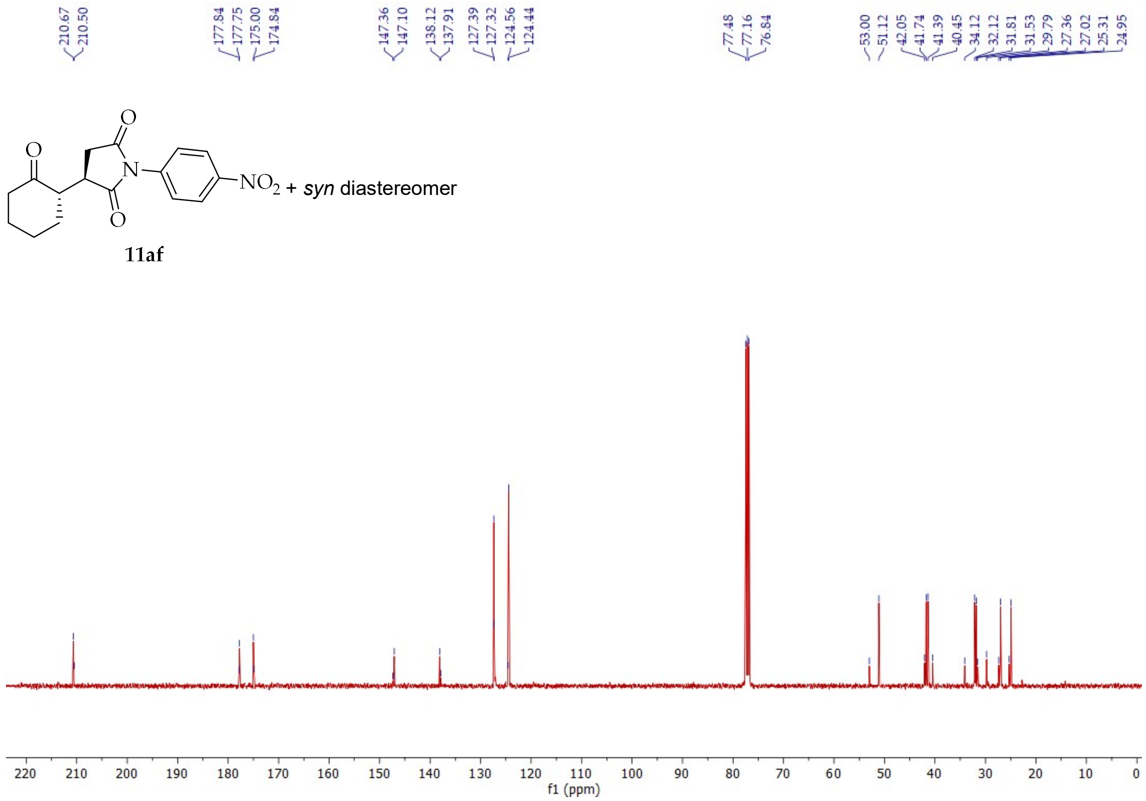

Figure S12. ^{13}C NMR spectrum (101 MHz) of compound **11af**.

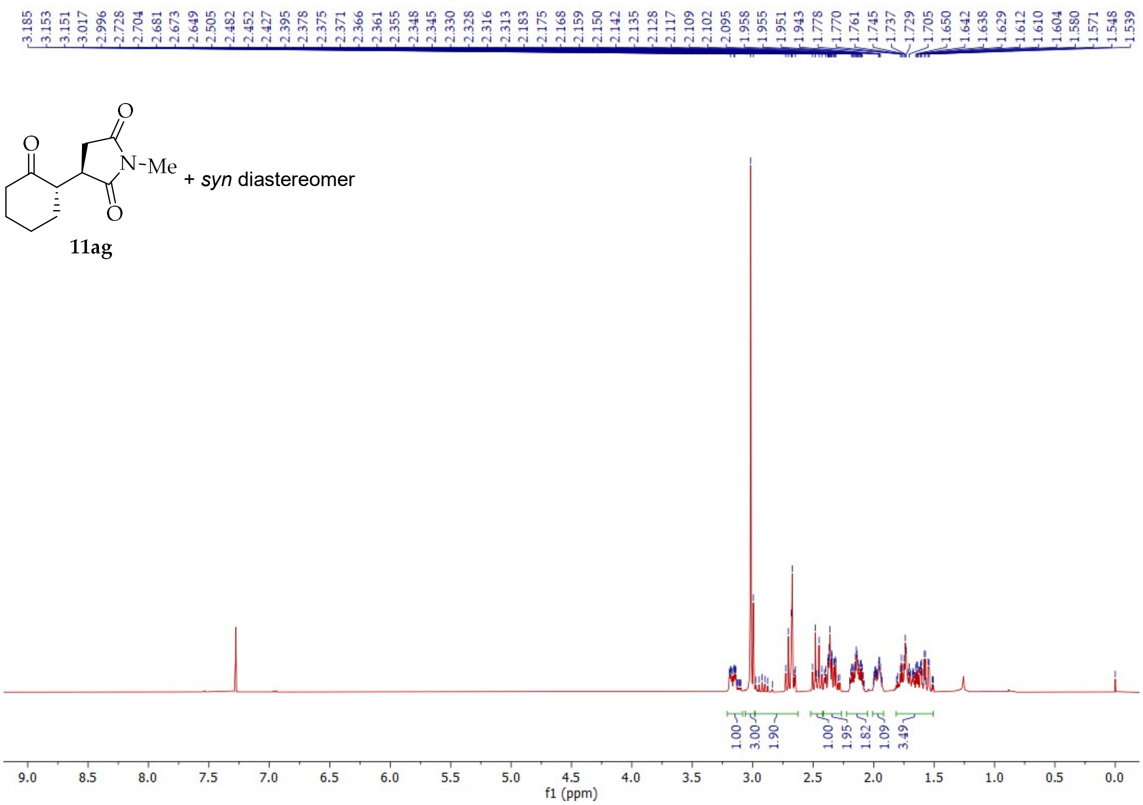

Figure S13. ^{1}H NMR spectrum (400 MHz) of compound **11ag**.

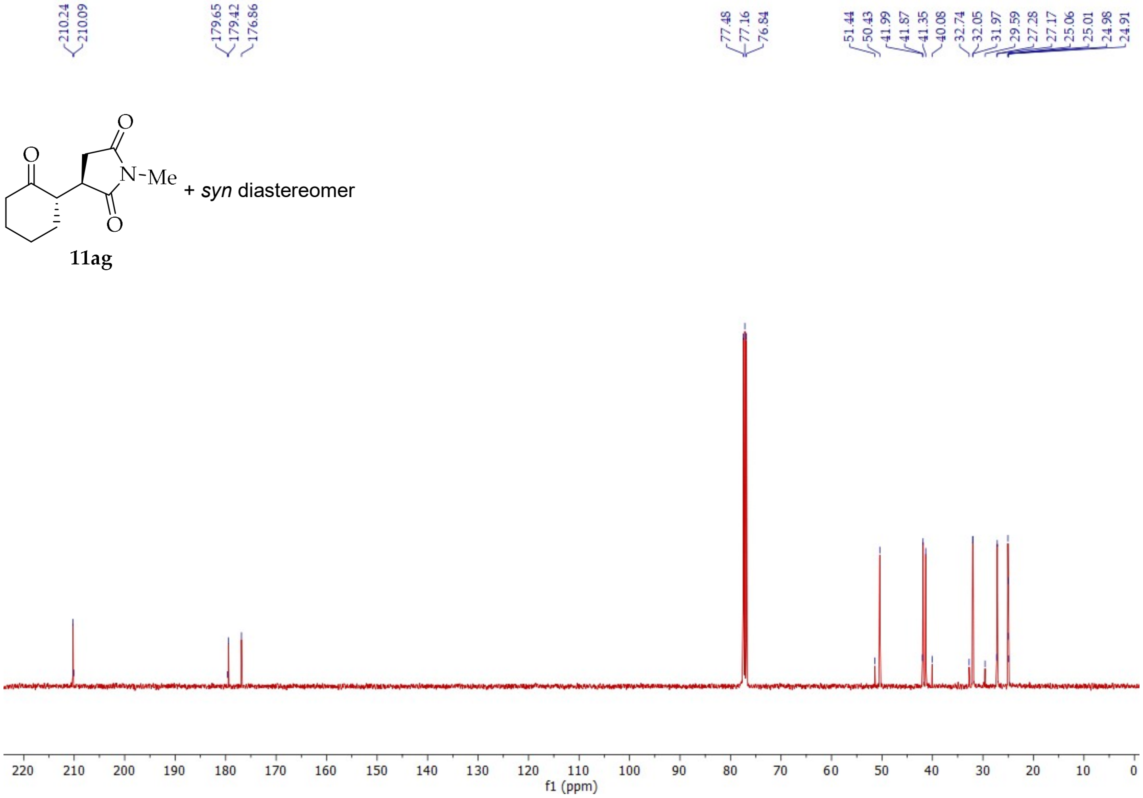

Figure S14. ^{13}C NMR spectrum (101 MHz) of compound **11ag**.

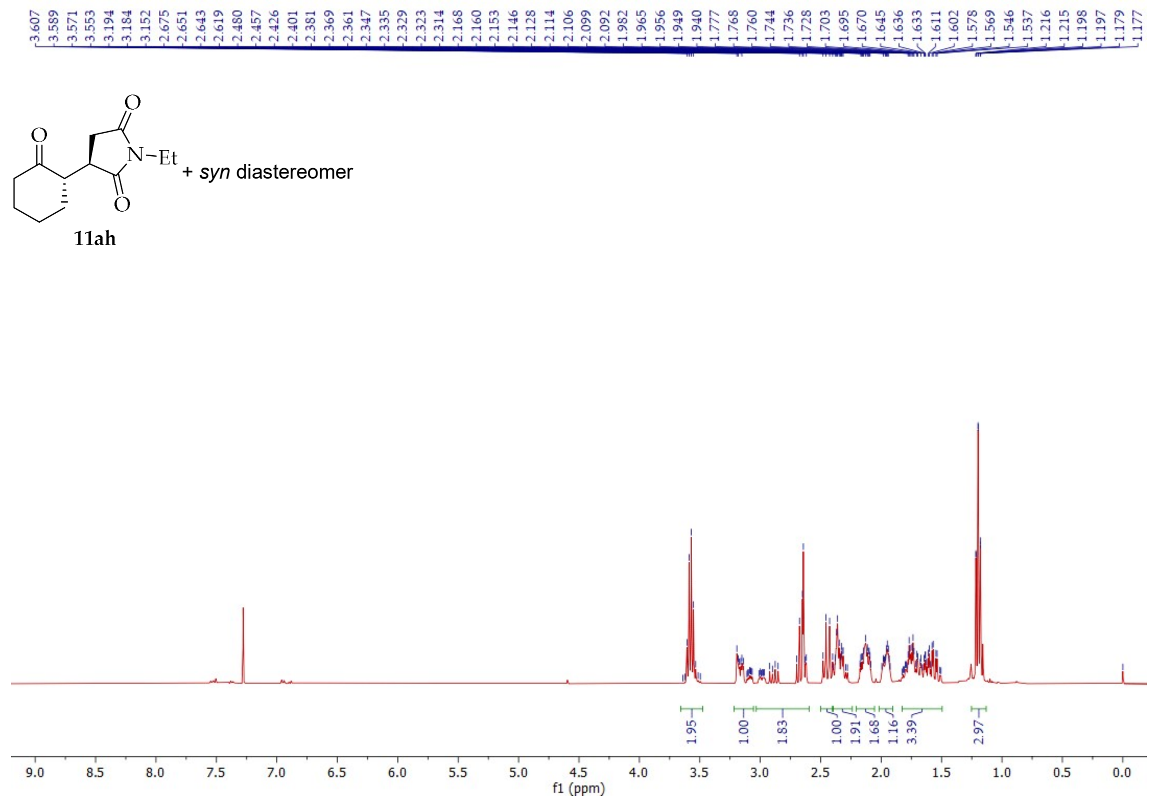

Figure S15. ^{1}H NMR spectrum (400 MHz) of compound **11ah**.

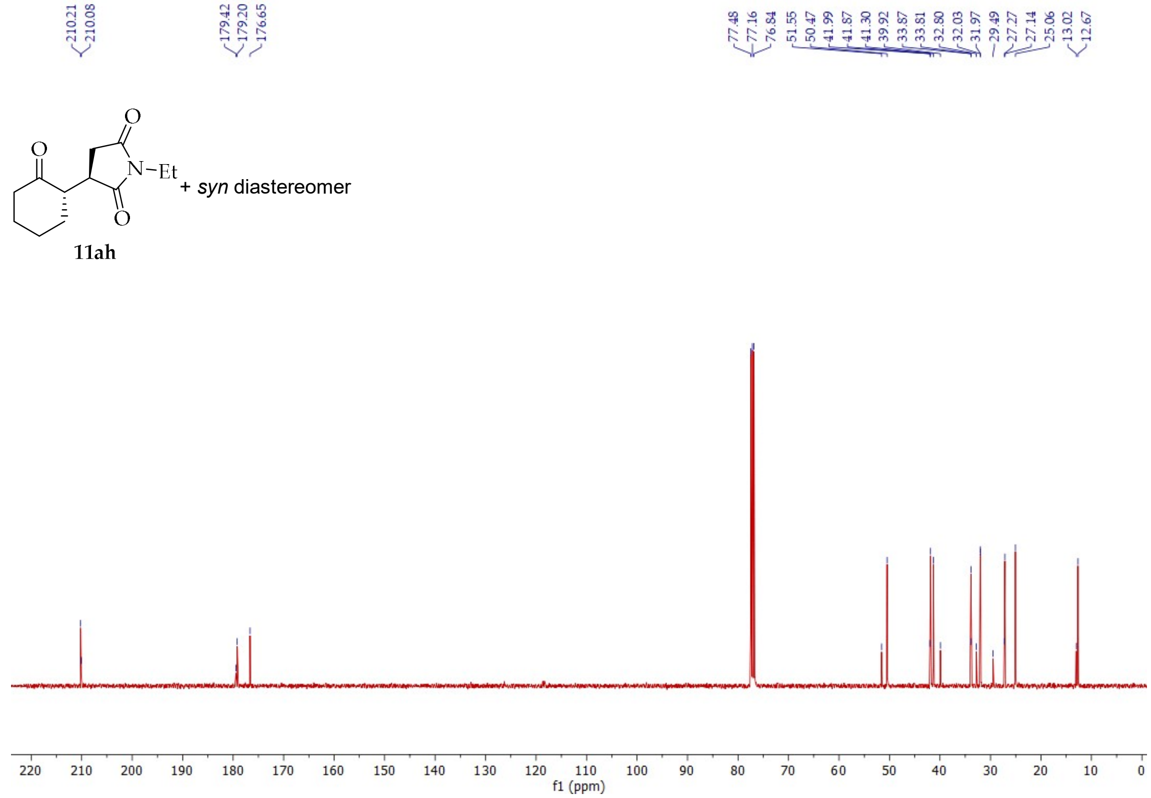

Figure S16. ^{13}C NMR spectrum (101 MHz) of compound **11ah**.

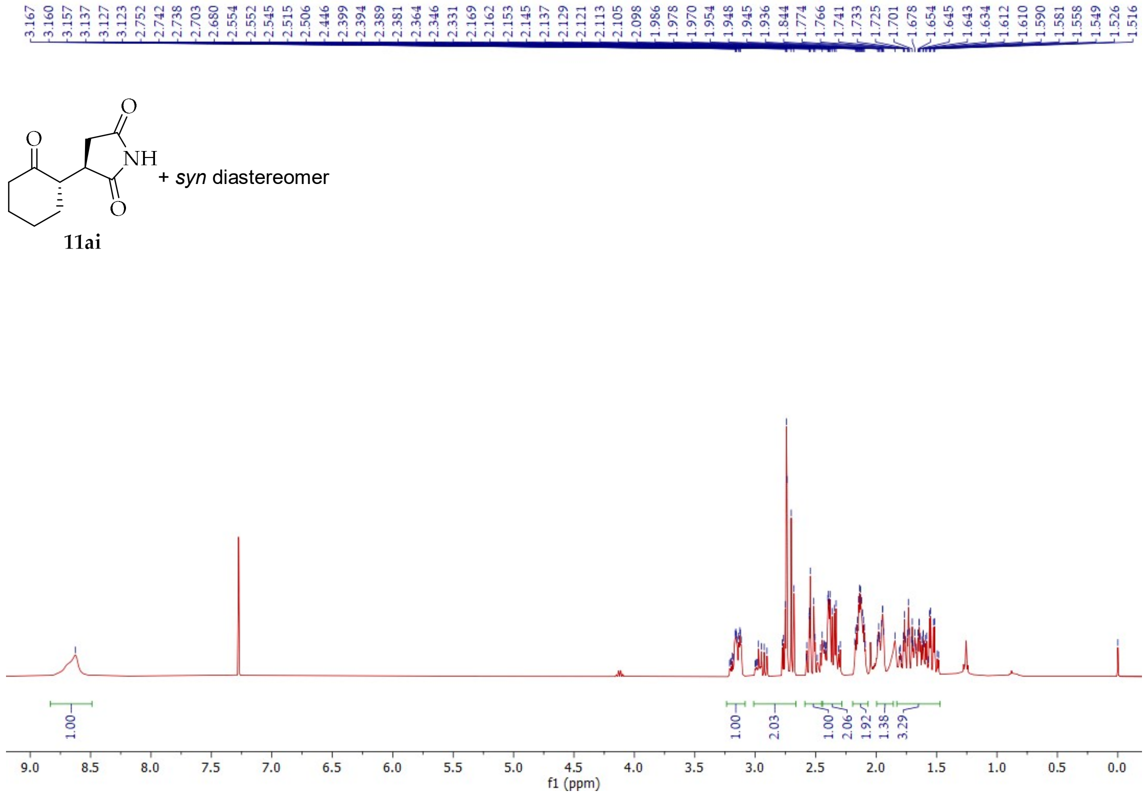

Figure S17. ^{1}H NMR spectrum (400 MHz) of compound **11ai**.

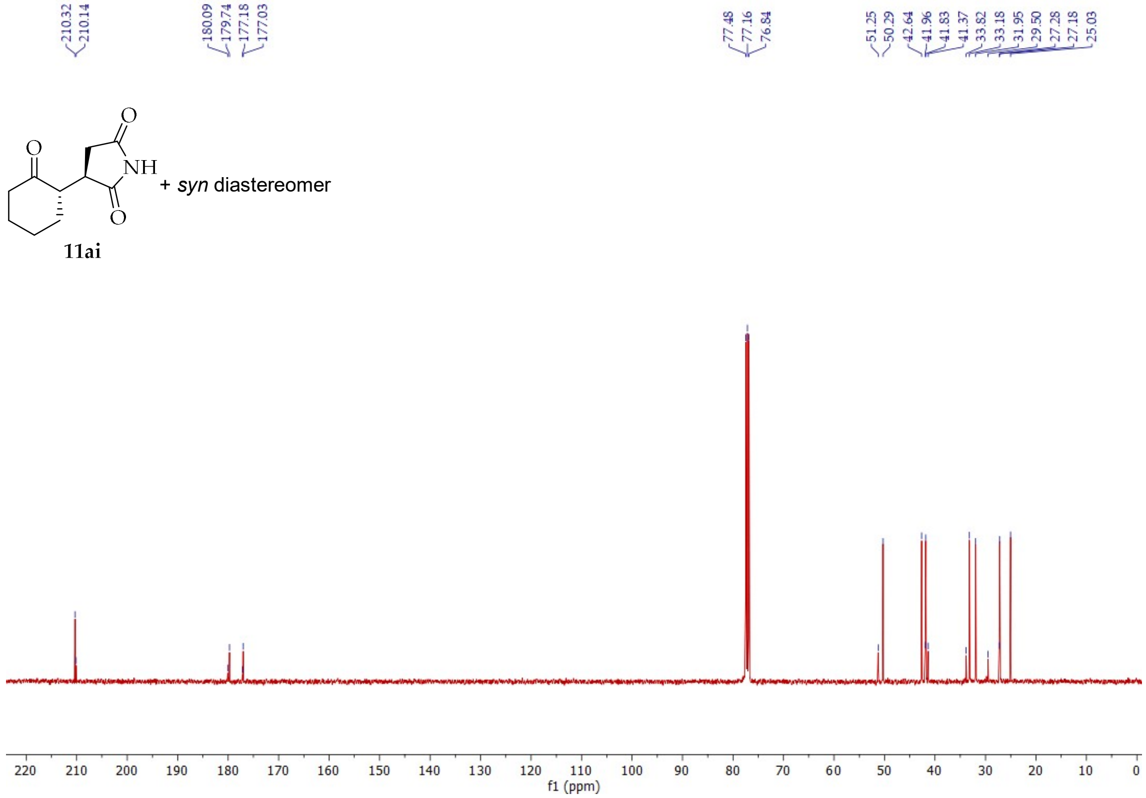

Figure S18. ^{13}C NMR spectrum (101 MHz) of compound **11ai**.

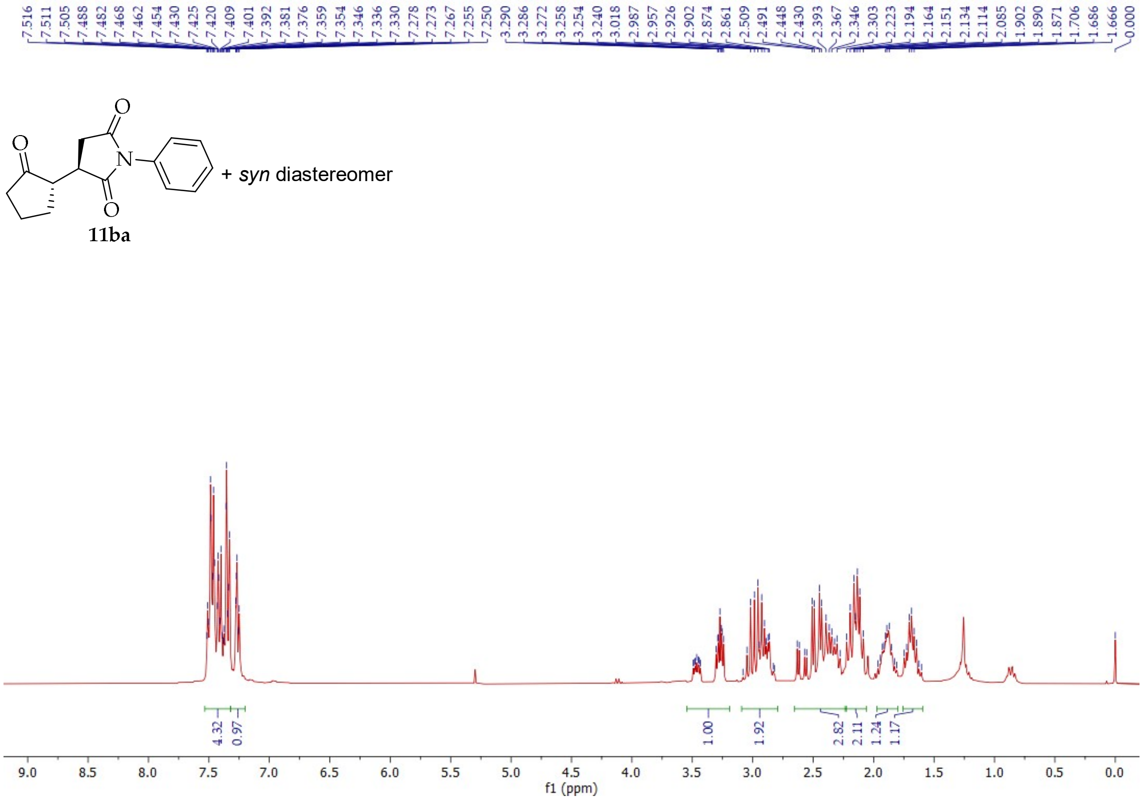

Figure S19. ^{1}H NMR spectrum (300 MHz) of compound **11ba**.

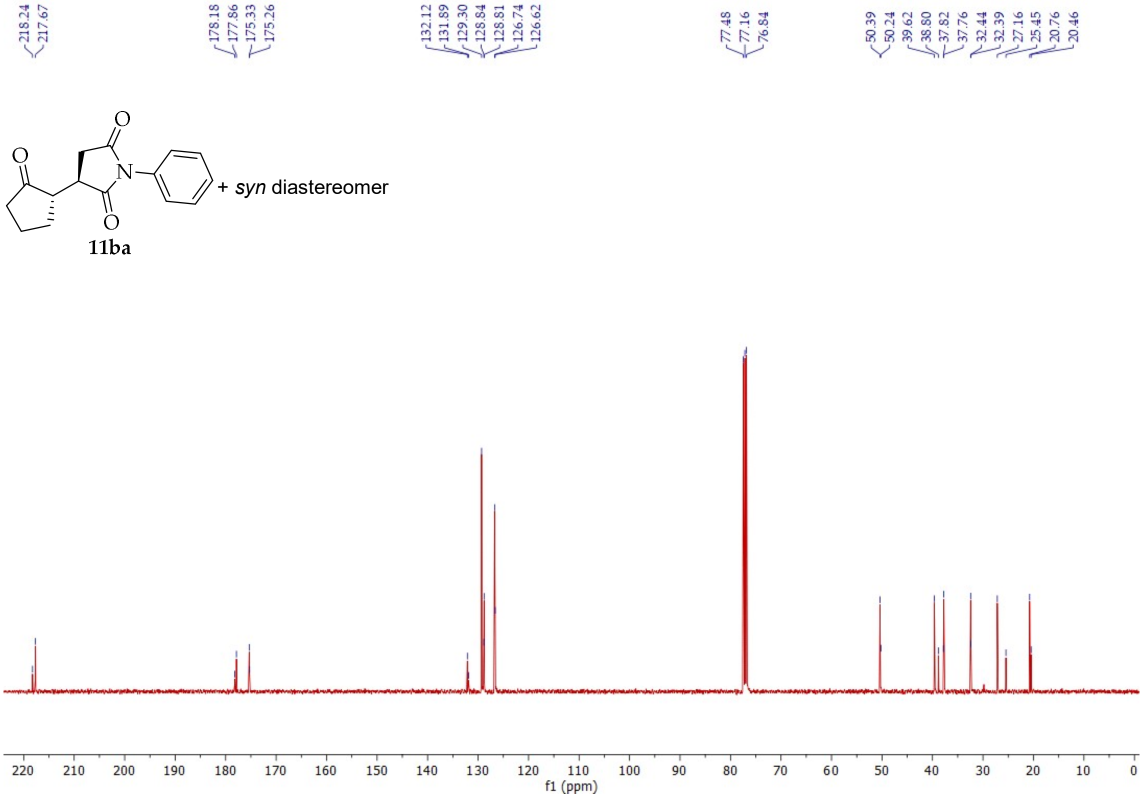

Figure S20. ^{13}C NMR spectrum (75 MHz) of compound **11ba**.

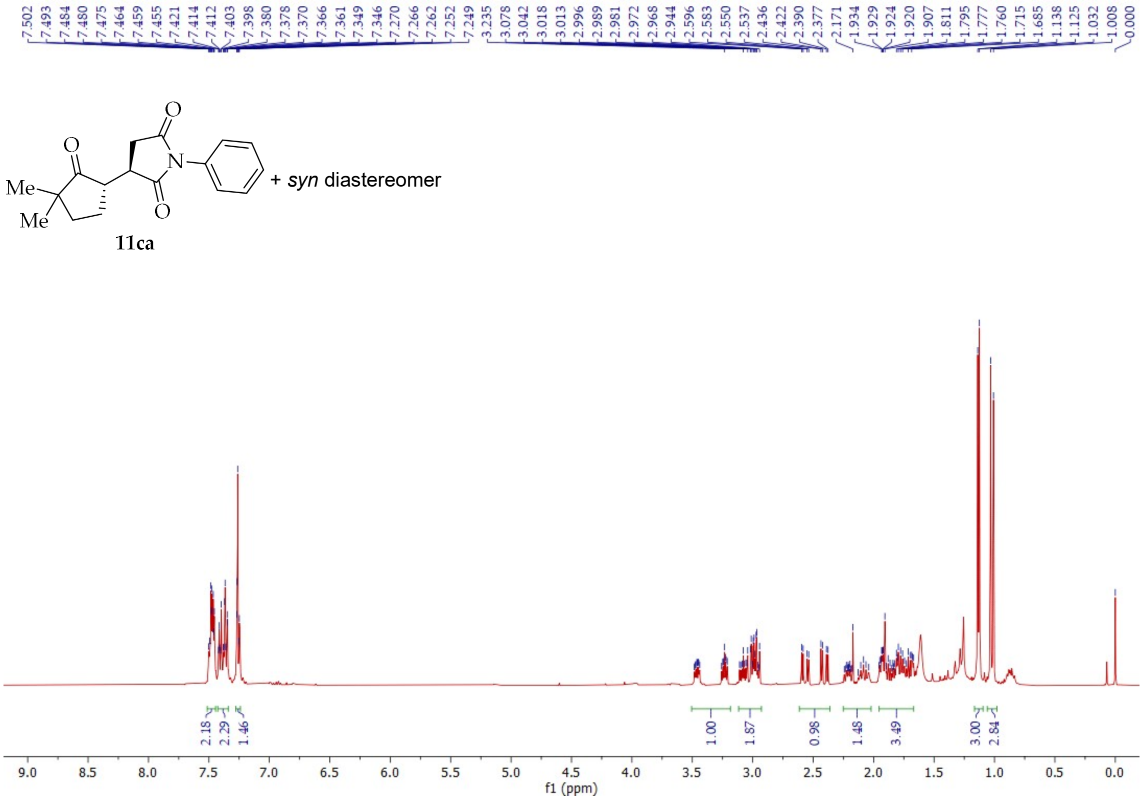

Figure S21. ^{1}H NMR spectrum (400 MHz) of compound **11ca**.

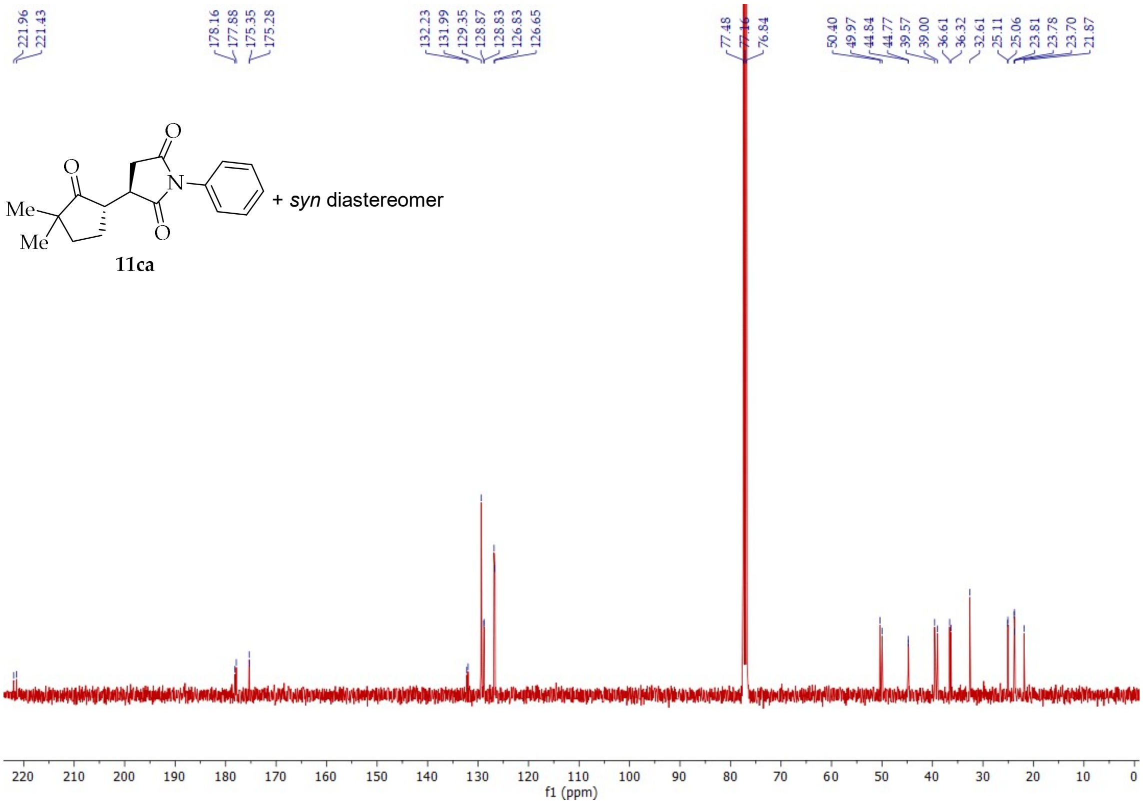

Figure S22. ^{13}C NMR spectrum (101 MHz) of compound **11ca**.

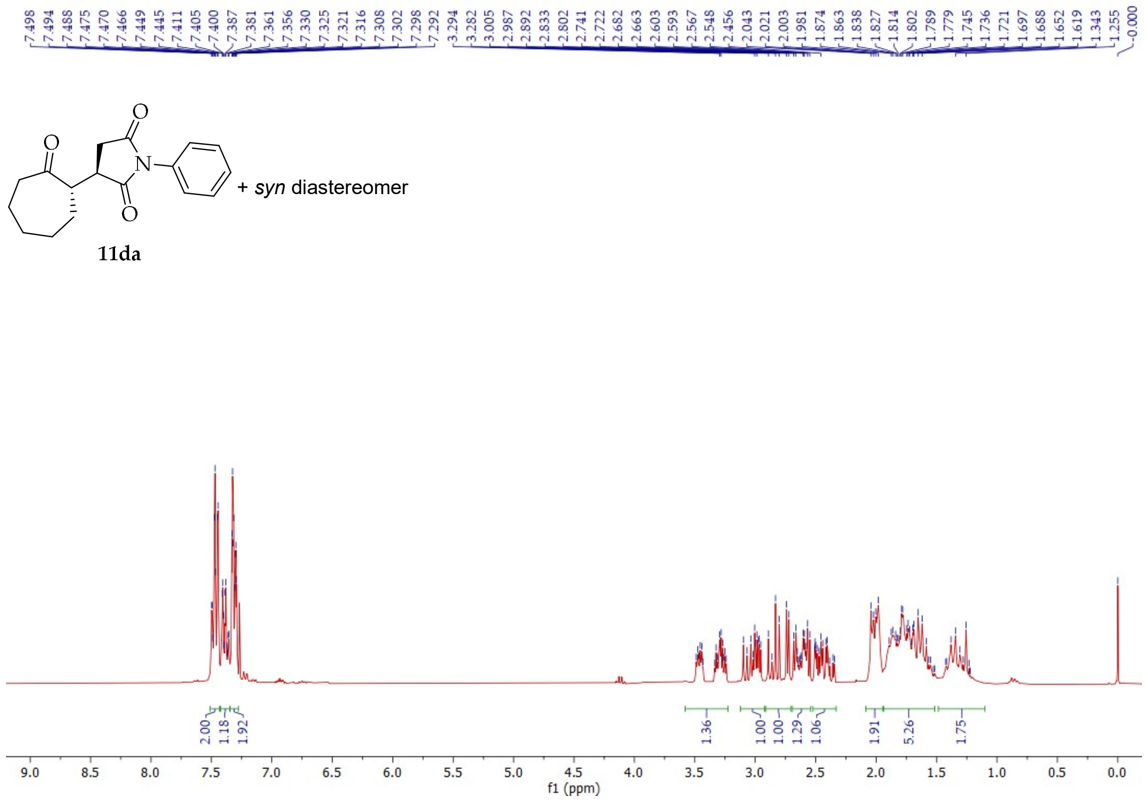

Figure S23. ^{1}H NMR spectrum (300 MHz) of compound **11da**.

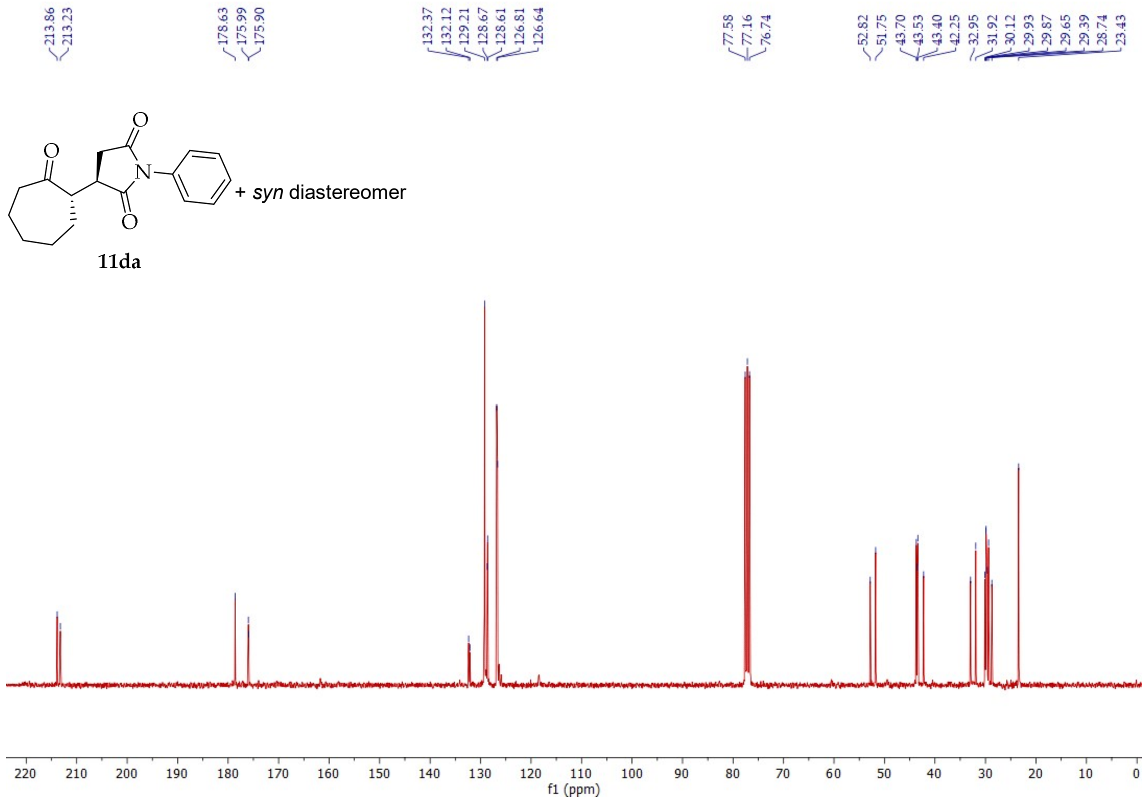

Figure S24. ^{13}C NMR spectrum (75 MHz) of compound **11da**.

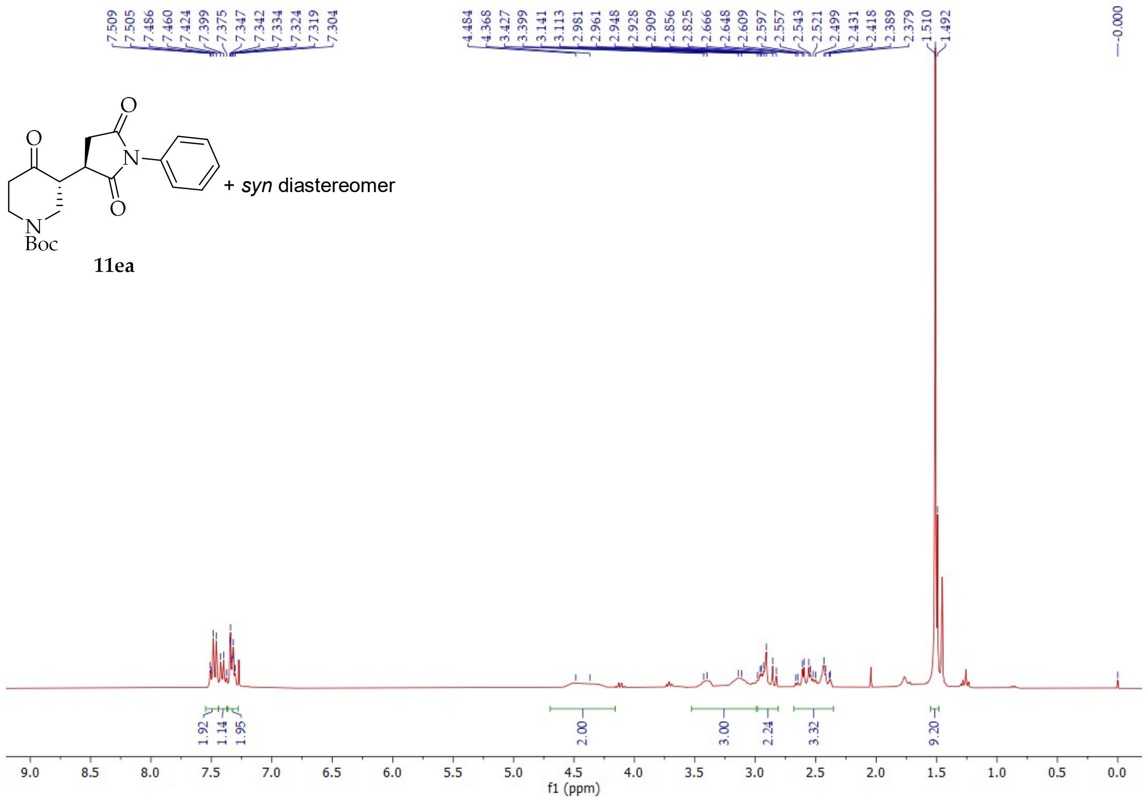

Figure S25. ^{1}H NMR spectrum (300 MHz) of compound **11ea**.

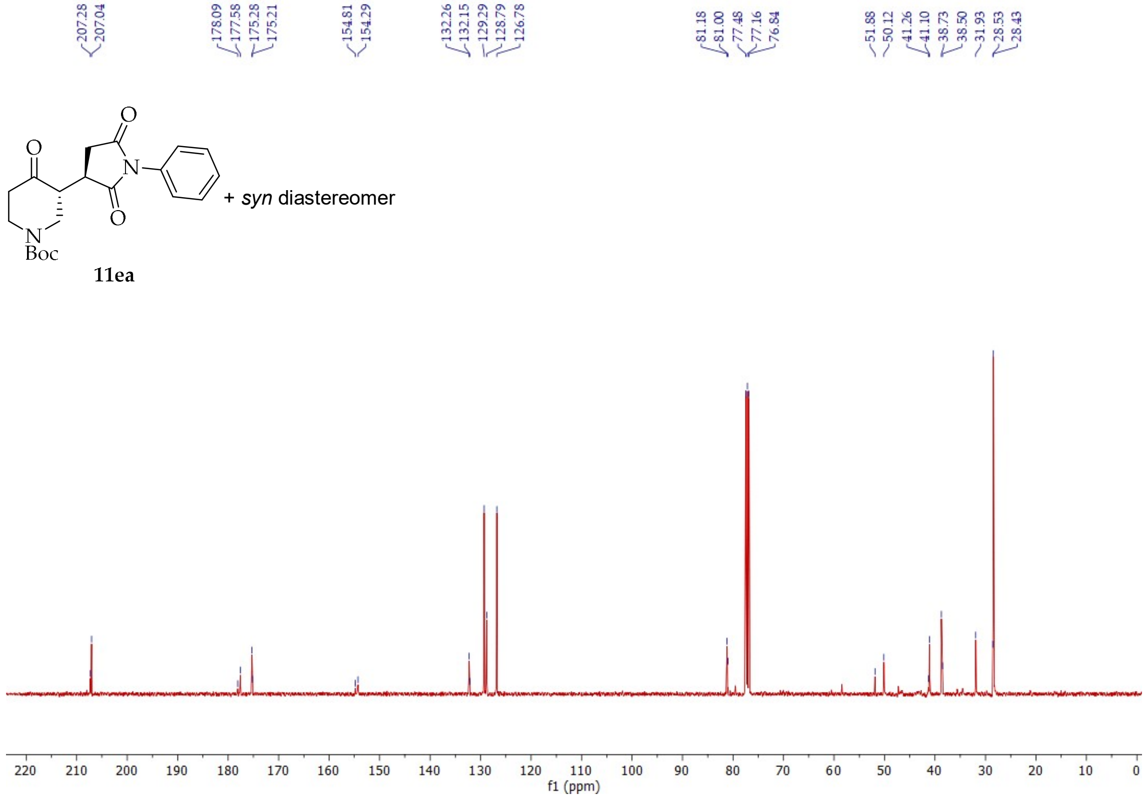

Figure S26. ^{13}C NMR spectrum (75 MHz) of compound **11ea**.

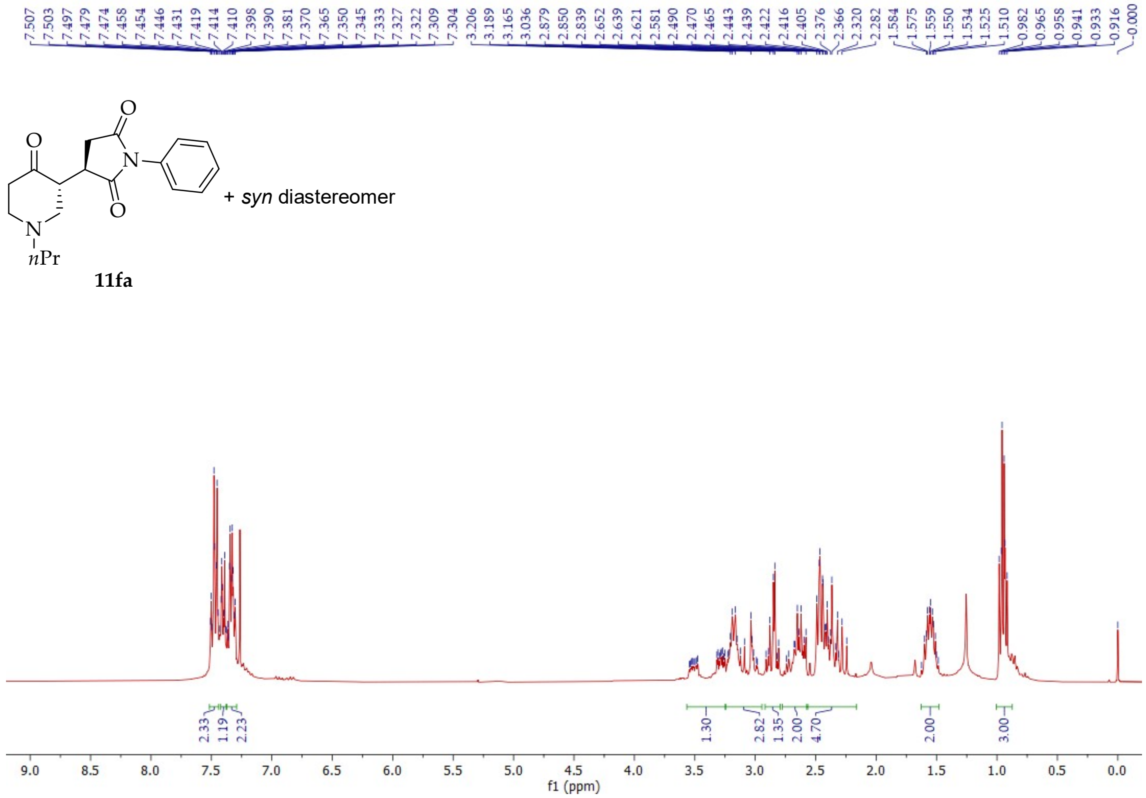

Figure S27. ^{1}H NMR spectrum (300 MHz) of compound **11fa**.

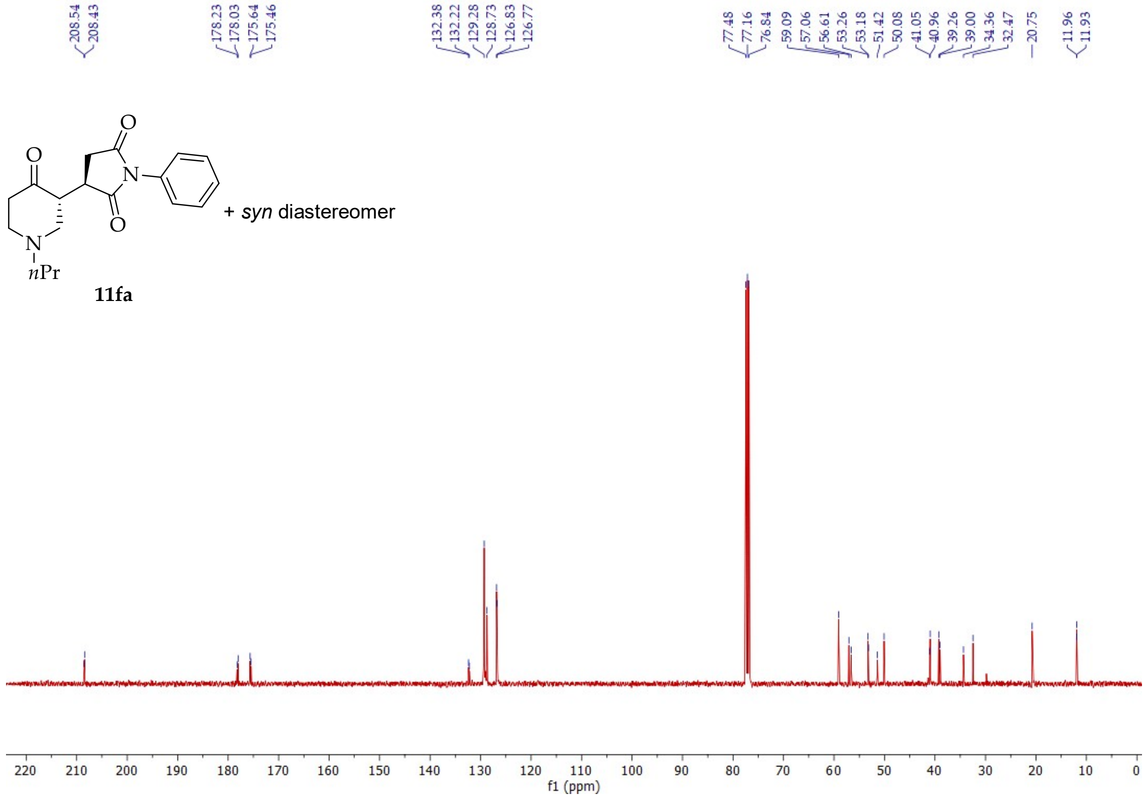

Figure S28. ^{13}C NMR spectrum (75 MHz) of compound **11fa**.

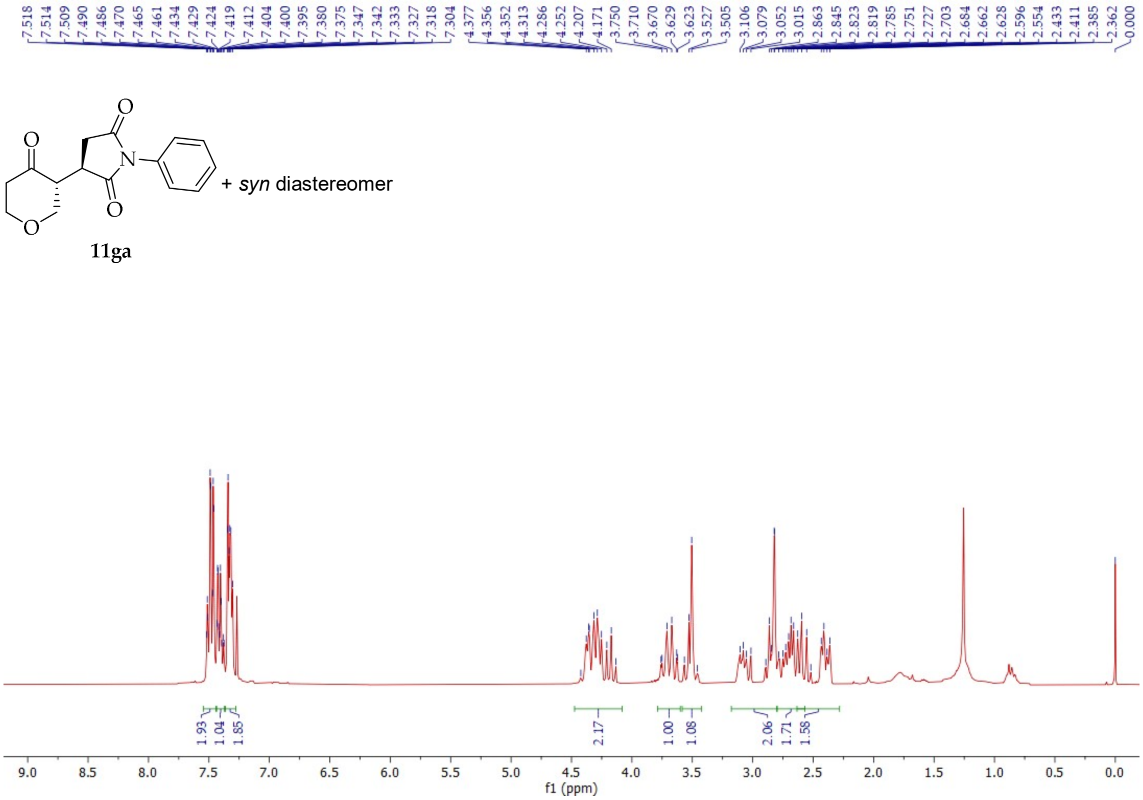

Figure S29. ^{1}H NMR spectrum (300 MHz) of compound **11ga**.

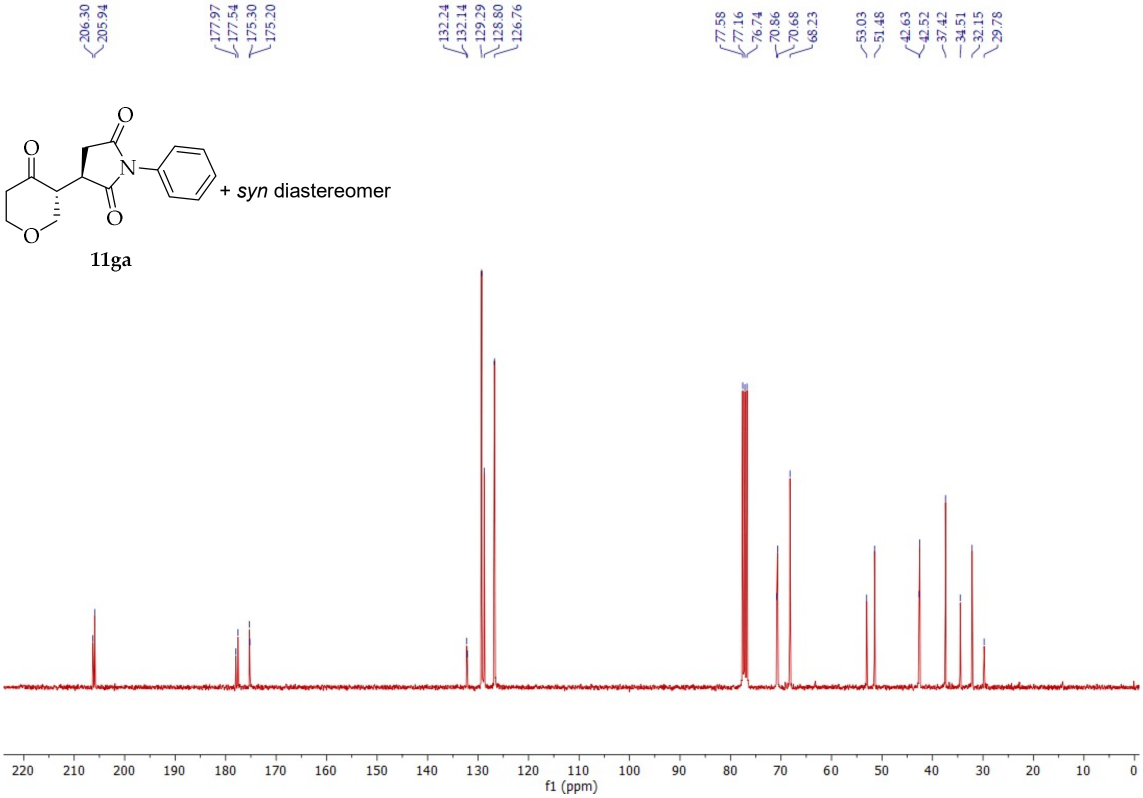

Figure S30. ^{13}C NMR spectrum (75 MHz) of compound **11ga**.

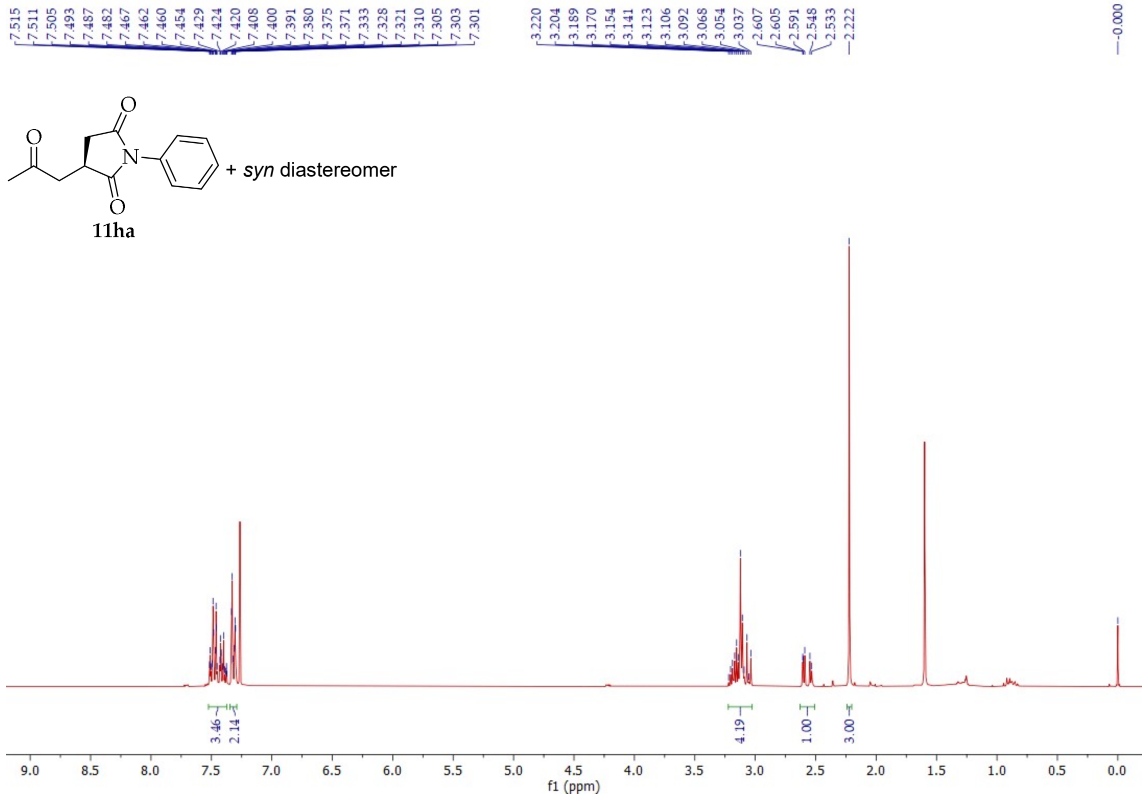

Figure S31. ^{1}H NMR spectrum (300 MHz) of compound **11ha**.

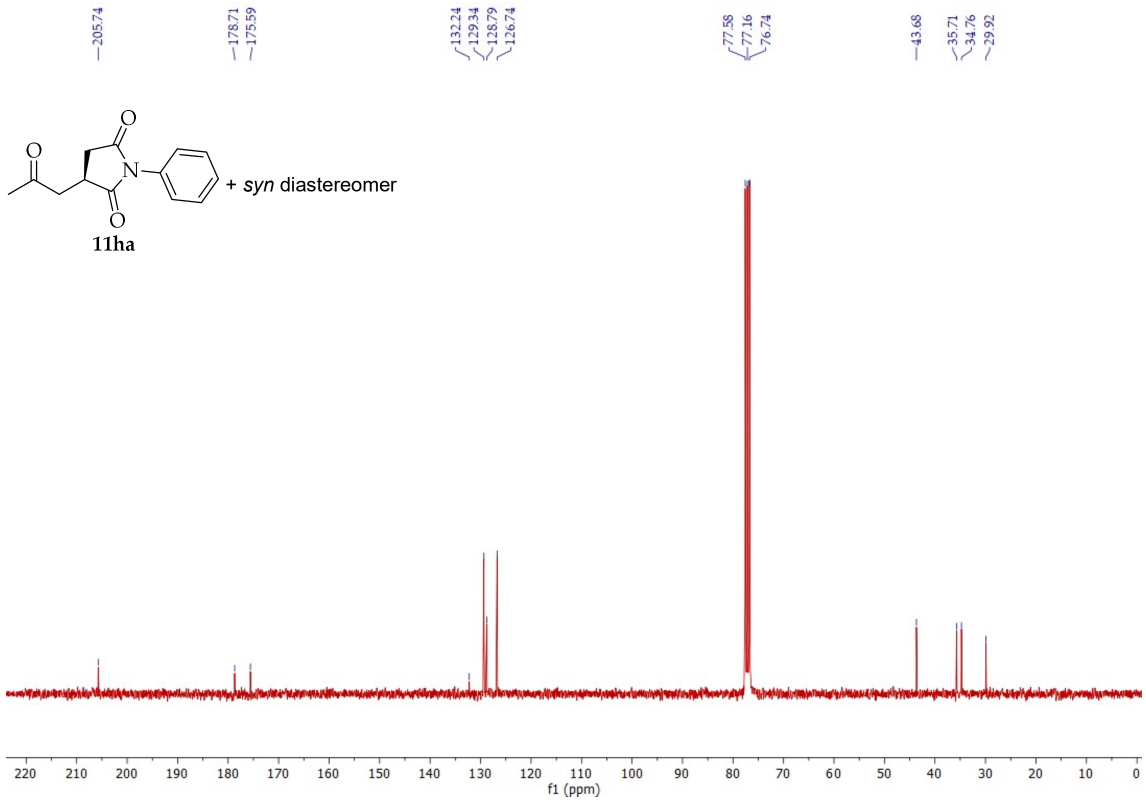

Figure S32. ^{13}C NMR spectrum (75 MHz) of compound **11ha**.

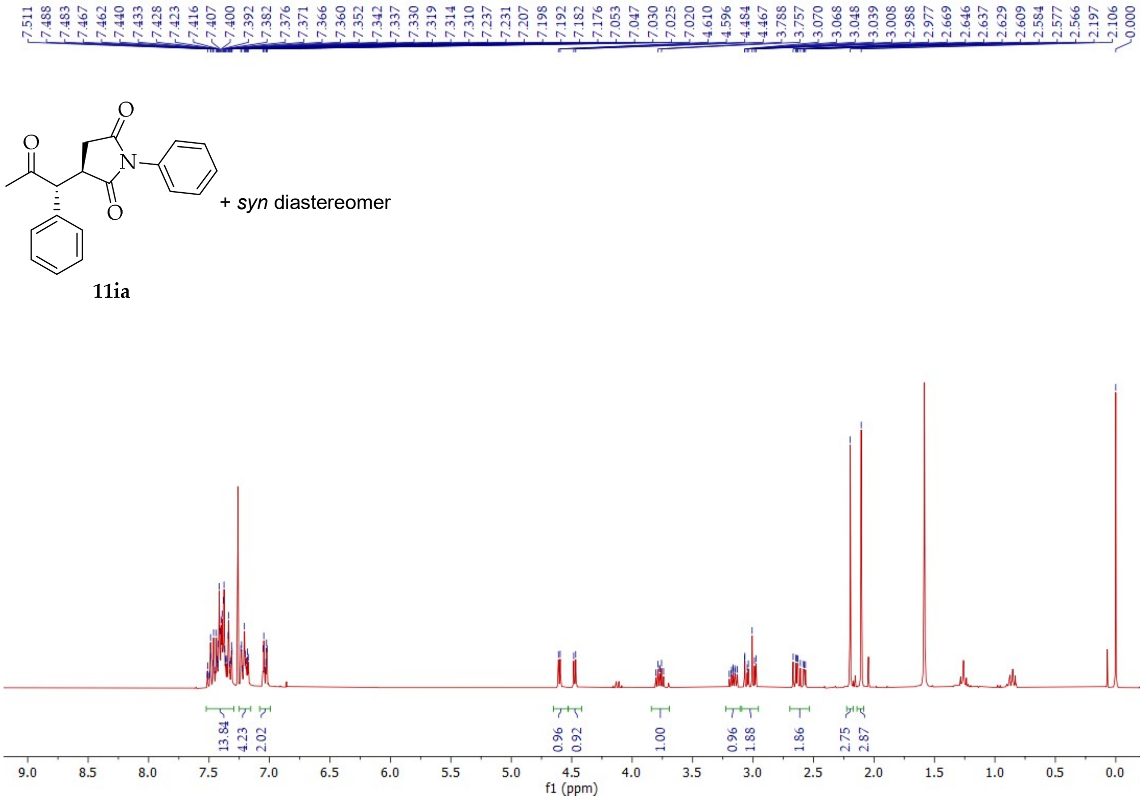

Figure S33. ^{1}H NMR spectrum (300 MHz) of compound **11ia**.

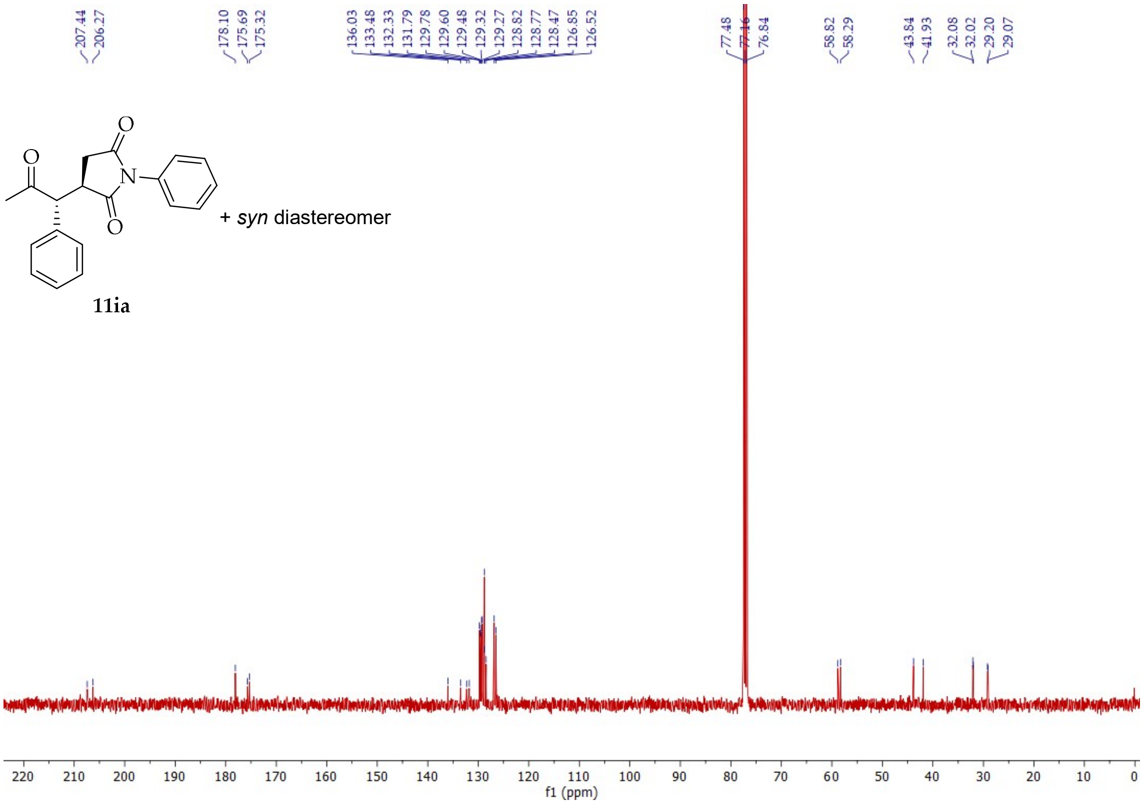

Figure S34. ^{13}C NMR spectrum (75 MHz) of compound **11ia**.

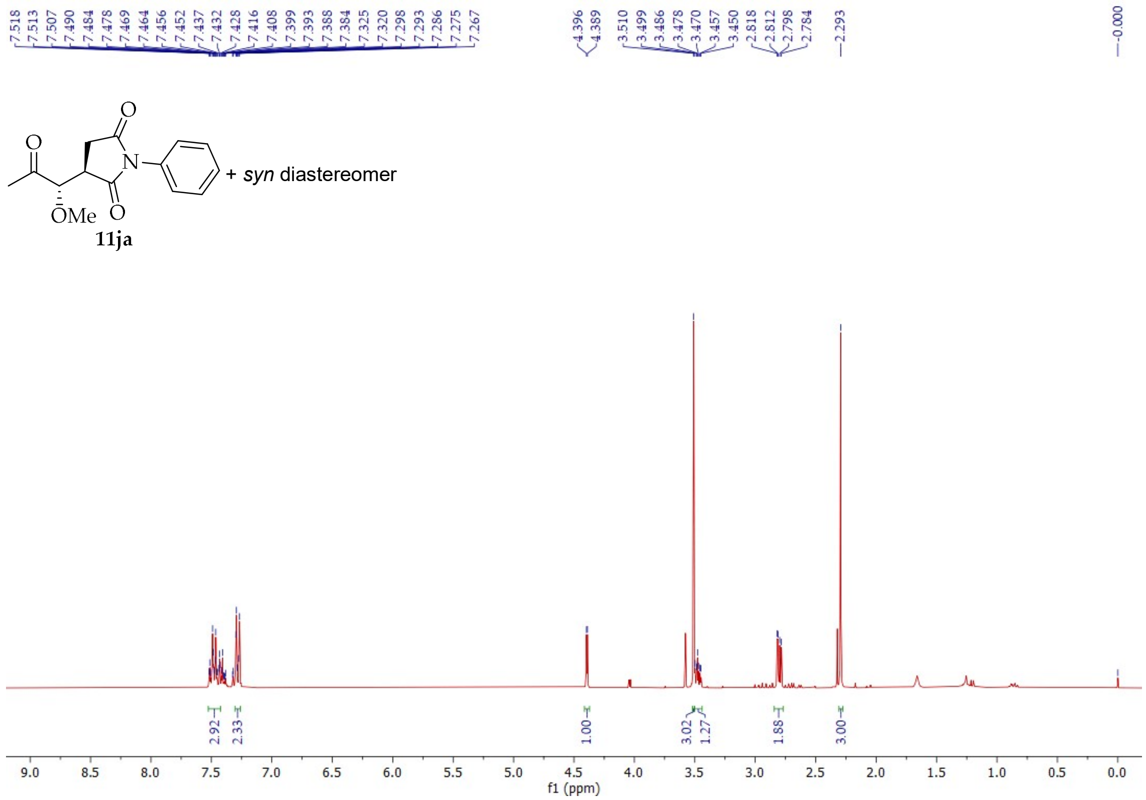

Figure S35. ^{1}H NMR spectrum (300 MHz) of compound **11ja**.

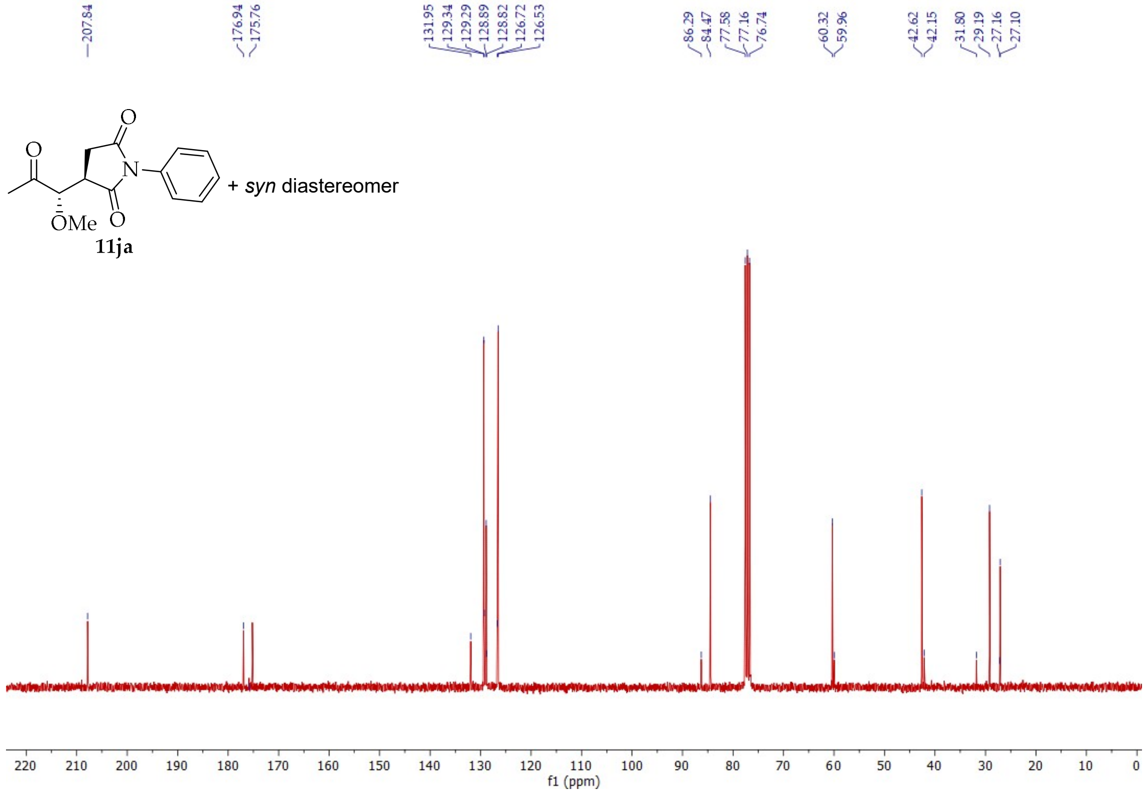

Figure S36. ^{13}C NMR spectrum (75 MHz) of compound **11ja**.

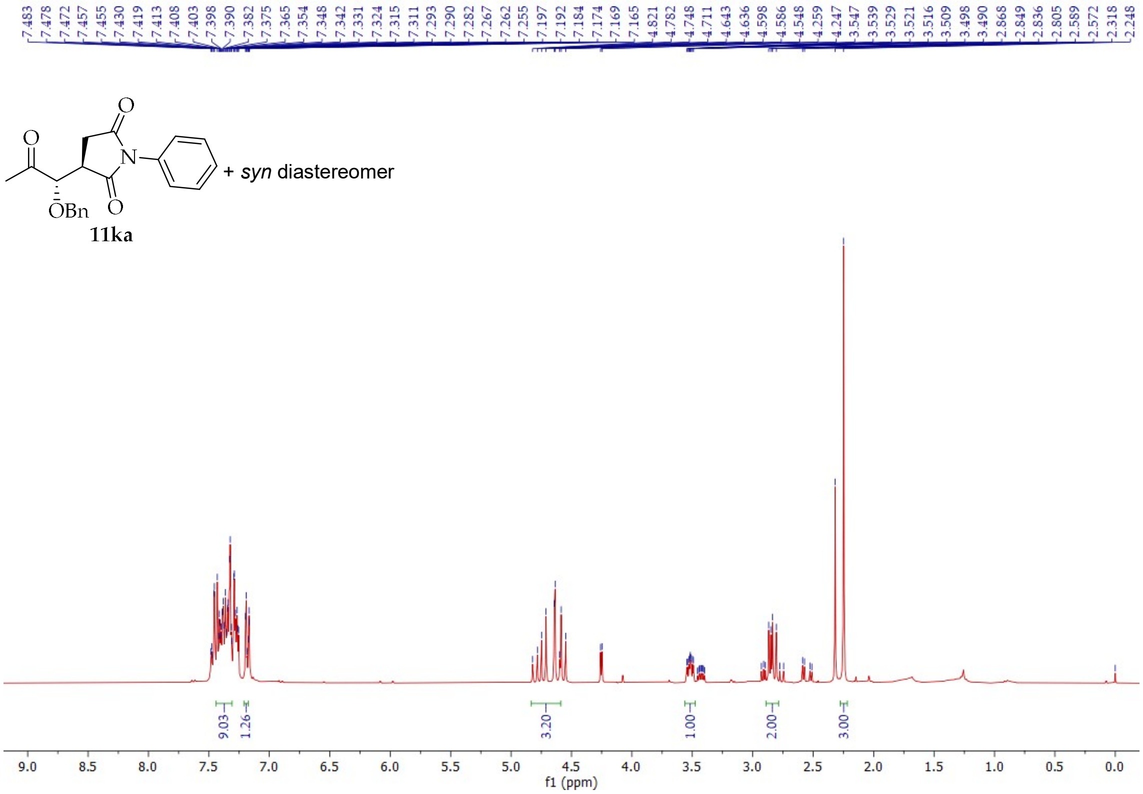

Figure S37. ^{1}H NMR spectrum (300 MHz) of compound **11ka**.

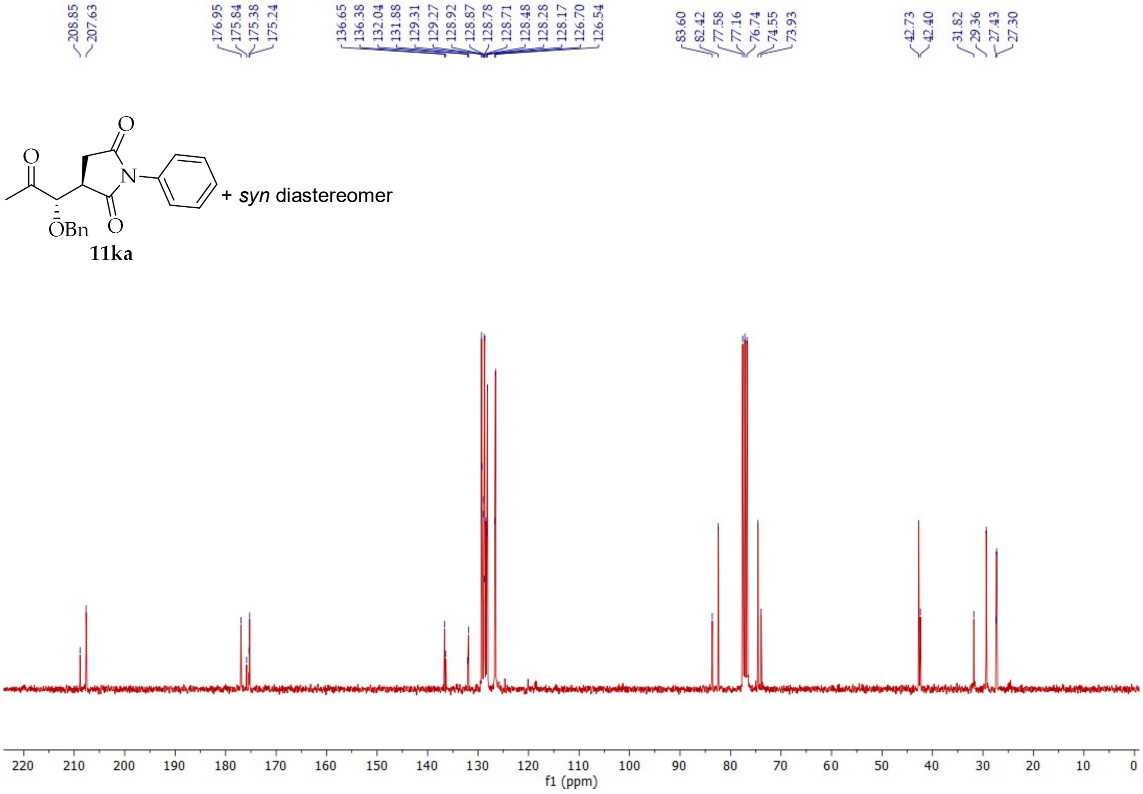

Figure S38. ^{13}C NMR spectrum (75 MHz) of compound **11ka**.

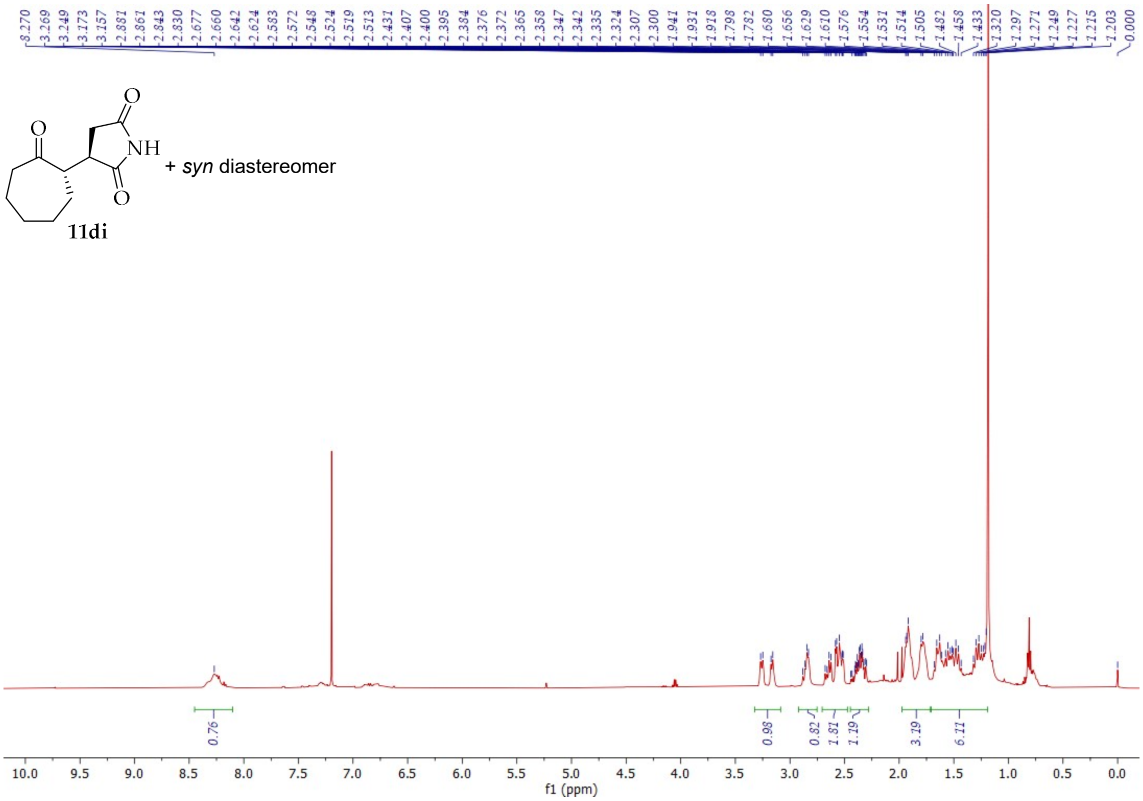

Figure S39. ^{1}H NMR spectrum (500 MHz) of compound **11di**.

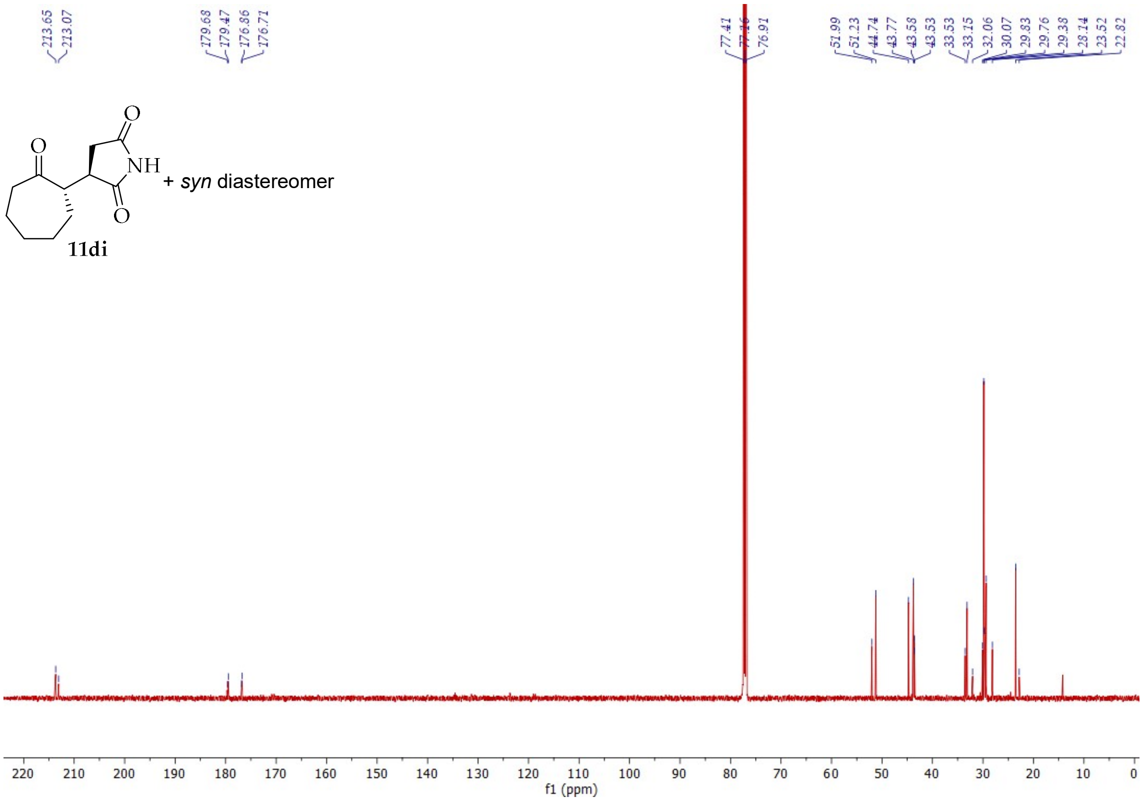

Figure S40. ^{13}C NMR spectrum (125 MHz) of compound **11di**.

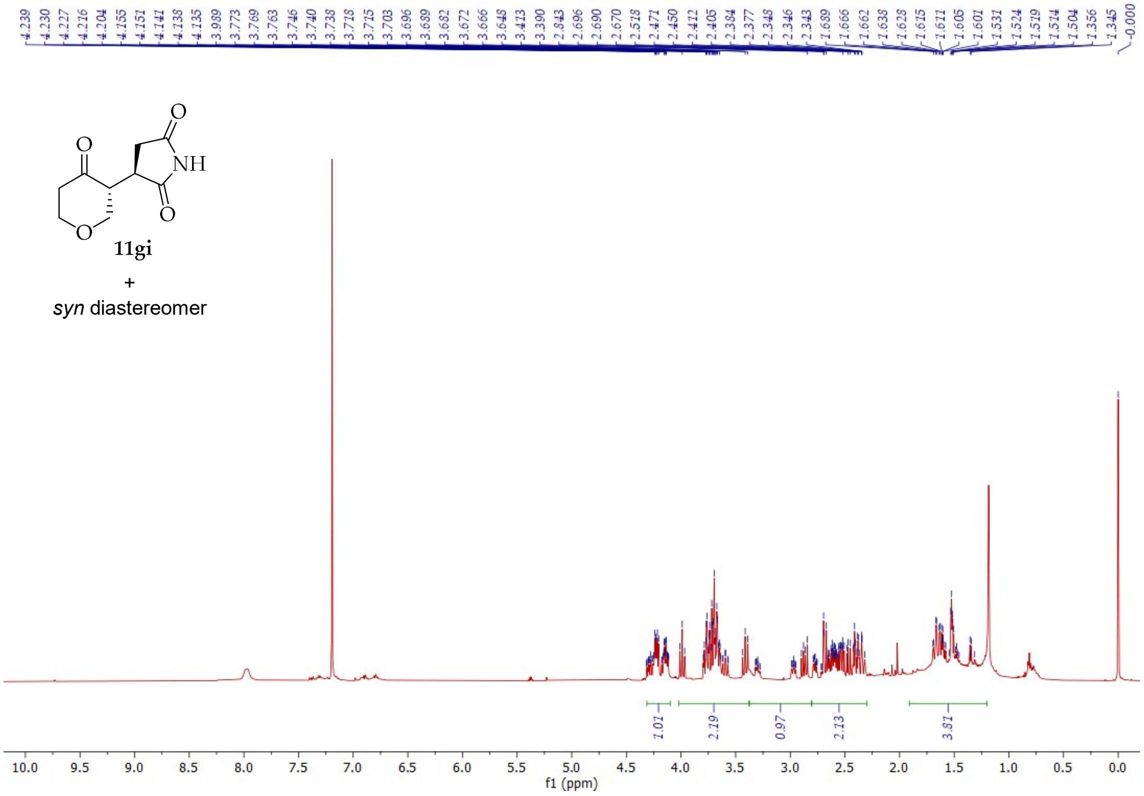

Figure S41. ^{1}H NMR spectrum (500 MHz) of compound **11gi**.

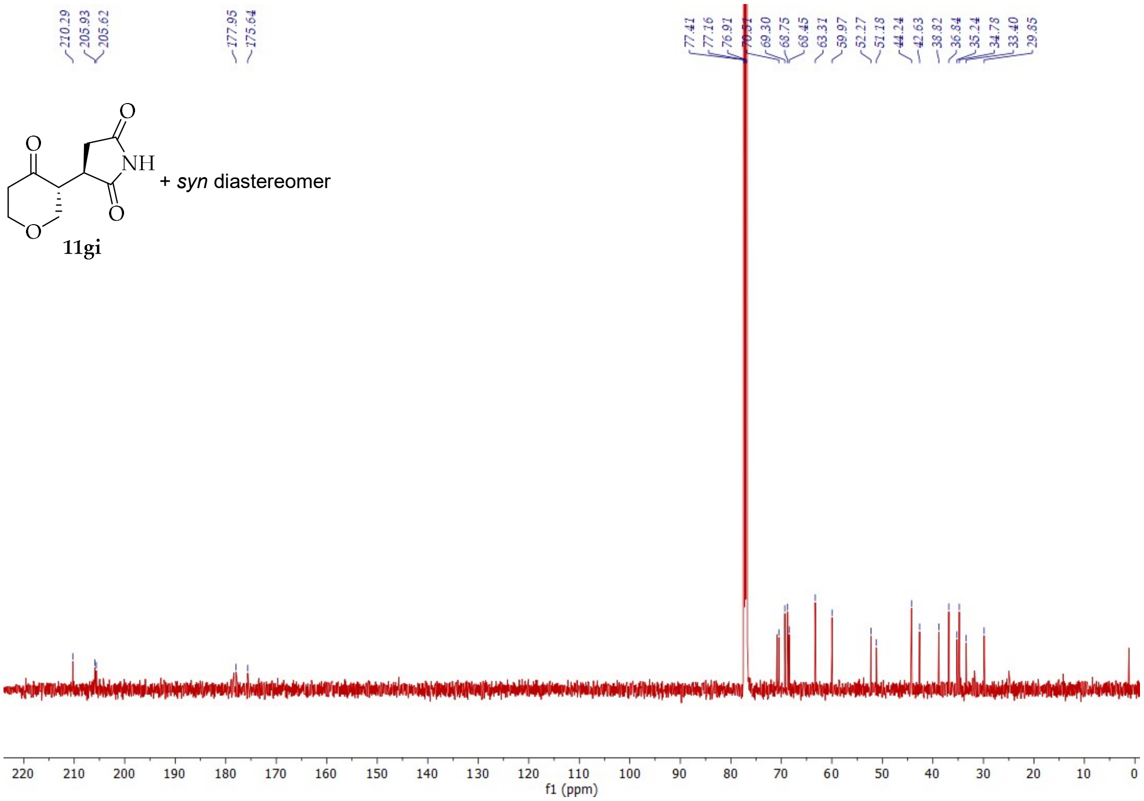

Figure S42. ^{13}C NMR spectrum (125 MHz) of compound **11gi**.

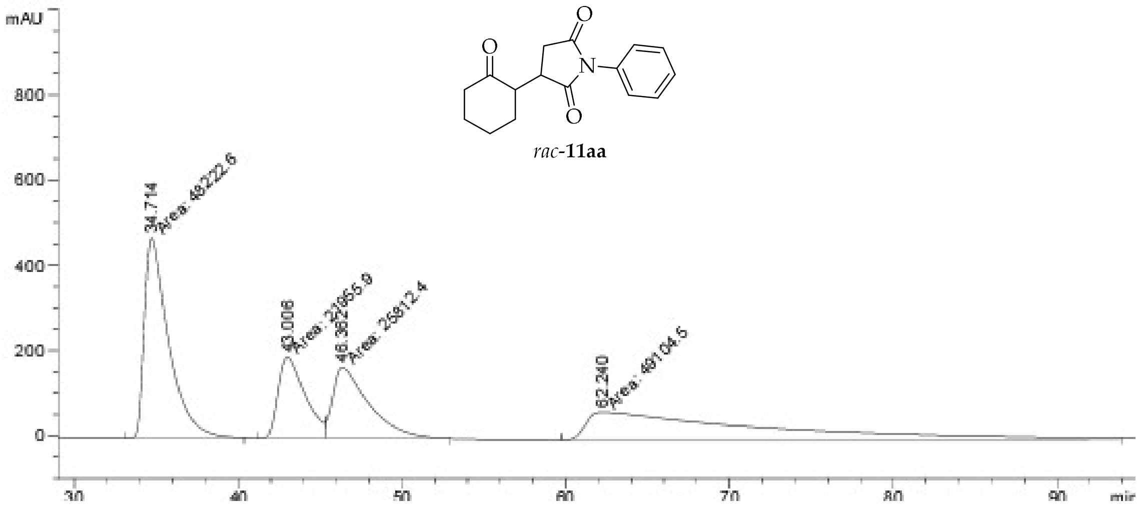

Peak #	RetTime [min]	Type	Width [min]	Area [mAU*s]	Height [mAU]	Area %
1	34.714	MM	1.7149	4.82226e4	468.67322	33.2351
2	43.006	MF	1.9246	2.19559e4	190.13069	15.1320
3	46.362	FM	2.5638	2.58124e4	167.80072	17.7899
4	62.240	MM	12.2222	4.91045e4	66.96054	33.8429

Figure S43. HPLC chromatogram of compound *rac*-**11aa**.

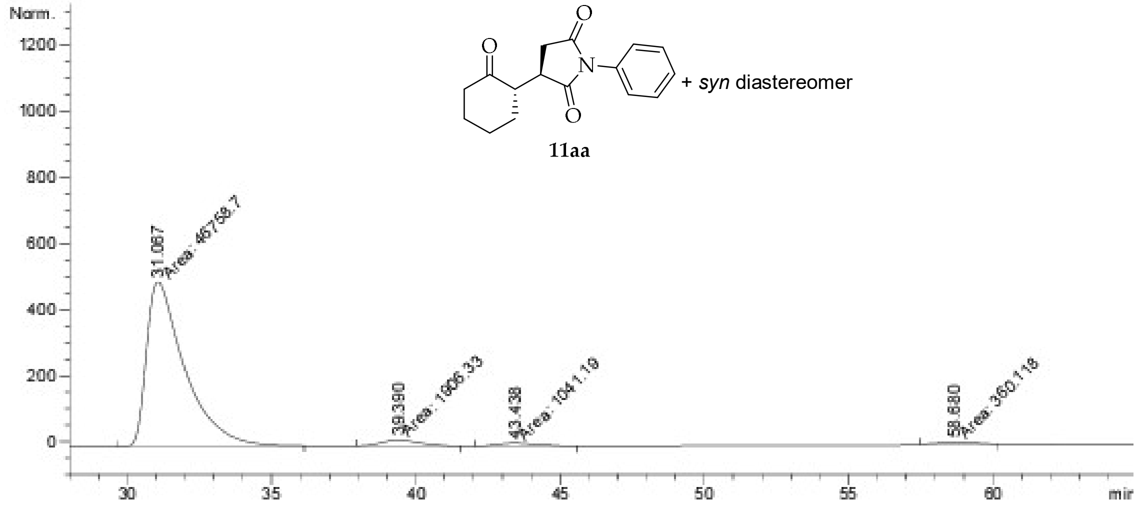

Peak #	RetTime [min]	Type	Width [min]	Area [mAU*s]	Height [mAU]	Area %
1	31.067	MM	1.5595	4.67587e4	499.71292	93.3935
2	39.390	MM	1.6461	1906.33228	19.30093	3.8076
3	43.438	MM	1.7359	1041.18604	9.99647	2.0796
4	58.680	MM	1.4173	360.11752	4.23475	0.7193

Figure S44. HPLC chromatogram of compound **11aa**.

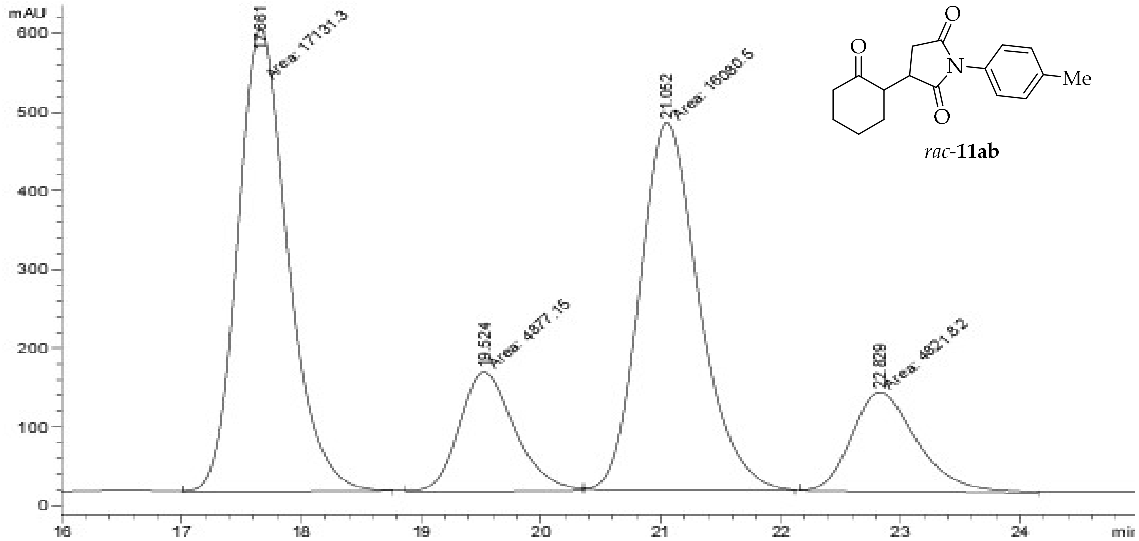

Peak #	RetTime [min]	Type	Width [min]	Area [mAU*s]	Height [mAU]	Area %
1	17.661	MM	0.4856	1.71313e4	588.02008	39.9230
2	19.524	MM	0.5357	4877.14990	151.74623	11.3658
3	21.052	MM	0.5745	1.60805e4	466.54474	37.4744
4	22.829	MM	0.6381	4821.82324	125.94037	11.2369

Figure S45. HPLC chromatogram of compound *rac*-**11ab**.

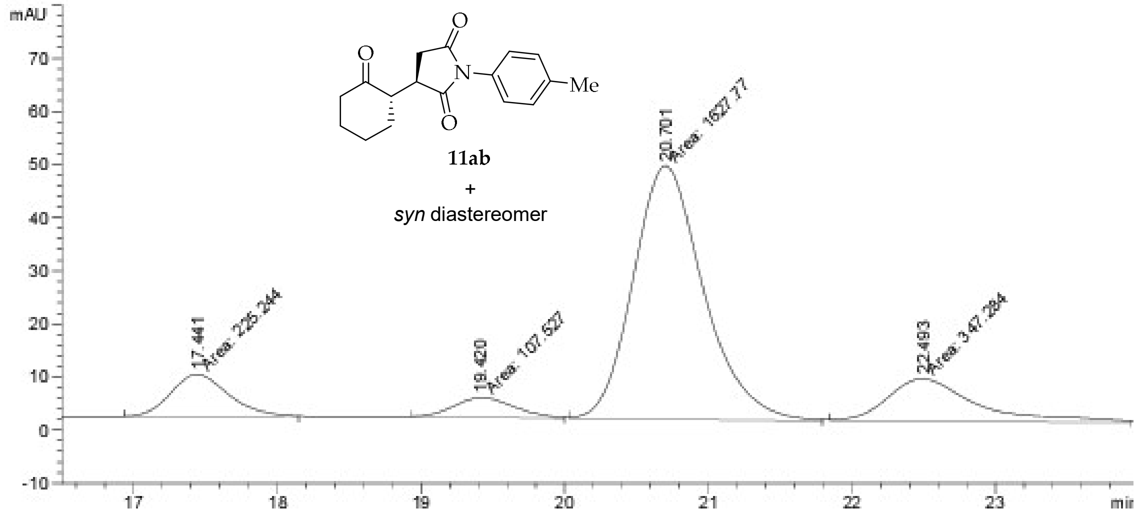

Peak #	RetTime [min]	Type	Width [min]	Area [mAU*s]	Height [mAU]	Area %
1	17.441	MM	0.4697	225.24380	7.99219	9.7600
2	19.420	MM	0.4856	107.52734	3.69019	4.6593
3	20.701	MM	0.5685	1627.76746	47.71959	70.5326
4	22.493	MM	0.7207	347.28403	8.03147	15.0481

Figure S46. HPLC chromatogram of compound **11ab**.

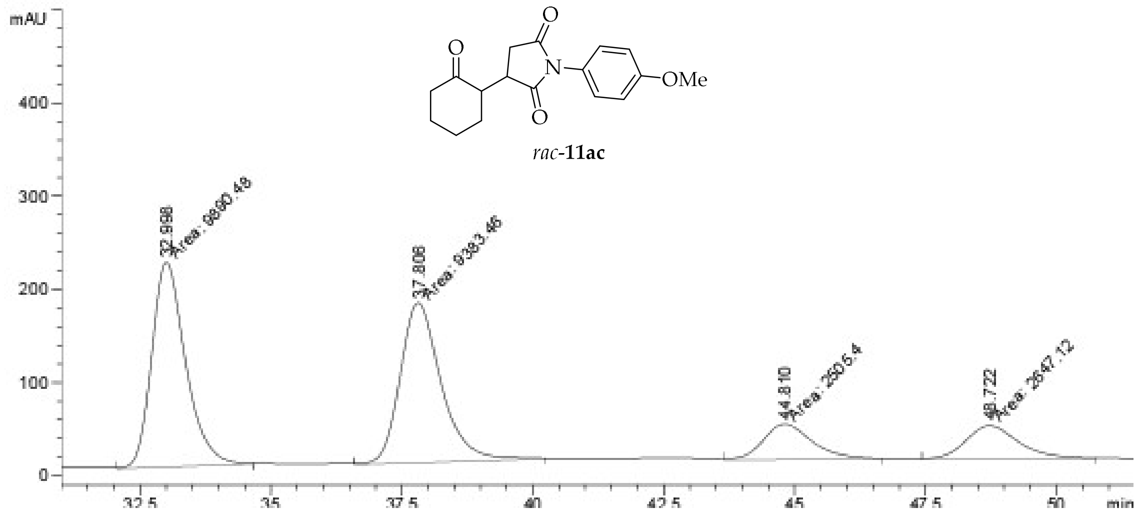

Peak #	RetTime [min]	Type	Width [min]	Area [mAU*s]	Height [mAU]	Area %
1	32.998	MM	0.7514	9890.47559	219.38046	40.4908
2	37.806	MM	0.9110	9383.46289	171.66896	38.4152
3	44.810	MM	1.0966	2505.39697	38.07766	10.2569
4	48.722	MM	1.2248	2647.12402	36.02127	10.8371

Figure S47. HPLC chromatogram of compound *rac*-**11ac**.

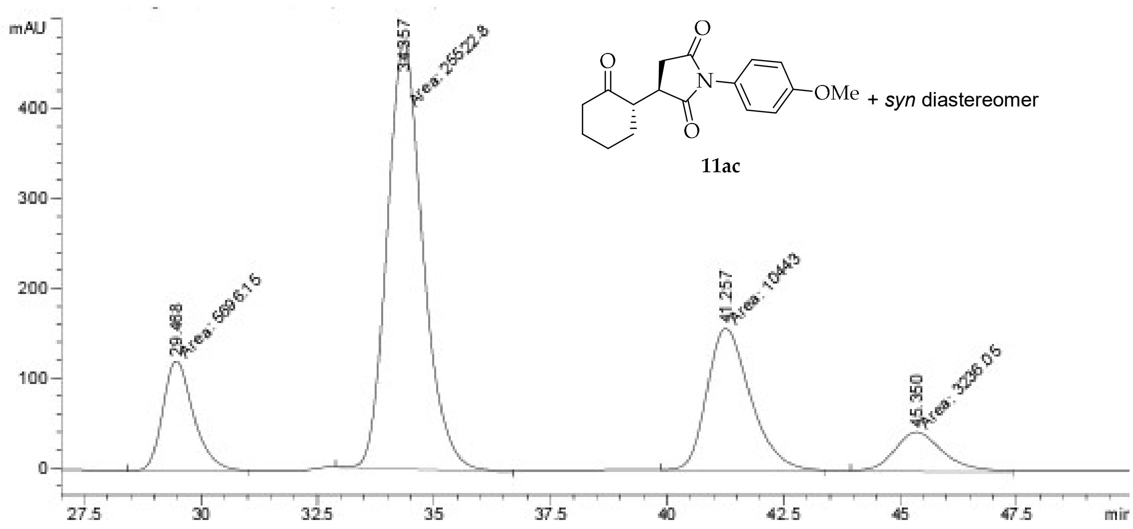

Peak #	RetTime [min]	Type	Width [min]	Area [mAU*s]	Height [mAU]	Area %
1	29.468	MM	0.7725	5696.15430	122.89522	12.6869
2	34.357	MM	0.8963	2.55228e4	474.61026	56.8461
3	41.257	MM	1.0962	1.04430e4	158.77977	23.2594
4	45.350	MM	1.2296	3236.05103	43.86297	7.2076

Figure S48. HPLC chromatogram of compound **11ac**.

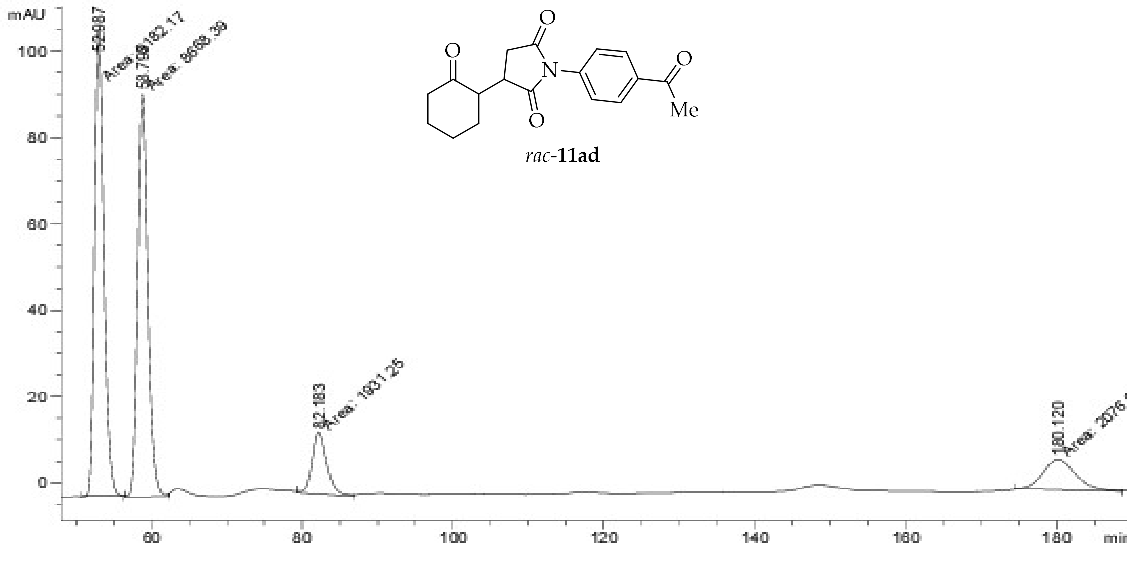

Peak #	RetTime [min]	Type	Width [min]	Area [mAU*s]	Height [mAU]	Area %
1	52.987	MM	1.4171	9182.17480	107.99545	42.0069
2	58.793	MM	1.5471	8668.38672	93.38093	39.6564
3	82.183	MM	2.2626	1931.25232	14.22615	8.8351
4	180.120	MM	4.9548	2076.92920	6.98622	9.5016

Figure S49. HPLC chromatogram of compound *rac*-**11ad**.

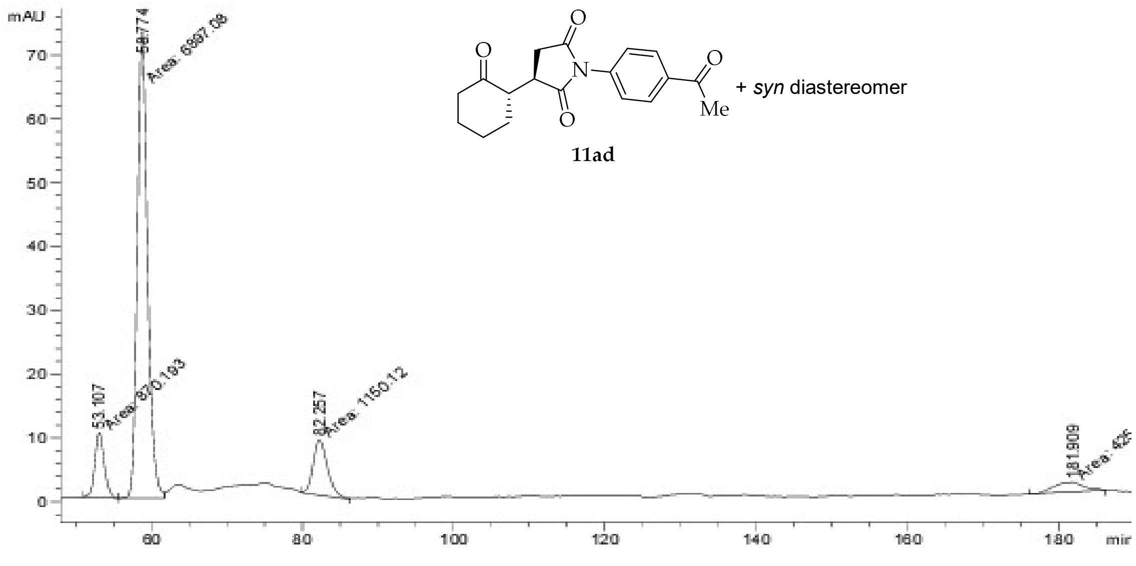

Peak #	RetTime [min]	Type	Width [min]	Area [mAU*s]	Height [mAU]	Area %
1	53.107	MM	1.4278	870.19257	10.15756	9.3139
2	58.774	MM	1.5721	6897.07666	73.11771	73.8216
3	82.257	MM	2.2135	1150.11829	8.65995	12.3101
4	181.909	MM	4.6978	425.51044	1.50961	4.5544

Figure S50. HPLC chromatogram of compound **11ad**.

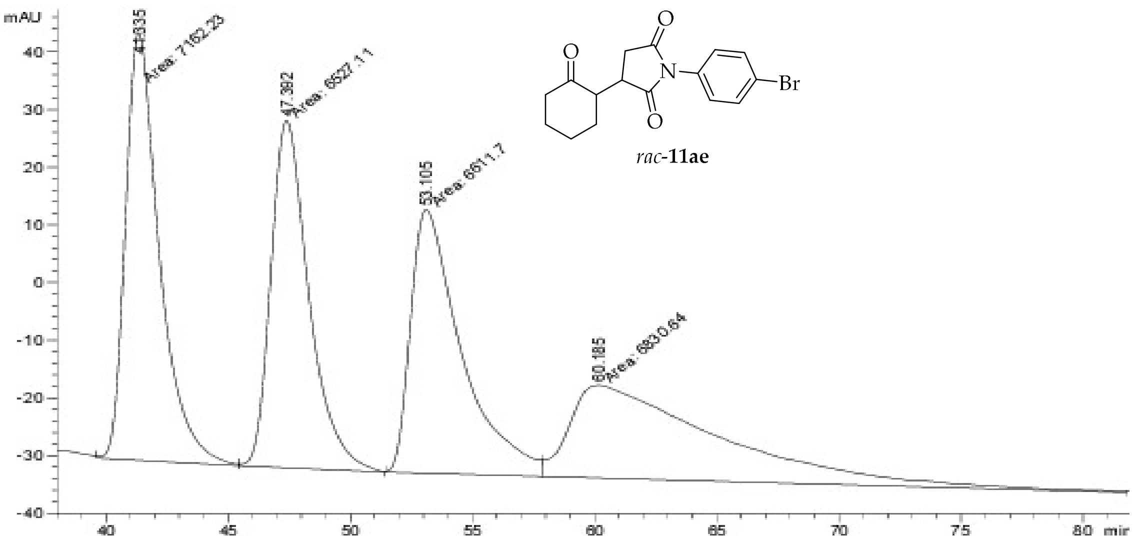

Peak #	RetTime [min]	Type	Width [min]	Area [mAU*s]	Height [mAU]	Area %
1	41.335	MM	1.6089	7162.22559	74.19167	26.3980
2	47.392	MM	1.8054	6527.10840	60.25534	24.0571
3	53.105	MF	2.4131	6611.70410	45.66485	24.3689
4	60.185	FM	7.0907	6830.64014	16.05532	25.1759

Figure S51. HPLC chromatogram of compound *rac*-**11ae**.

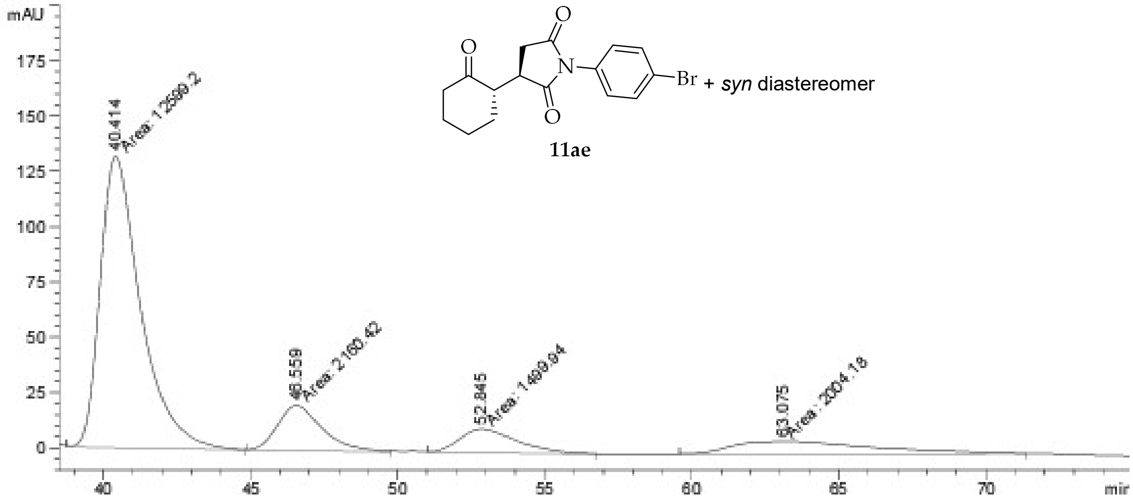

Peak #	RetTime [min]	Type	Width [min]	Area [mAU*s]	Height [mAU]	Area %
1	40.414	MM	1.5928	1.25992e4	131.83543	68.9848
2	46.559	MM	1.7679	2160.41992	20.36714	11.8290
3	52.845	MM	2.3155	1499.93665	10.79642	8.2126
4	63.075	MM	5.6162	2004.17737	5.94761	10.9735

Figure S52. HPLC chromatogram of compound **11ae**.

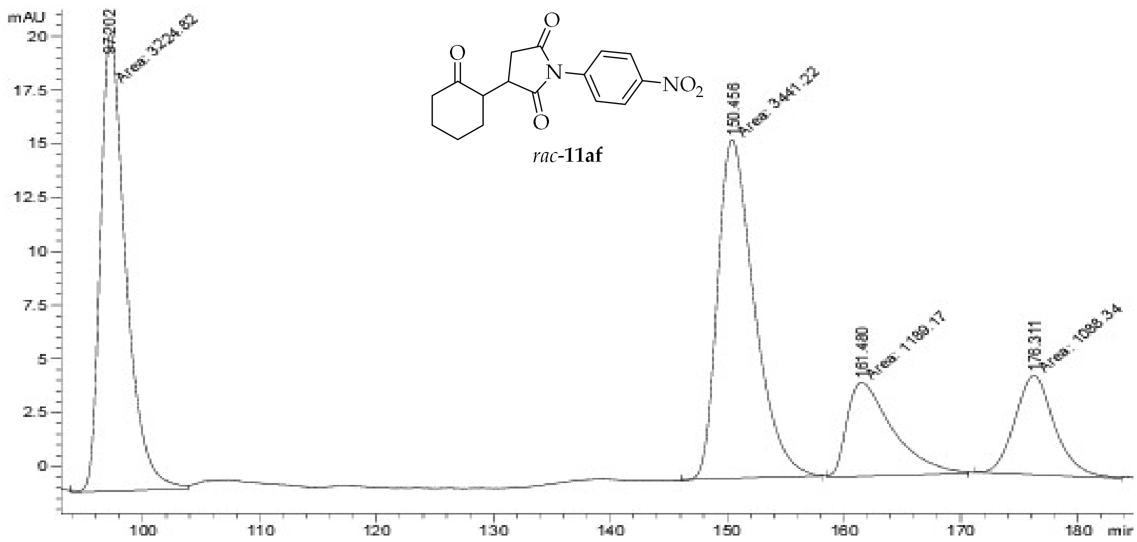

Peak #	RetTime [min]	Type	Width [min]	Area [mAU*s]	Height [mAU]	Area %
1	97.202	MM	2.5198	3224.82251	21.32964	36.0575
2	150.456	MM	3.6404	3441.21606	15.75481	38.4771
3	161.480	MM	4.5415	1189.17212	4.36409	13.2964
4	176.311	MM	3.9247	1088.33948	4.62174	12.1690

Figure S53. HPLC chromatogram of compound *rac*-**11af**.

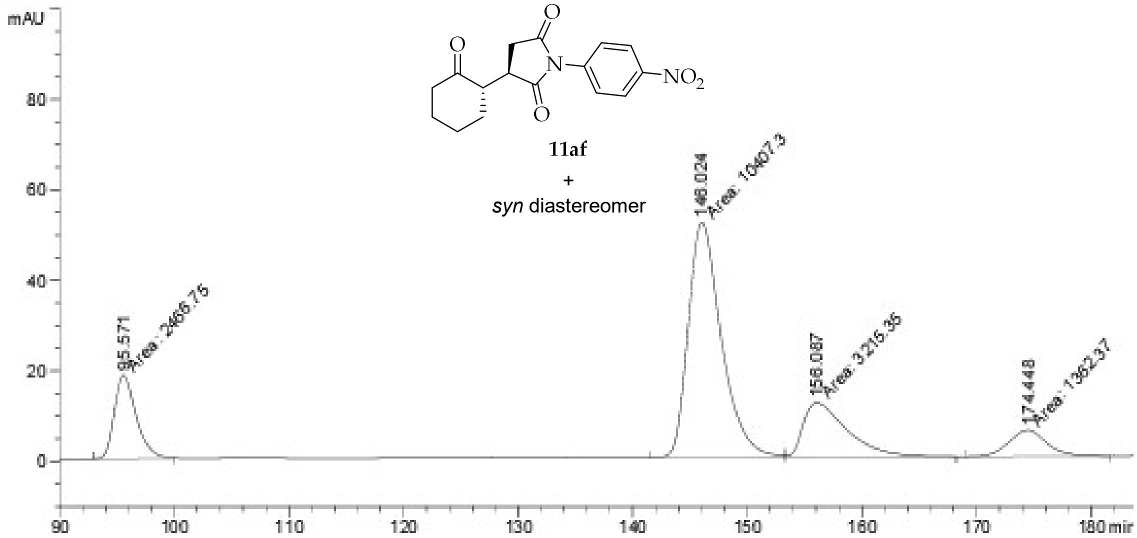

Peak #	RetTime [min]	Type	Width [min]	Area [mAU*s]	Height [mAU]	Area %
1	95.571	MM	2.2152	2466.74731	18.55913	14.1346
2	146.024	MF	3.3438	1.04073e4	51.87420	59.6347
3	156.087	FM	4.4572	3215.35278	12.02310	18.4242
4	174.448	MM	3.8732	1362.36584	5.86234	7.8064

Figure S54. HPLC chromatogram of compound **11af**.

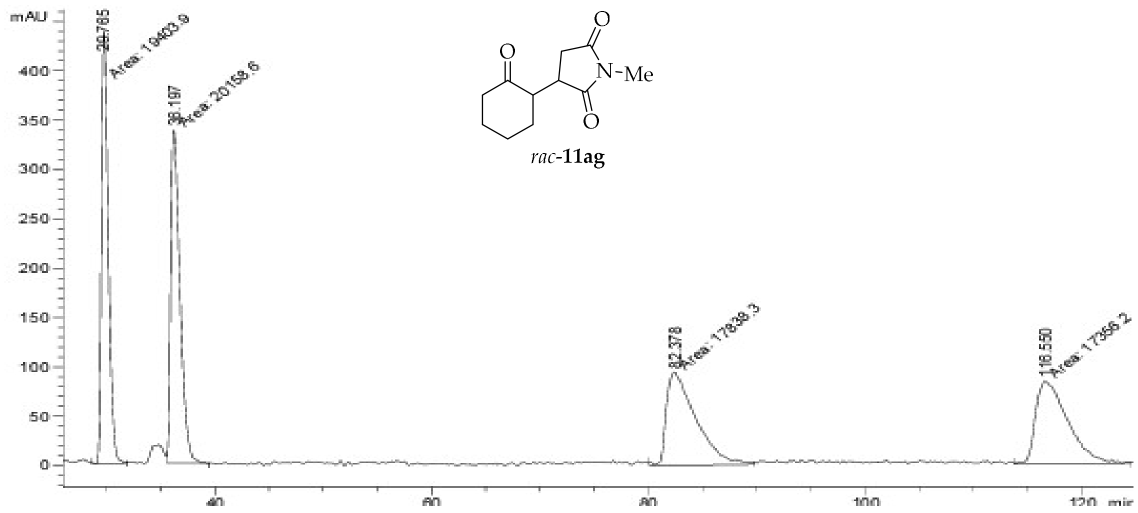

Peak #	RetTime [min]	Type	Width [min]	Area [mAU*s]	Height [mAU]	Area %
1	29.765	MM	0.7374	1.94039e4	438.58673	25.9560
2	36.197	FM	0.9957	2.01586e4	337.43411	26.9655
3	82.378	MM	3.1473	1.78383e4	94.46242	23.8617
4	116.550	MM	3.4319	1.73562e4	84.28939	23.2168

Figure S55. HPLC chromatogram of compound *rac*-**11ag**.

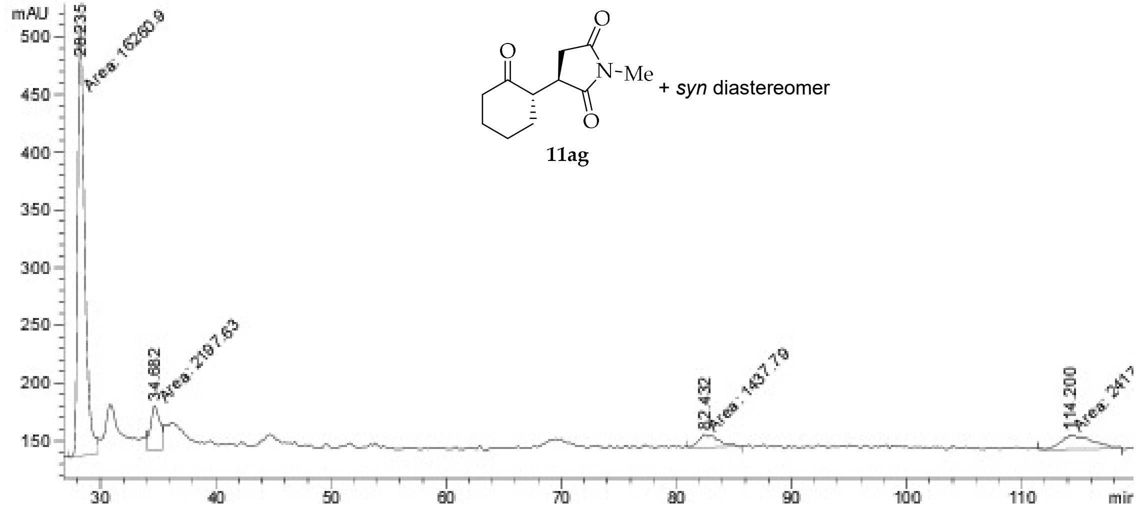

Peak #	RetTime [min]	Type	Width [min]	Area [mAU*s]	Height [mAU]	Area %
1	28.235	MM	0.7261	1.62609e4	373.23672	72.8740
2	34.682	MM	0.9432	2197.63135	38.83427	9.8488
3	82.432	MM	2.0220	1437.79211	11.85128	6.4435
4	114.200	MM	3.1701	2417.39868	12.70932	10.8337

Figure S56. HPLC chromatogram of compound **11ag**.

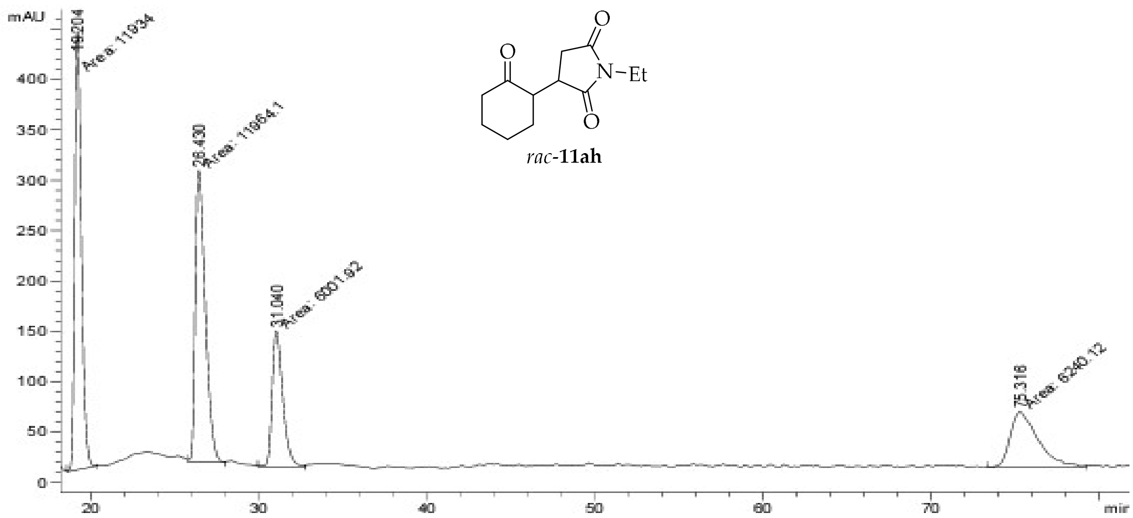

Peak #	RetTime [min]	Type	Width [min]	Area [mAU*s]	Height [mAU]	Area %
1	19.204	MM	0.4571	1.19340e4	435.16562	33.0214
2	26.430	MM	0.6882	1.19641e4	289.75534	33.1048
3	31.040	MM	0.7408	6001.91650	135.03879	16.6073
4	75.316	MM	1.9015	6240.11523	54.69476	17.2664

Figure S57. HPLC chromatogram of compound *rac*-**11ah**.

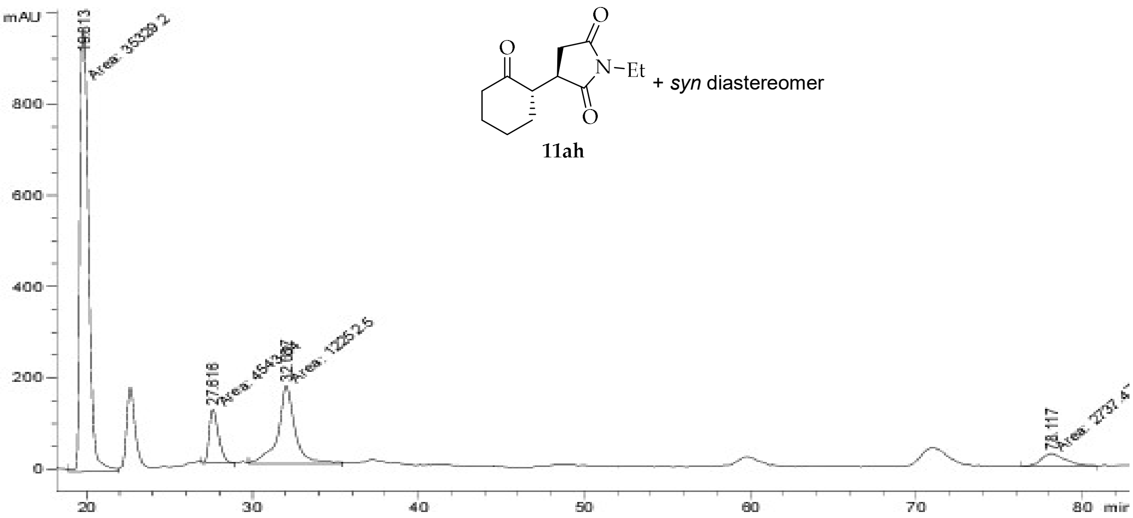

Peak #	RetTime [min]	Type	Width [min]	Area [mAU*s]	Height [mAU]	Area %
1	19.813	MM	0.6103	3.53292e4	964.76843	64.3963
2	27.616	MM	0.6403	4543.04492	118.24818	8.2808
3	32.037	MM	1.1954	1.22525e4	170.82472	22.3332
4	78.117	MM	1.6943	2737.47412	26.92798	4.9897

Figure S58. HPLC chromatogram of compound **11ah**.

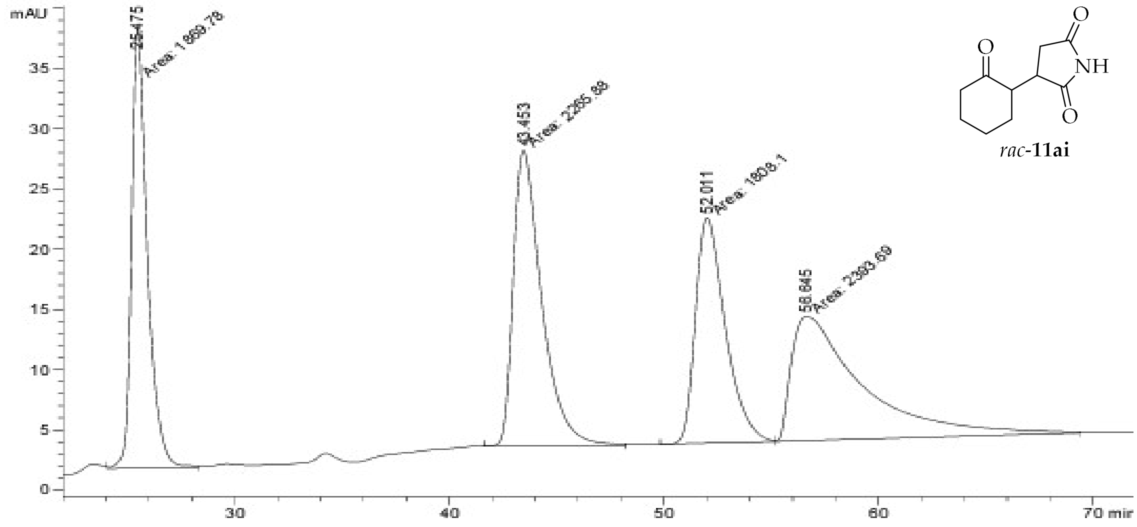

Peak #	RetTime [min]	Type	Width [min]	Area [mAU*s]	Height [mAU]	Area %
1	25.475	MM	0.8539	1869.77734	36.49651	22.4263
2	43.453	MM	1.5384	2265.87646	24.54864	27.1771
3	52.011	MM	1.6111	1808.09961	18.70457	21.6865
4	56.645	MM	3.8589	2393.69263	10.33835	28.7101

Figure S59. HPLC chromatogram of compound *rac*-**11ai**.

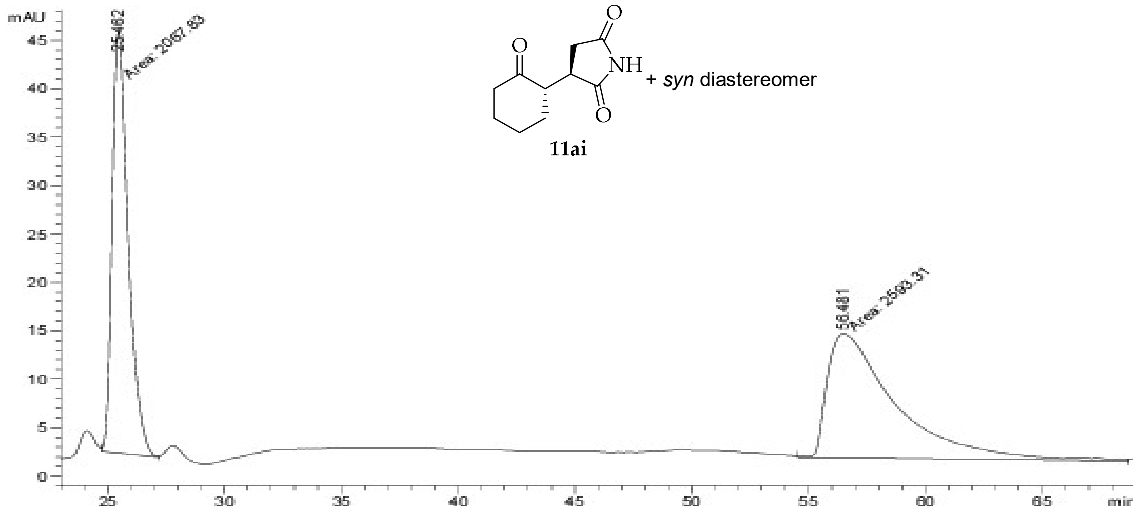

Peak #	RetTime [min]	Type	Width [min]	Area [mAU*s]	Height [mAU]	Area %
1	25.462	MM	0.7921	2067.83130	43.51075	44.3632
2	56.481	MM	3.3836	2593.31201	12.77402	55.6368

Figure S60. HPLC chromatogram of compound **11ai**.

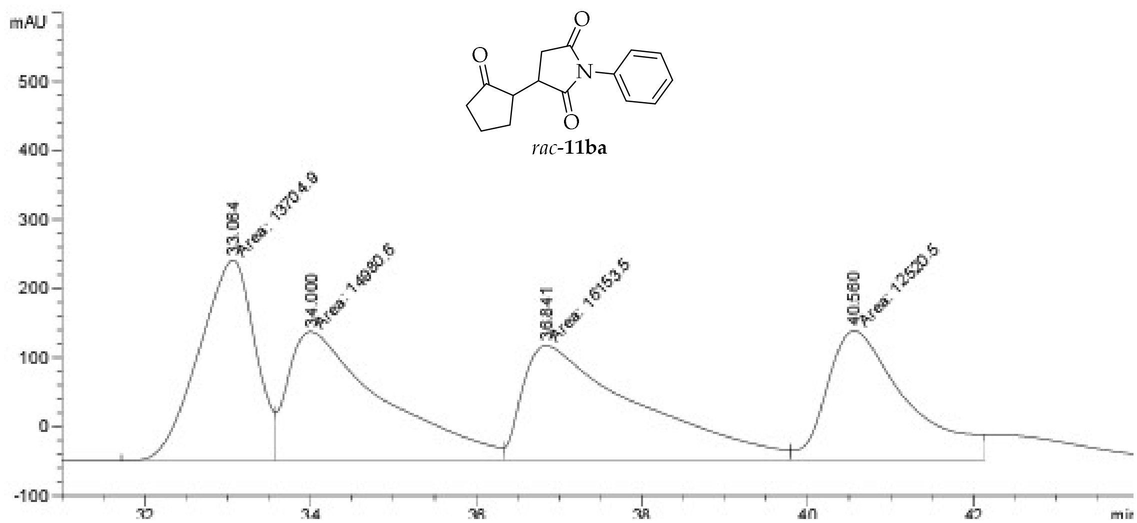

Peak #	RetTime [min]	Type	Width [min]	Area [mAU*s]	Height [mAU]	Area %
1	33.064	MF	0.7848	1.37049e4	291.06421	23.8930
2	34.000	MF	1.3326	1.49806e4	187.35893	26.1171
3	36.841	MF	1.6140	1.61535e4	166.80431	28.1618
4	40.560	FM	1.1097	1.25205e4	188.04073	21.8281

Figure S61. HPLC chromatogram of compound *rac*-**11ba**.

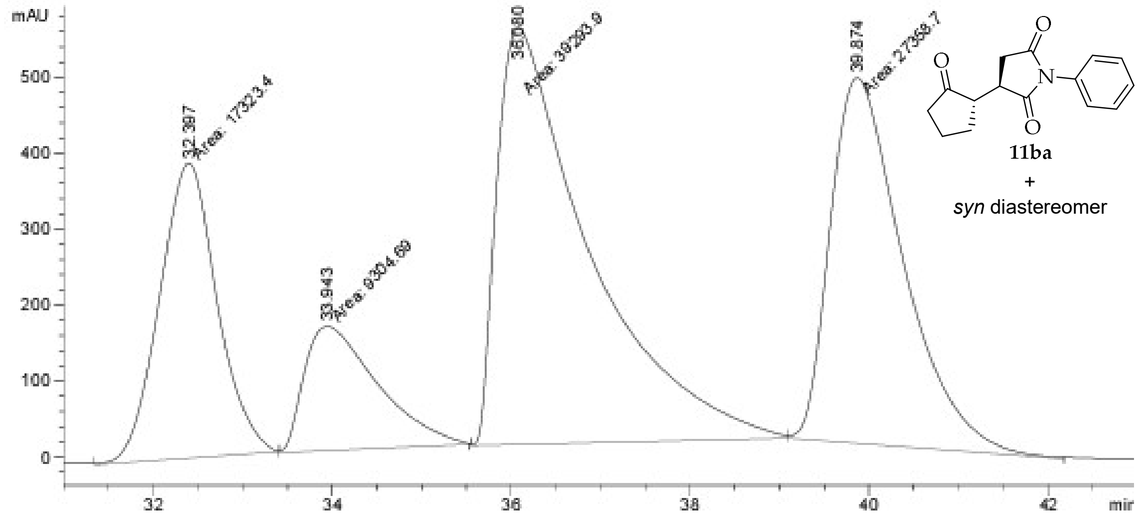

Peak #	RetTime [min]	Type	Width [min]	Area [mAU*s]	Height [mAU]	Area %
1	32.397	MM	0.7435	1.73234e4	388.33466	18.5713
2	33.943	MM	0.9463	9304.69336	163.88423	9.9749
3	36.080	MM	1.1945	3.92939e4	548.26361	42.1244
4	39.874	MM	0.9474	2.73587e4	481.31326	29.3294

Figure S62. HPLC chromatogram of compound **11ba**.

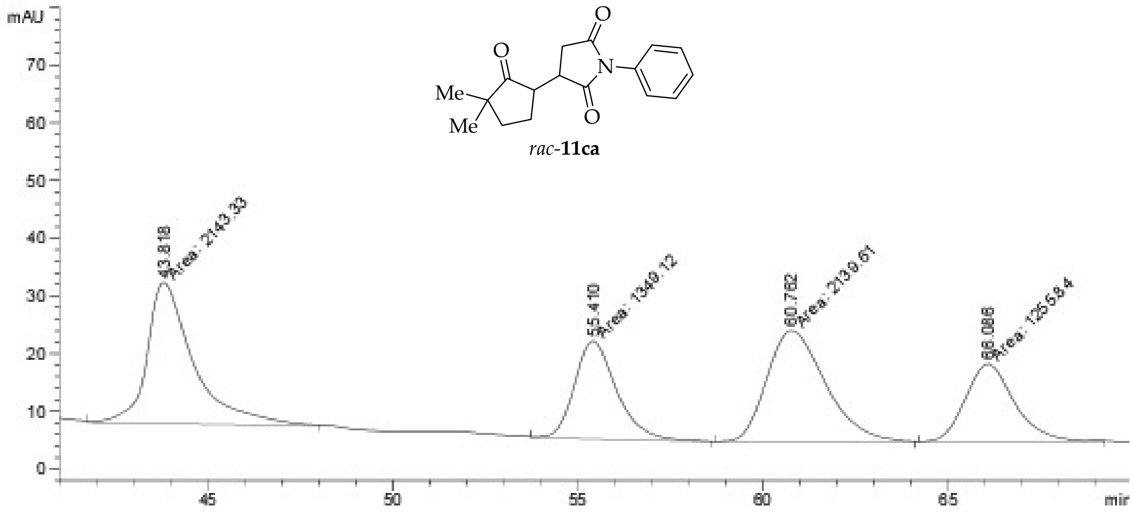

Peak #	RetTime [min]	Type	Width [min]	Area [mAU*s]	Height [mAU]	Area %
1	43.818	MM	1.4618	2143.33496	24.43648	31.1174
2	55.410	MM	1.3227	1349.12183	17.00022	19.5868
3	60.762	MM	1.8426	2139.60864	19.35271	31.0633
4	66.086	MM	1.5503	1255.84338	13.50132	18.2326

Figure S63. HPLC chromatogram of compound *rac*-**11ca**.

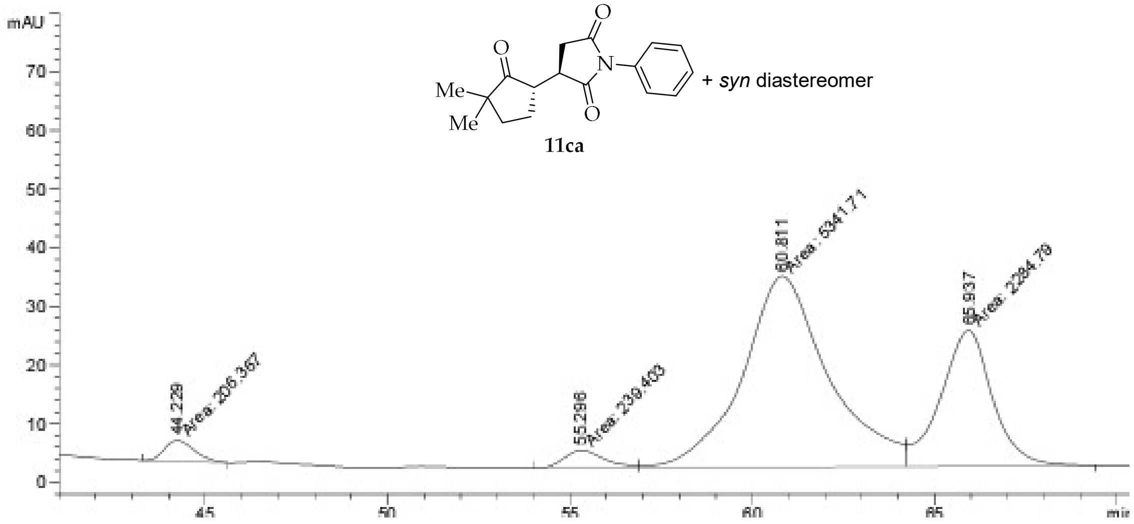

Peak #	RetTime [min]	Type	Width [min]	Area [mAU*s]	Height [mAU]	Area %
1	44.229	MM	0.9579	206.36650	3.59069	2.5565
2	55.296	MF	1.3084	239.40324	3.04949	2.9658
3	60.811	MF	2.7356	5341.70557	32.54422	66.1736
4	65.937	FM	1.6416	2284.78564	23.19720	28.3042

Figure S64. HPLC chromatogram of compound **11ca**.

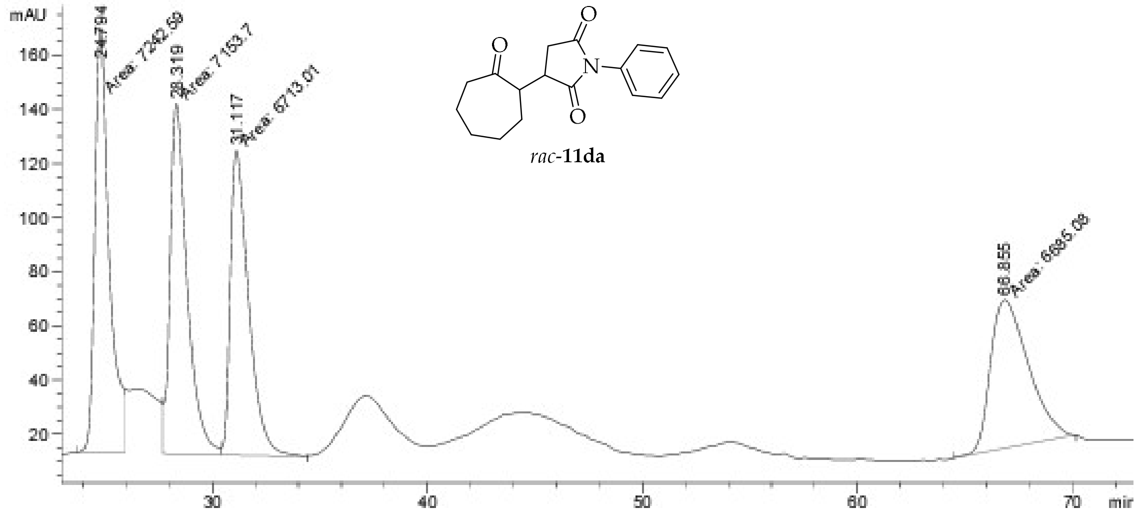

Peak #	RetTime [min]	Type	Width [min]	Area [mAU*s]	Height [mAU]	Area %
1	24.794	MF	0.7682	7242.58691	157.12639	26.0577
2	28.319	MF	0.9215	7153.70215	129.37955	25.7379
3	31.117	FM	0.9943	6713.01123	112.52296	24.1524
4	66.855	MM	2.0329	6685.08350	54.80653	24.0519

Figure S65. HPLC chromatogram of compound *rac*-**11da**.

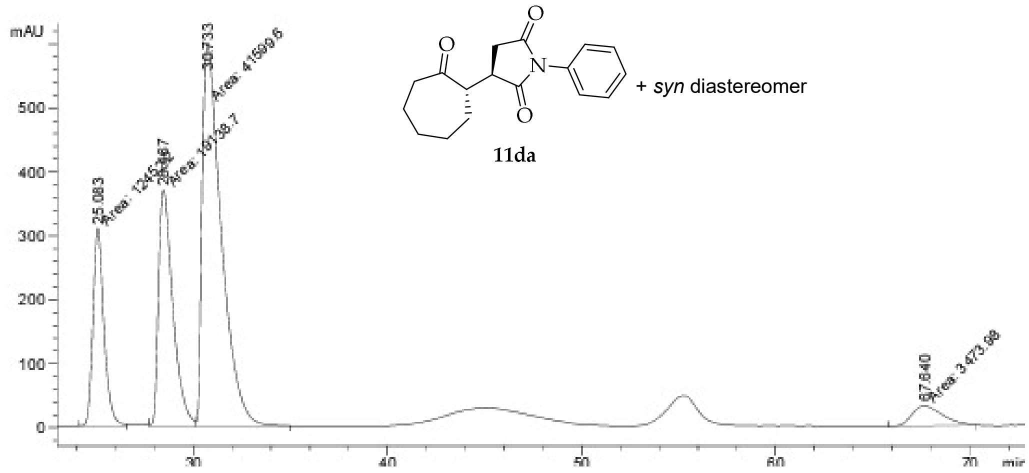

Peak #	RetTime [min]	Type	Width [min]	Area [mAU*s]	Height [mAU]	Area %
1	25.083	MF	0.6676	1.24522e4	310.88812	16.2424
2	28.467	MF	0.8615	1.91387e4	370.24838	24.9643
3	30.733	FM	1.1522	4.15996e4	601.73669	54.2619
4	67.640	MM	1.8314	3473.97852	31.61424	4.5314

Figure S66. HPLC chromatogram of compound **11da**.

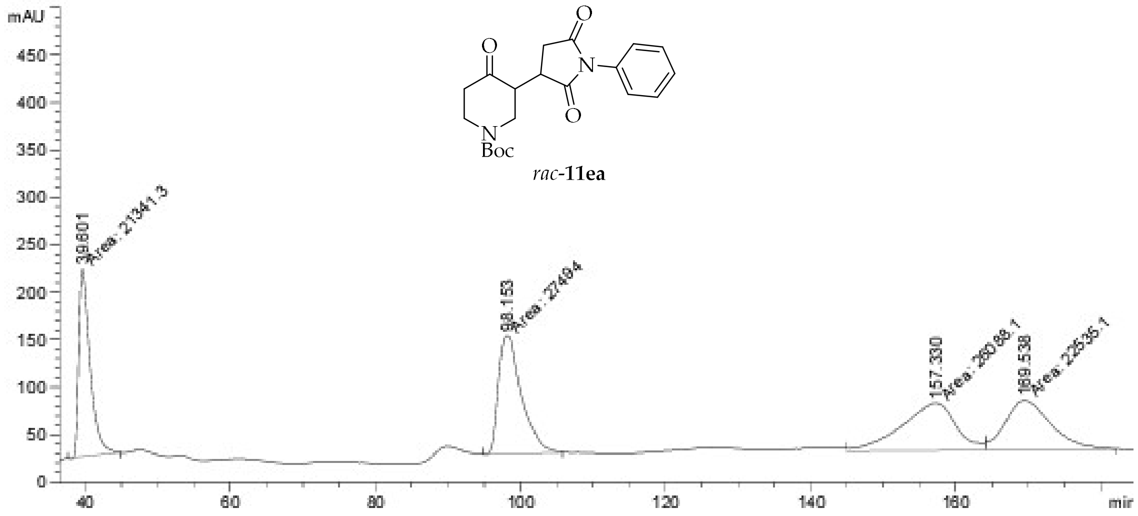

Peak #	RetTime [min]	Type	Width [min]	Area [mAU*s]	Height [mAU]	Area %
1	39.601	MM	1.8007	2.13413e4	197.52512	21.8978
2	98.153	MM	3.6761	2.74940e4	124.65079	28.2110
3	157.330	MF	8.7371	2.60881e4	49.76489	26.7684
4	169.538	FM	7.2377	2.25351e4	51.89299	23.1227

Figure S67. HPLC chromatogram of compound *rac*-**11ea**.

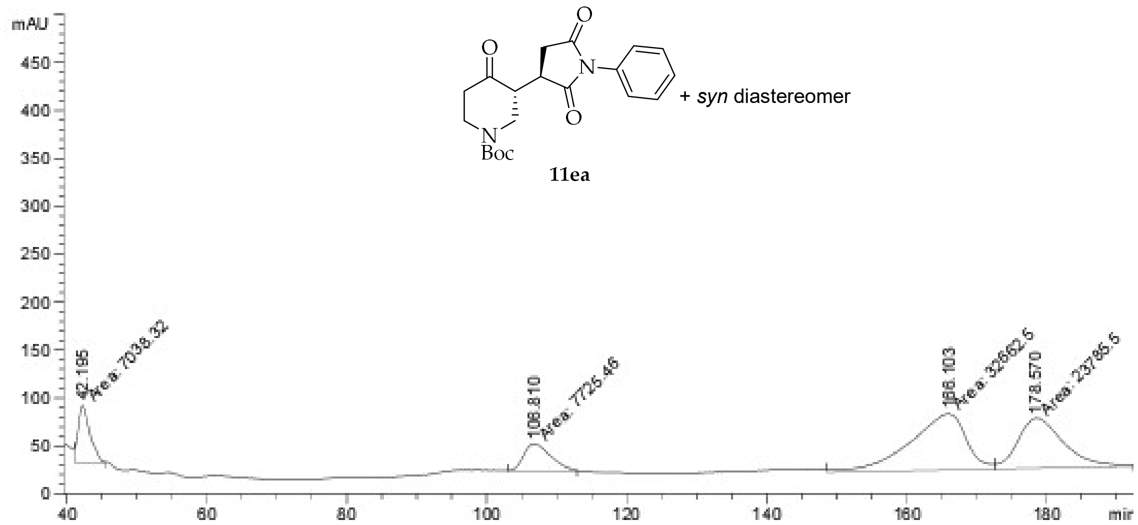

Peak #	RetTime [min]	Type	Width [min]	Area [mAU*s]	Height [mAU]	Area %
1	42.195	MM	1.9759	7038.32373	59.36661	9.8836
2	106.810	MM	4.5094	7725.45557	28.55336	10.8486
3	166.103	MF	9.3078	3.26625e4	58.48572	45.8667
4	178.570	FM	7.6155	2.37855e4	52.05519	33.4011

Figure S68. HPLC chromatogram of compound **11ea**.

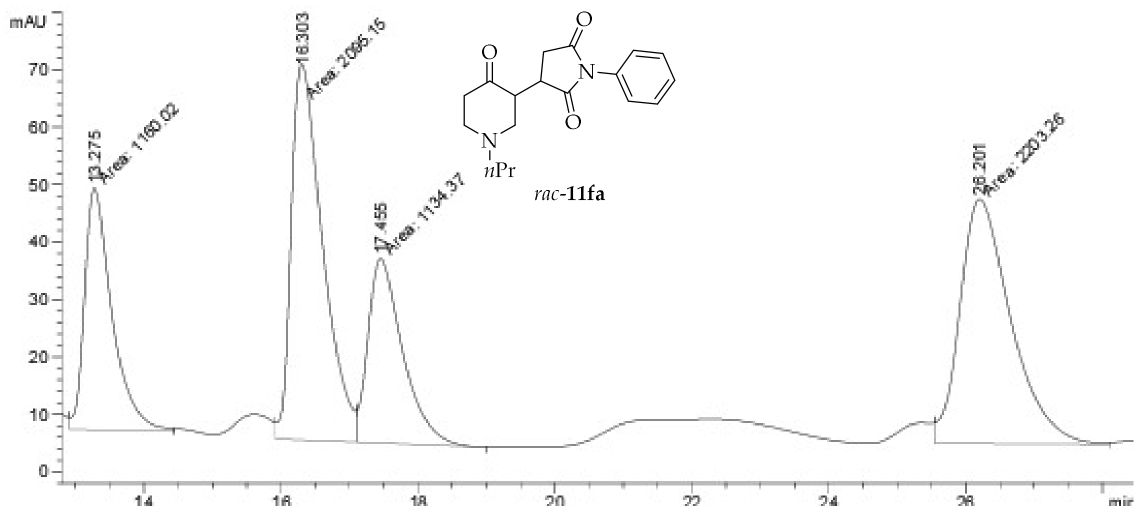

Peak #	RetTime [min]	Type	Width [min]	Area [mAU*s]	Height [mAU]	Area %
1	13.275	MM	0.4597	1160.01953	42.05348	17.5952
2	16.303	MF	0.5333	2095.14502	65.48257	31.7793
3	17.455	FM	0.5887	1134.37354	32.11398	17.2062
4	26.201	FM	0.8631	2203.26147	42.54479	33.4192

Figure S69. HPLC chromatogram of compound *rac*-**11fa**.

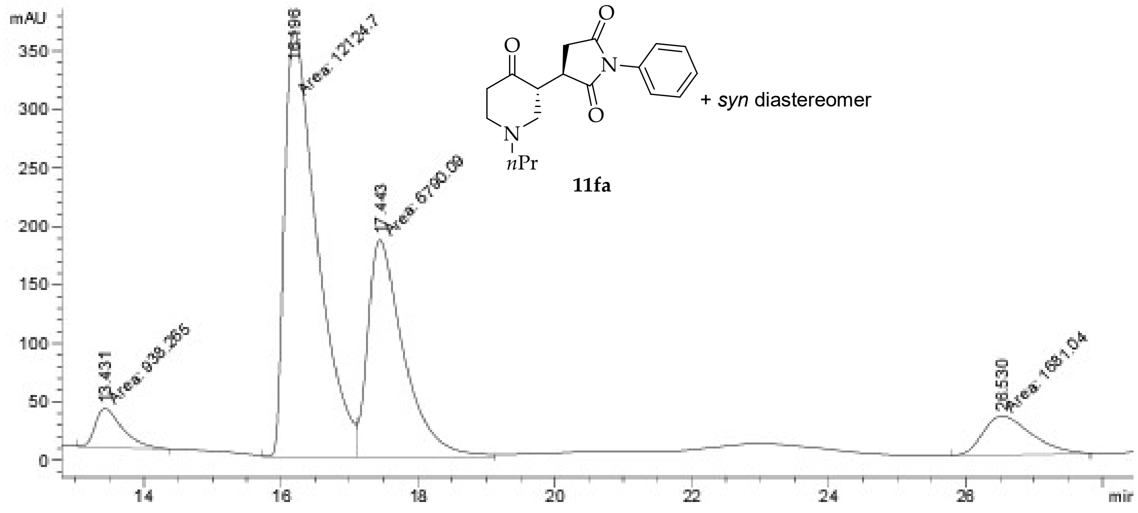

Peak #	RetTime [min]	Type	Width [min]	Area [mAU*s]	Height [mAU]	Area %
1	13.431	MM	0.4662	938.26538	33.54221	4.3571
2	16.196	MF	0.5529	1.21247e4	365.48090	56.3046
3	17.443	FM	0.6077	6790.09473	186.23572	31.5319
4	26.530	MM	0.8354	1681.03662	33.53699	7.8064

Figure S70. HPLC chromatogram of compound **11fa**.

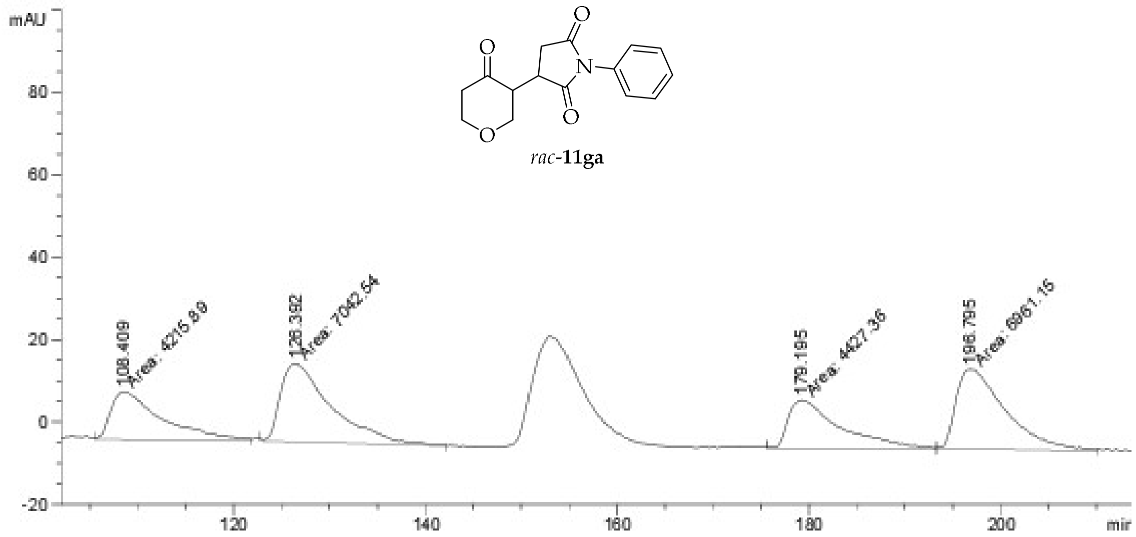

Peak #	RetTime [min]	Type	Width [min]	Area [mAU*s]	Height [mAU]	Area %
1	108.409	MM	6.0390	4215.88818	11.63520	18.6157
2	126.392	MM	6.2003	7042.54053	18.93077	31.0971
3	179.195	MM	6.2887	4427.35693	11.73372	19.5495
4	196.795	MM	5.9836	6961.14600	19.38960	30.7377

Figure S71. HPLC chromatogram of compound *rac*-**11ga**.

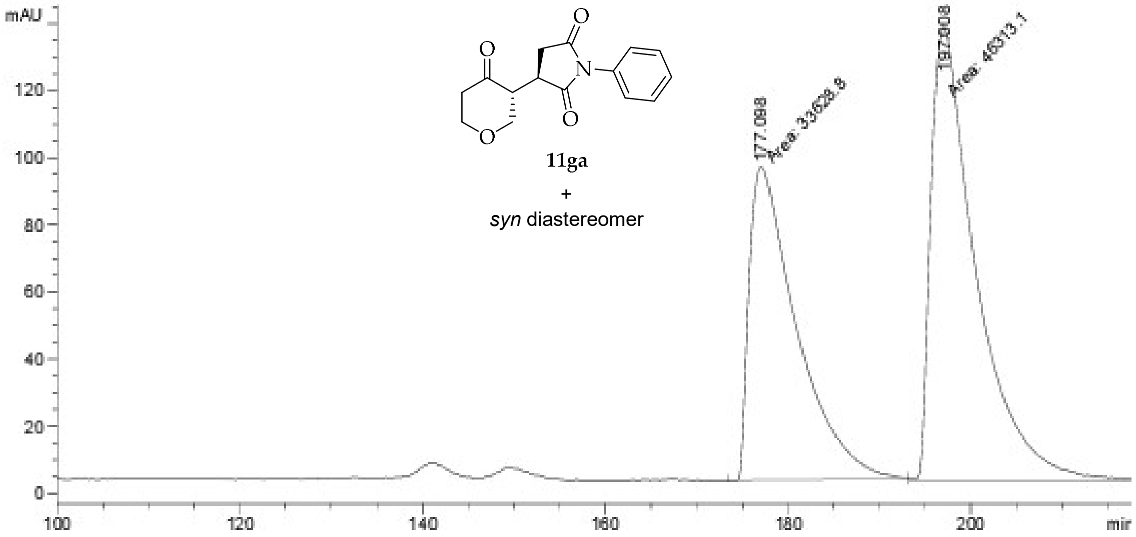

Peak #	RetTime [min]	Type	Width [min]	Area [mAU*s]	Height [mAU]	Area %
1	177.098	MM	6.0272	3.36288e4	92.99184	42.0665
2	197.008	MM	5.7294	4.63131e4	134.72319	57.9335

Figure S72. HPLC chromatogram of compound **11ga**.

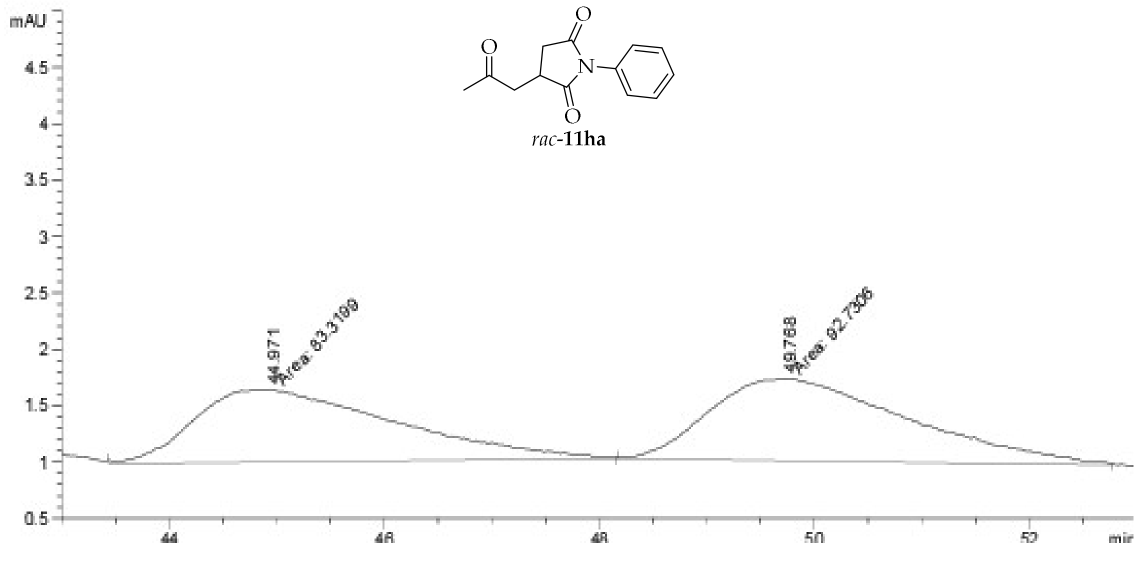

Peak #	RetTime [min]	Type	Width [min]	Area [mAU*s]	Height [mAU]	Area %
1	44.971	MM	2.1756	83.31993	6.38295e-1	47.3273
2	49.768	MM	2.1168	92.73058	7.30117e-1	52.6727

Figure S73. HPLC chromatogram of compound *rac*-**11ha**.

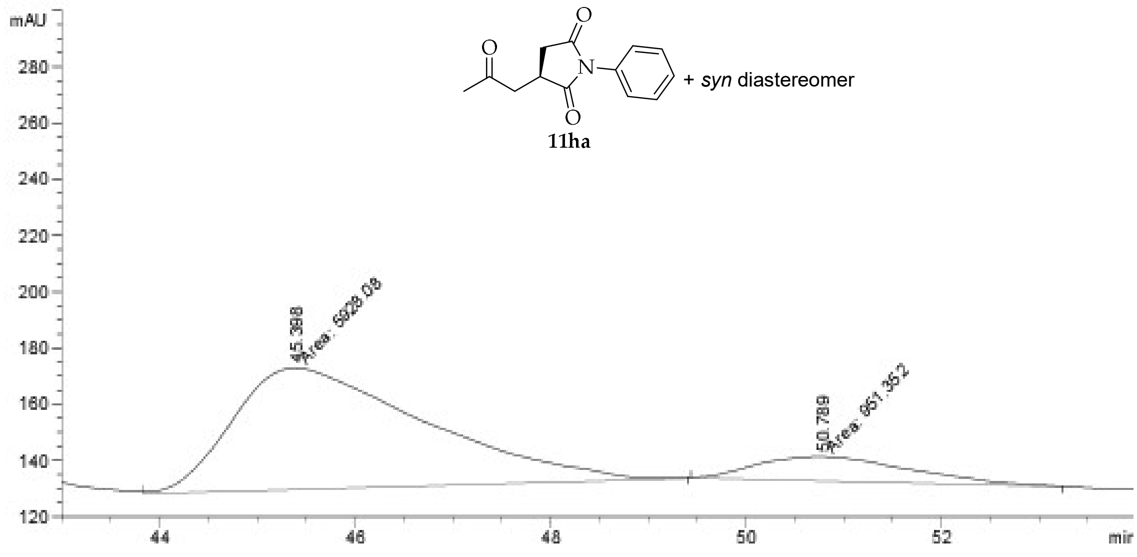

Peak #	RetTime [min]	Type	Width [min]	Area [mAU*s]	Height [mAU]	Area %
1	45.398	MM	2.2884	5928.07568	43.17566	86.1711
2	50.789	MM	1.8325	951.35242	8.65269	13.8289

Figure S74. HPLC chromatogram of compound **11ha**.

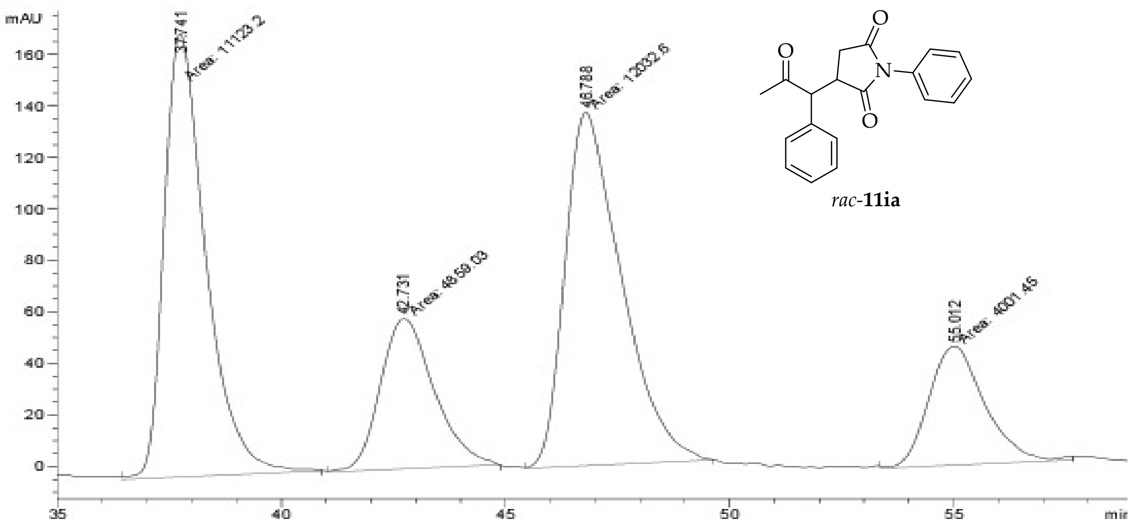

Peak #	RetTime [min]	Type	Width [min]	Area [mAU*s]	Height [mAU]	Area %
1	37.741	MM	1.0762	1.11232e4	172.25964	34.7422
2	42.731	MM	1.3901	4859.03027	58.25960	15.1768
3	46.788	MM	1.4610	1.20326e4	137.26492	37.5828
4	55.012	MM	1.4449	4001.44653	46.15499	12.4982

Figure S75. HPLC chromatogram of compound *rac*-**11ia**.

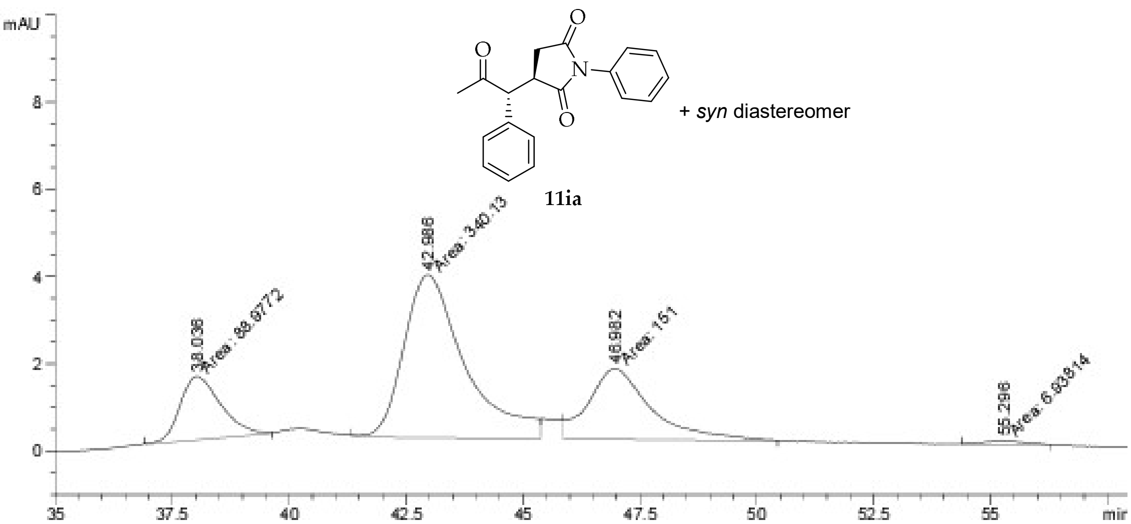

Peak #	RetTime [min]	Type	Width [min]	Area [mAU*s]	Height [mAU]	Area %
1	38.036	MM	1.0142	88.97725	1.46219	15.1568
2	42.986	MF	1.5191	340.12952	3.73181	57.9393
3	46.982	FM	1.5571	150.99973	1.61622	25.7220
4	55.296	MM	1.1483	6.93814	1.00703e-1	1.1819

Figure S76. HPLC chromatogram of compound **11ia**.

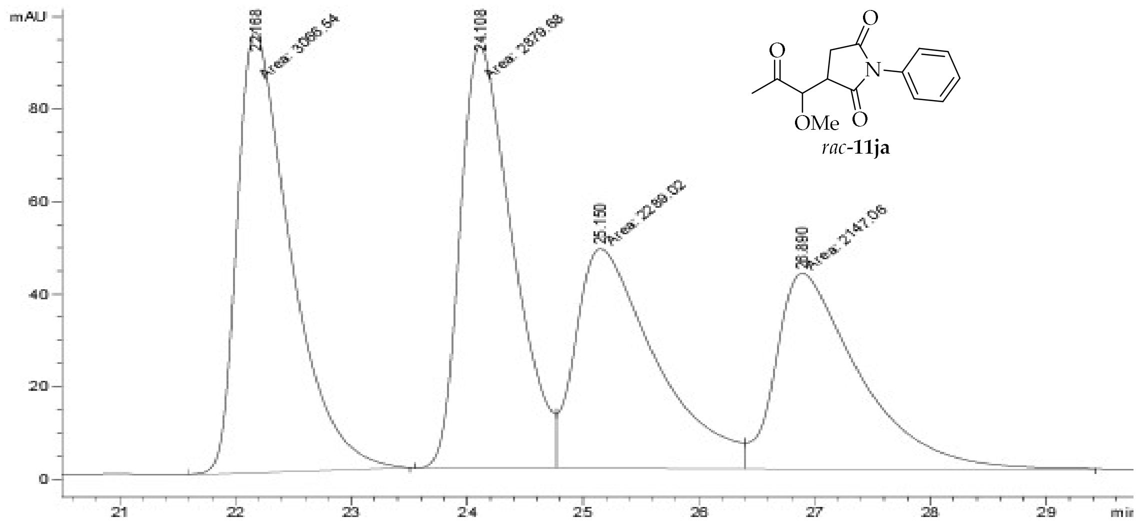

Peak #	RetTime [min]	Type	Width [min]	Area [mAU*s]	Height [mAU]	Area %
1	22.168	MM	0.5358	3066.54150	95.39330	29.5362
2	24.108	MF	0.5266	2879.68188	91.14449	27.7364
3	25.150	MF	0.8037	2289.02393	47.46743	22.0473
4	26.890	FM	0.8462	2147.06226	42.28981	20.6800

Figure S77. HPLC chromatogram of compound *rac*-**11ja**.

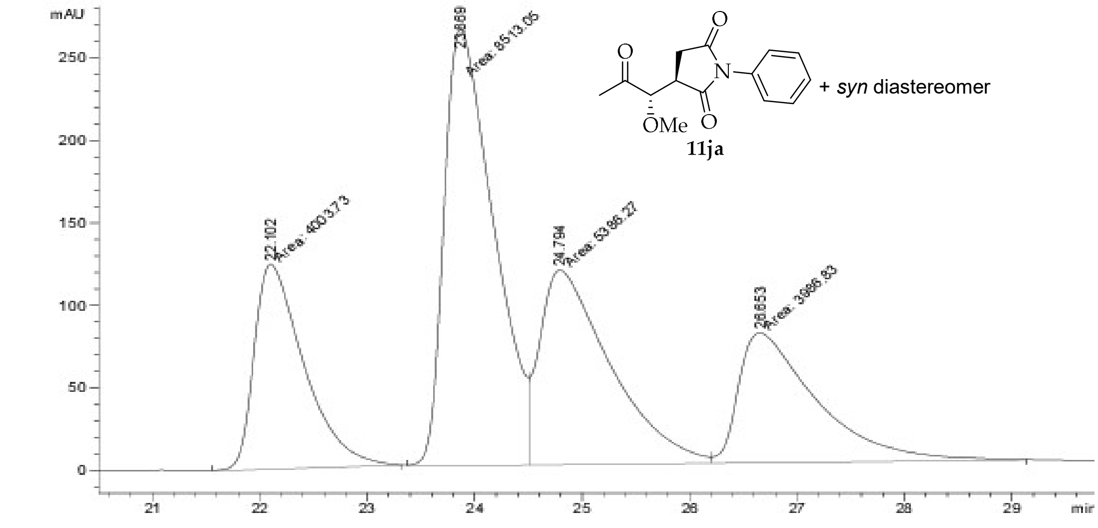

Peak #	RetTime [min]	Type	Width [min]	Area [mAU*s]	Height [mAU]	Area %
1	22.102	MM	0.5368	4003.73315	124.31665	18.2903
2	23.869	MF	0.5357	8513.05273	264.87021	38.8904
3	24.794	MF	0.7611	5386.26709	117.94196	24.6062
4	26.653	FM	0.8435	3986.83032	78.77255	18.2131

Figure S78. HPLC chromatogram of compound **11ja**.

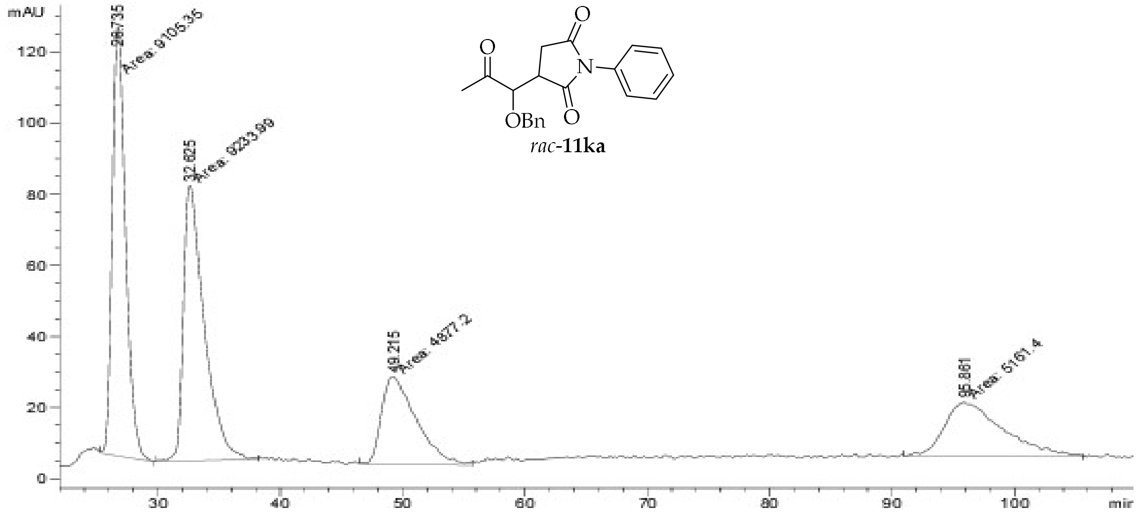

Peak #	RetTime [min]	Type	Width [min]	Area [mAU*s]	Height [mAU]	Area %
1	26.735	MM	1.2567	9105.34961	120.76128	32.0860
2	32.625	MM	1.9859	9233.99414	77.49511	32.5393
3	49.215	MM	3.2849	4877.20361	24.74577	17.1866
4	95.861	MM	5.5841	5161.39893	15.40511	18.1881

Figure S79. HPLC chromatogram of compound *rac*-**11ka**.

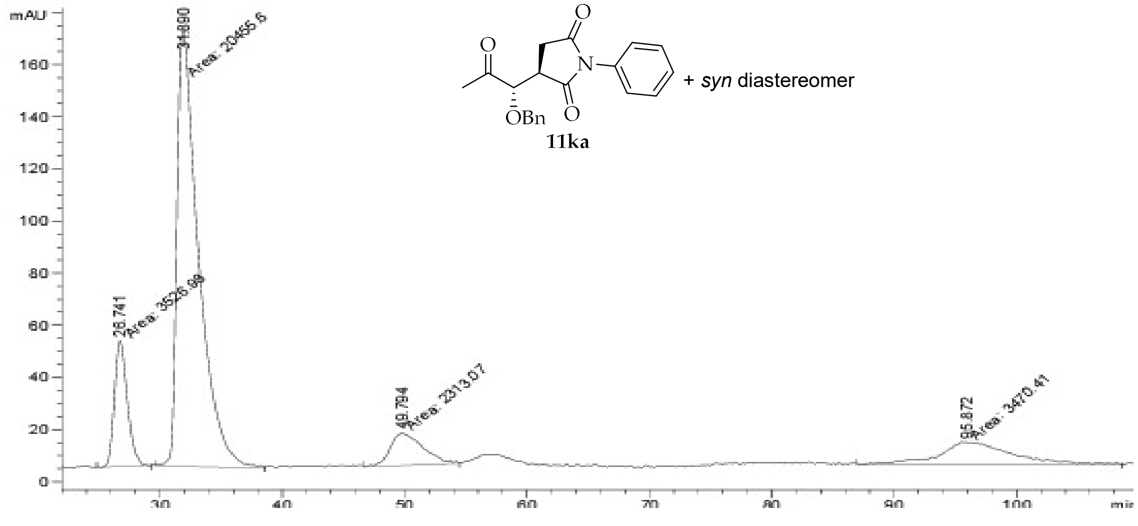

Peak #	RetTime [min]	Type	Width [min]	Area [mAU*s]	Height [mAU]	Area %
1	26.741	MM	1.2192	3526.97998	48.21417	11.8490
2	31.890	MM	2.0365	2.04556e4	167.41229	68.7212
3	49.794	MM	3.0551	2313.07349	12.61871	7.7708
4	95.872	MM	6.7771	3470.40771	8.53461	11.6589

Figure S80. HPLC chromatogram of compound **11ka**.

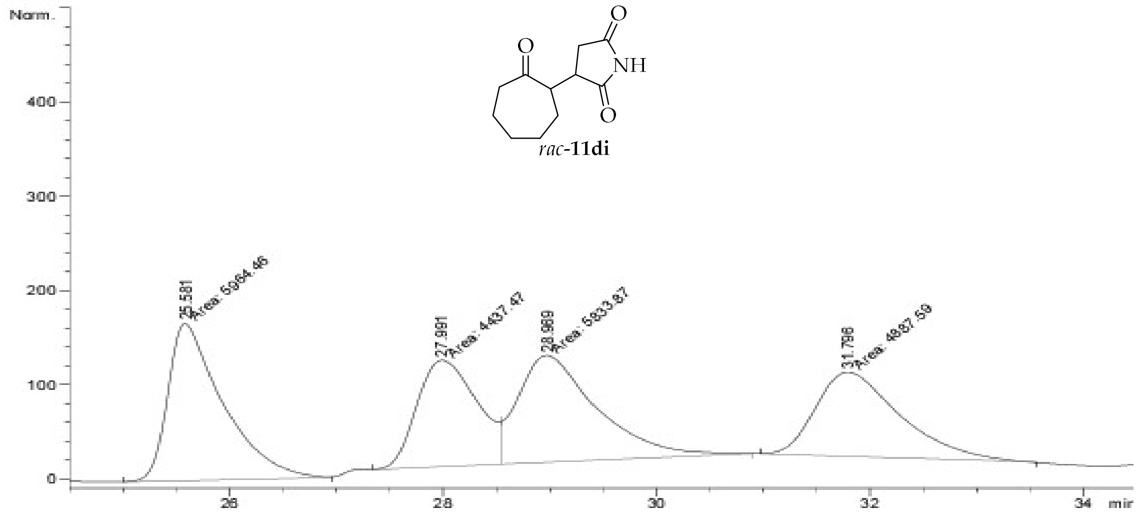

Peak #	RetTime [min]	Type	Width [min]	Area [mAU*s]	Height [mAU]	Area %
1	25.581	MM	0.5972	5964.46191	166.46904	28.2363
2	27.991	MF	0.6544	4437.47412	113.01321	21.0074
3	28.969	FM	0.8581	5833.86523	113.30320	27.6180
4	31.796	MM	0.9102	4887.58838	89.49586	23.1383

Figure S81. HPLC chromatogram of compound *rac*-**11di**.

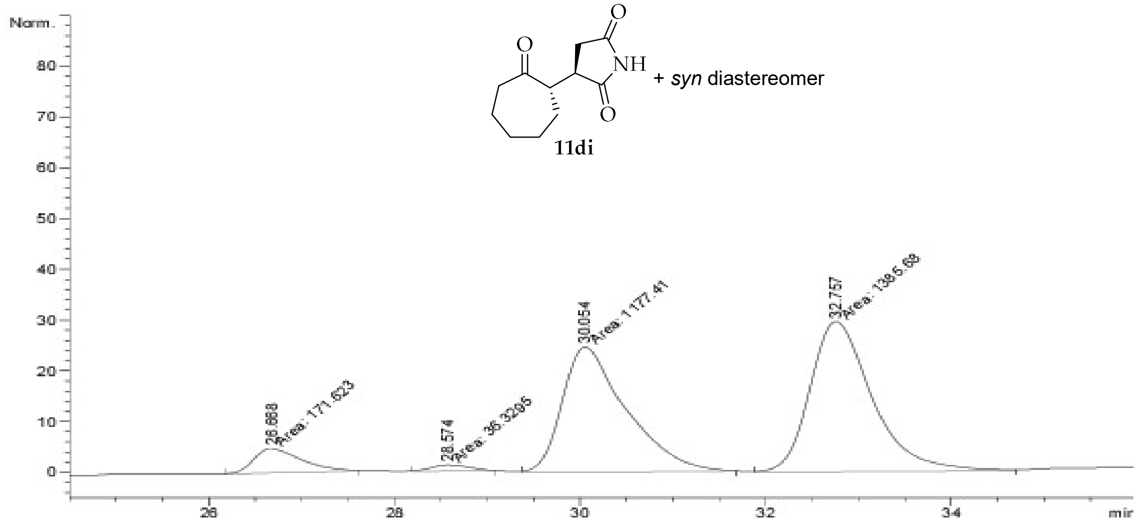

Peak #	RetTime [min]	Type	Width [min]	Area [mAU*s]	Height [mAU]	Area %
1	26.668	MM	0.5925	171.62267	4.82785	6.1934
2	28.574	MM	0.4926	36.32951	1.22912	1.3110
3	30.054	MM	0.7970	1177.40625	24.62033	42.4897
4	32.757	MM	0.7807	1385.68018	29.58154	50.0058

Figure S82. HPLC chromatogram of compound **11di**.

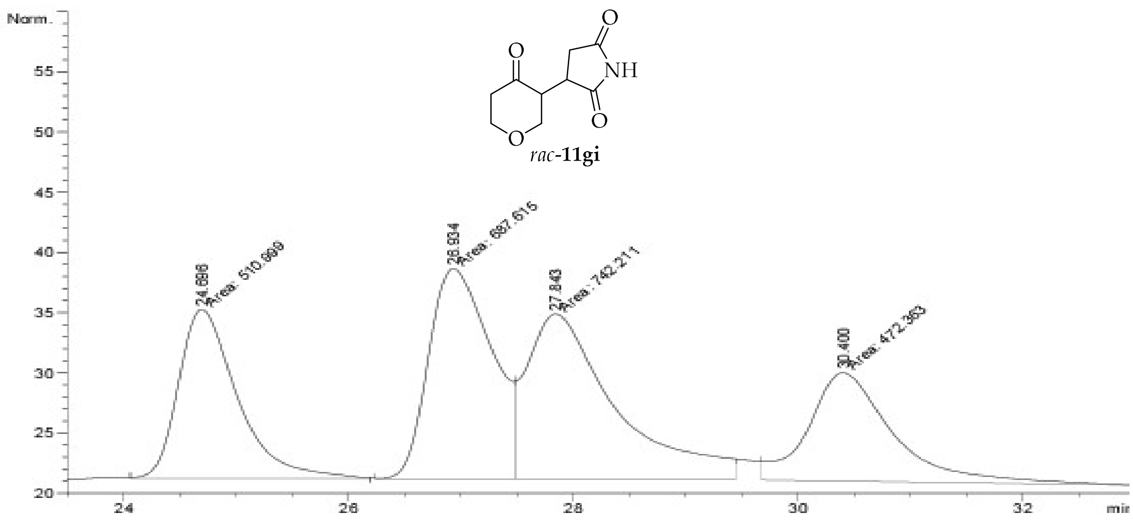

Peak #	RetTime [min]	Type	Width [min]	Area [mAU*s]	Height [mAU]	Area %
1	24.696	MM	0.6072	510.99866	14.02711	21.1753
2	26.934	MF	0.6562	687.61475	17.46419	28.4940
3	27.843	FM	0.9017	742.21075	13.71894	30.7565
4	30.400	MM	0.8754	472.36301	8.99343	19.5742

Figure S83. HPLC chromatogram of compound *rac*-**11gi**.

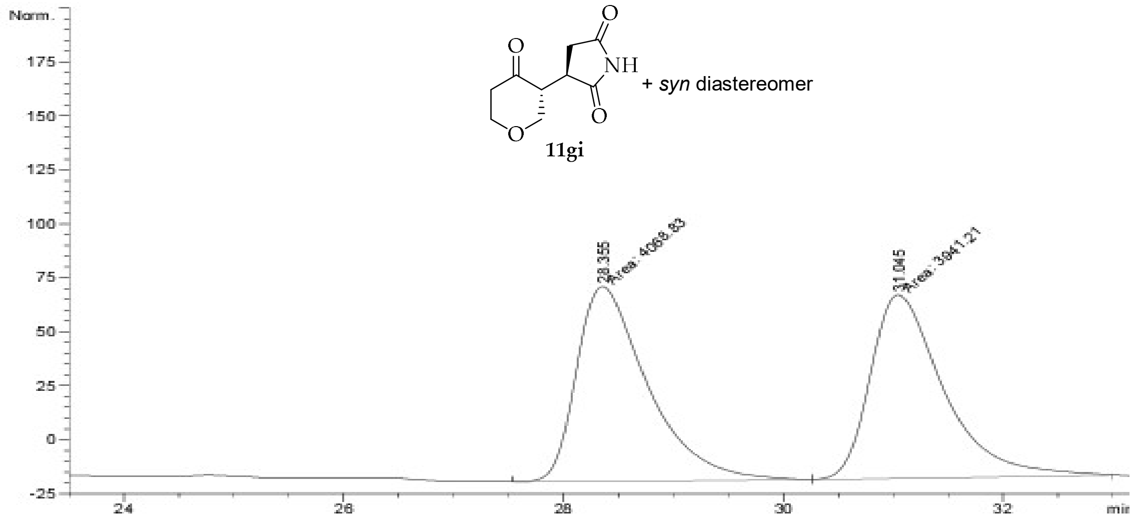

Peak #	RetTime [min]	Type	Width [min]	Area [mAU*s]	Height [mAU]	Area %
1	28.355	MM	0.7512	4068.82642	90.27445	50.7966
2	31.045	MM	0.7726	3941.20654	85.01845	49.2034

Figure S84. HPLC chromatogram of compound **11gi**.
